# *CERT1* mutations perturb human development by disrupting sphingolipid homeostasis

**DOI:** 10.1172/JCI165019

**Published:** 2023-03-28

**Authors:** Charlotte Gehin, Museer A. Lone, Winston Lee, Laura Capolupo, Sylvia Ho, Adekemi M. Adeyemi, Erica H. Gerkes, Alexander P.A. Stegmann, Estrella López-Martín, Eva Bermejo-Sánchez, Beatriz Martínez-Delgado, Christiane Zweier, Cornelia Kraus, Bernt Popp, Vincent Strehlow, Daniel Gräfe, Ina Knerr, Eppie R. Jones, Stefano Zamuner, Luciano A. Abriata, Vidya Kunnathully, Brandon E. Moeller, Anthony Vocat, Samuel Rommelaere, Jean-Philippe Bocquete, Evelyne Ruchti, Greta Limoni, Marine Van Campenhoudt, Samuel Bourgeat, Petra Henklein, Christian Gilissen, Bregje W. van Bon, Rolph Pfundt, Marjolein H. Willemsen, Jolanda H. Schieving, Emanuela Leonardi, Fiorenza Soli, Alessandra Murgia, Hui Guo, Qiumeng Zhang, Kun Xia, Christina R. Fagerberg, Christoph P. Beier, Martin J. Larsen, Irene Valenzuela, Paula Fernández-Álvarez, Shiyi Xiong, Robert Śmigiel, Vanesa López-González, Lluís Armengol, Manuela Morleo, Angelo Selicorni, Annalaura Torella, Moira Blyth, Nicola S. Cooper, Valerie Wilson, Renske Oegema, Yvan Herenger, Aurore Garde, Ange-Line Bruel, Frederic Tran Mau-Them, Alexis B.R. Maddocks, Jennifer M. Bain, Musadiq A. Bhat, Gregory Costain, Peter Kannu, Ashish Marwaha, Neena L. Champaigne, Michael J. Friez, Ellen B. Richardson, Vykuntaraju K. Gowda, Varunvenkat M. Srinivasan, Yask Gupta, Tze Y. Lim, Simone Sanna-Cherchi, Bruno Lemaitre, Toshiyuki Yamaji, Kentaro Hanada, John E. Burke, Ana Marija Jakšić, Brian D. McCabe, Paolo De Los Rios, Thorsten Hornemann, Giovanni D’Angelo, Vincenzo A. Gennarino

**Affiliations:** 1 Institute of Bioengineering (IBI), École Polytechnique Fédérale de Lausanne (EPFL), Lausanne, Switzerland.; 2 Institute of Clinical Chemistry, University Hospital Zurich, University of Zurich, Zurich, Switzerland.; 3 Department of Genetics and Development and; 4 Department Ophthalmology, Columbia University Irving Medical Center, New York, New York, USA.; 5 Department of Medical Genetics, Cumming School of Medicine, The University of Calgary, Calgary, Alberta, Canada.; 6 University of Groningen, University Medical Center Groningen, Department of Genetics, Groningen, Netherlands.; 7 Department of Clinical Genetics and School for Oncology and Developmental Biology (GROW), Maastricht University Medical Center, Maastricht, Netherlands.; 8 Institute of Rare Diseases Research (IIER), Instituto de Salud Carlos III, Madrid, Spain.; 9 Institute of Human Genetics, Friedrich-Alexander-Universität Erlangen-Nürnberg, Erlangen, Germany.; 10 Department of Human Genetics, Inselspital, Bern University Hospital, University of Bern, Bern, Switzerland.; 11 Institute of Human Genetics, University of Leipzig Medical Center, Leipzig, Germany.; 12 Berlin Institute of Health at Charité – Universitätsmedizin Berlin, Center of Functional Genomics, Berlin, Germany.; 13 Department of Pediatric Radiology, University Hospital Leipzig, Leipzig, Leipzig, Germany.; 14 National Centre for Inherited Metabolic Disorders, Children’s Health Ireland (CHI) at Temple Street, Dublin, Ireland.; 15 UCD School of Medicine, Dublin, Ireland.; 16 Genuity Science, Cherrywood Business Park, Dublin, Ireland.; 17 Institute of Physics, School of Basic Sciences, École Polytechnique Féderale de Lausanne (EPFL), Lausanne, Switzerland.; 18 Laboratory for Biomolecular Modeling and Protein Purification and Structure Facility, EPFL and Swiss Institute of Bioinformatics, Lausanne Switzerland.; 19 Institute of Biochemistry and Cell Biology, National Research Council, Naples, Italy.; 20 Department of Biochemistry and Microbiology, University of Victoria, Victoria, Canada.; 21 Global Health Institute, School of Life Sciences and; 22 Brain Mind Institute, School of Life Sciences, EPFL, Lausanne, Switzerland.; 23 Berlin Institute of Health, Institut für Biochemie, Charité-Universitätsmedizin Berlin, Corporate Member of Freie Universität Berlin, Humboldt-Universität zu Berlin, Berlin, Germany.; 24 Radboud University Medical Center, Department of Human Genetics, Nijmegen, Netherlands.; 25 Radboud Institute for Molecular Life Sciences, Nijmegen, Netherlands.; 26 Radboud University Medical Center, Department of Pediatric Neurology, Amalia Children’s Hospital and Donders Institute for Brain, Cognition and Behavior, Nijmegen, Netherlands.; 27 Molecular Genetics of Neurodevelopment, Department of Woman and Child Health, University of Padova, Padova, Italy.; 28 Fondazione Istituto di Ricerca Pediatrica (IRP), Città della Speranza, Padova, Italy.; 29 Medical Genetics Department, APSS Trento, Trento, Italy.; 30 Center for Medical Genetics and Hunan Key Laboratory of Medical Genetics, School of Life Sciences, Central South University, Changsha, Hunan, China.; 31 Department of Neurology, Odense University Hospital, and Department of Clinical Research, University of Southern Denmark, Odense, Denmark.; 32 Department of Clinical and Molecular Genetics, University Hospital Vall d′Hebron, Medicine Genetics Group, Valle Hebron Research Institute, Barcelona, Spain.; 33 Fetal Medicine Unit and Prenatal Diagnosis Center, Shanghai First Maternity and Infant Hospital, Tongji University School of Medicine, Shanghai, China.; 34 Department of Family and Pediatric Nursing, Medical University, Wroclaw, Poland.; 35 Sección de Genética Médica, Servicio de Pediatría, Hospital Clínico Universitario Virgen de la Arrixaca, IMIB-Arrixaca, CIBERER-ISCIII, Murcia, Spain.; 36 Quantitative Genomic Medicine Laboratories, S.L., CSO & CEO, Esplugues del Llobregat, Barcelona, Catalunya, Spain.; 37 Telethon Institute of Genetics and Medicine (TIGEM), Pozzuoli, Naples, Italy.; 38 Department of Precision Medicine, University of Campania “Luigi Vanvitelli,” Naples, Italy.; 39 Department of Pediatrics, ASST Lariana Sant’ Anna Hospital, San Fermo Della Battaglia, Como, Italy.; 40 North of Scotland Regional Genetics Service, Clinical Genetics Centre, Ashgrove House, Foresterhill, Aberdeen, United Kingdom.; 41 W Midlands Clinical Genetics Service, Birmingham Women’s Hospital, Edgbaston Birmingham, United Kingdom.; 42 Northern Regional Genetics Laboratory, Newcastle upon Tyne, United Kingdom.; 43 Department of Genetics, University Medical Center Utrecht, Utrecht University, Utrecht, Netherlands.; 44 Genetica AG, Humangenetisches Labor und Beratungsstelle, Zürich, Switzerland.; 45 Centre de Référence Anomalies du Développement et Syndromes Malformatifs, FHU TRANSLAD, Hôpital d’Enfants, CHU Dijon, Dijon, France.; 46 UMR1231 GAD, INSERM – Université Bourgogne-Franche Comté, Dijon, France.; 47 Unité Fonctionnelle Innovation en Diagnostic Génomique des Maladies Rares, FHU-TRANSLAD, CHU Dijon Bourgogne, Dijon, France.; 48 Department of Radiology at Columbia University Irving Medical Center, New York, New York, USA.; 49 Department of Neurology, Columbia University Irving Medical Center, New York Presbyterian Hospital, Columbia University Medical Center, New York, New York, USA.; 50 Institute of Pharmacology and Toxicology University of Zürich, Zürich, Switzerland.; 51 Division of Clinical and Metabolic Genetics, The Hospital for Sick Children, Toronto, Ontario, Canada.; 52 Department of Medical Genetics, University of Alberta, Edmonton, Alberta, Canada.; 53 Greenwood Genetic Center and the Medical University of South Carolina, Greenwood, South Carolina, USA.; 54 Department of Pediatric Neurology, Indira Gandhi Institute of Child Health, Bangalore, India.; 55 Division of Nephrology, Department of Medicine, Columbia University, New York, New York, USA.; 56 Department of Biochemistry and Cell Biology, National Institute of Infectious Diseases, Tokyo, Japan.; 57 Department of Biochemistry and Molecular Biology, The University of British Columbia, Vancouver, British Columbia, Canada.; 58 Department of Pediatrics,; 59 Department of Neurology,; 60 Columbia Stem Cell Initiative, and; 61 Initiative for Columbia Ataxia and Tremor, Columbia University Irving Medical Center, New York, New York, USA.

**Keywords:** Cell biology, Genetics, Lipid rafts, Neurodevelopment

## Abstract

Neural differentiation, synaptic transmission, and action potential propagation depend on membrane sphingolipids, whose metabolism is tightly regulated. Mutations in the ceramide transporter CERT (*CERT1*), which is involved in sphingolipid biosynthesis, are associated with intellectual disability, but the pathogenic mechanism remains obscure. Here, we characterize 31 individuals with de novo missense variants in *CERT1*. Several variants fall into a previously uncharacterized dimeric helical domain that enables CERT homeostatic inactivation, without which sphingolipid production goes unchecked. The clinical severity reflects the degree to which CERT autoregulation is disrupted, and inhibiting CERT pharmacologically corrects morphological and motor abnormalities in a *Drosophila* model of the disease, which we call ceramide transporter (CerTra) syndrome. These findings uncover a central role for CERT autoregulation in the control of sphingolipid biosynthetic flux, provide unexpected insight into the structural organization of CERT, and suggest a possible therapeutic approach for patients with CerTra syndrome.

## Graphical abstract

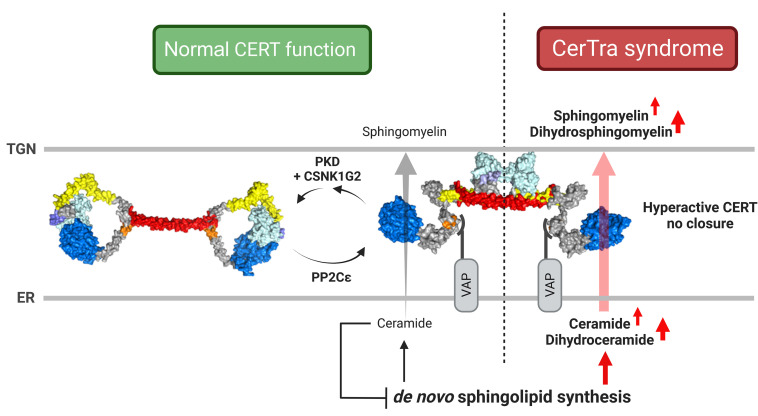

## Introduction

Sphingolipids play numerous essential roles in membrane structure, signal transduction, and brain development and function (1–3). The central nervous system is particularly affected by disturbances in sphingolipid production or clearance: defective production can cause hereditary sensory neuropathy, spastic paraplegia, or infantile epilepsy syndrome, whereas toxic accumulation of sphingolipids underlies a number of devastating inborn errors of metabolism such as Gaucher disease, Farber disease, Niemann-Pick disease type A, Krabbe, Tay-Sachs, and Sandhoff diseases (1, 4). Sphingolipid metabolic fluxes therefore must be tightly regulated through homeostatic circuits (5). A key checkpoint in sphingolipid biosynthesis occurs at contact sites between the endoplasmic reticulum (ER) and the *trans* Golgi membrane, where the ceramide transporter (CERT) transfers ceramide (Cer) from the ER to the *trans* Golgi for its conversion to sphingomyelin (SM) (6). When sufficient rates of SM production are reached, CERT is phosphorylated and undergoes a conformational change that renders it inactive (7).

Given the central position of CERT in sphingolipid metabolism, its malfunction should be detrimental to human health and particularly to neural function. Thus far, 1 case report and several case-control screening studies have described associations between variants in ceramide transporter 1 (*CERT1*), the gene that encodes CERT, and neurological abnormalities (8–16). Nonetheless, there has not been a systematic assessment of the mutational landscape of *CERT1* in humans, and whether or how *CERT1* mutations cause neurological disease remains to be proven.

In this study, we characterized 31 unrelated individuals with 22 distinct missense variants in *CERT1*, 18 of which have not to our knowledge been previously reported. These patients have a syndromic presentation characterized by infantile hypotonia; mild dysmorphologies (affecting the face, hands or feet); variable degrees of intellectual disability and motor and speech delays; increased pain tolerance; and seizures. We investigated the effect of Cer transporter (CerTra) mutations on CERT function, regulation, and structure. We found that several CerTra mutations disrupt CERT autoregulation, leading to a gain in CERT activity, increased de novo sphingolipid synthesis in the ER, and skewed metabolic flux toward the production of potentially neurotoxic compounds. We found that CERT gain of function in *Drosophila melanogaster* led to head and brain size defects and impaired locomotor activity, which we corrected by pharmacological inhibition of CERT. Biochemical characterization of disease-causing *CERT1* mutations led us to identify an unanticipated structured region within CERT that is essential to its autoregulation and to sphingolipid homeostasis.

## Results

### Characterization of CERT1-associated phenotypes.

**Figure 1:**
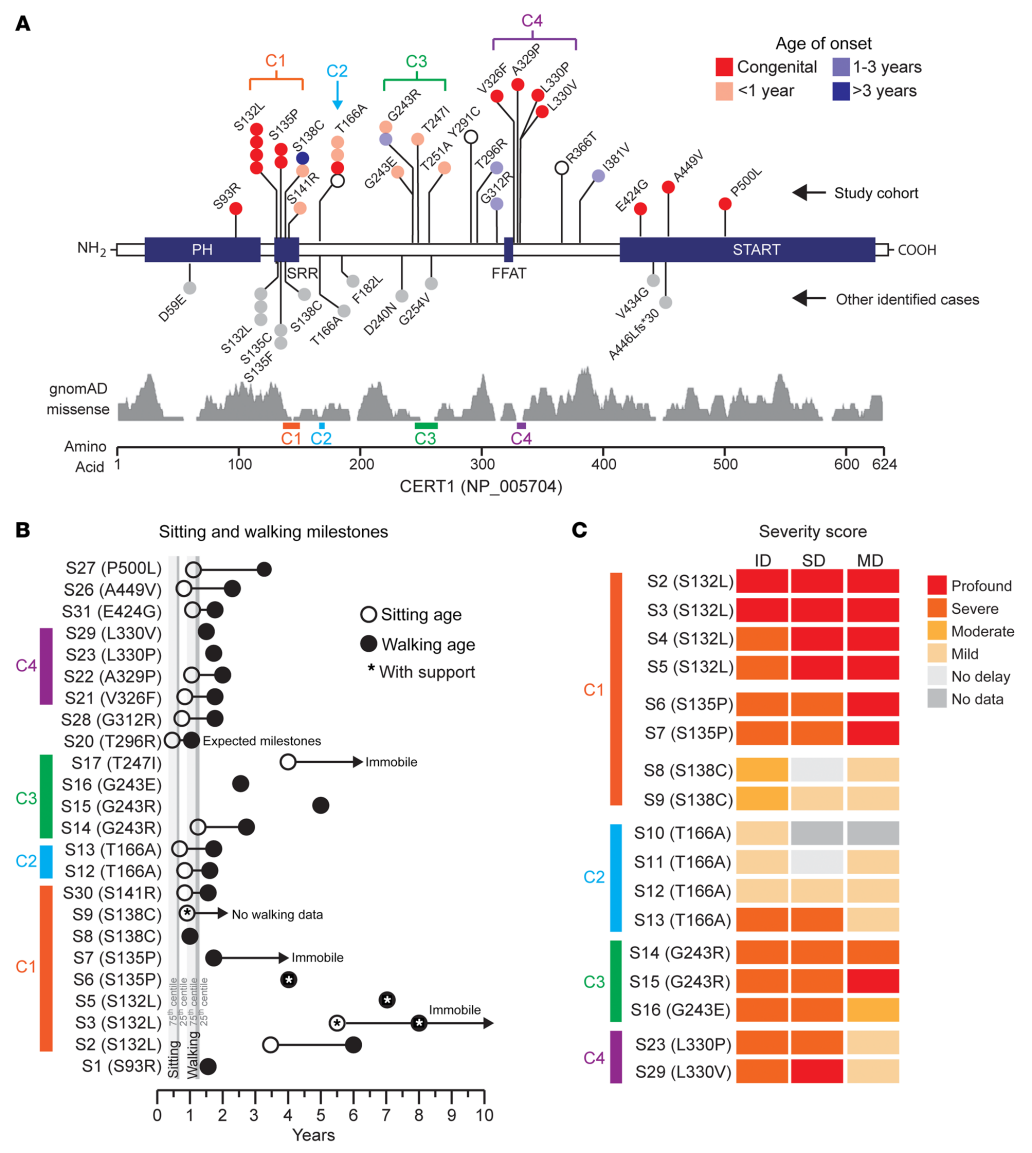
Mutations in *CERT1* lead to a neurodevelopmental syndrome. (**A**) Schematic representation of functional domains in CERT. The N-terminal PH domain interacts with phosphoinositide phosphatidylinositol-4-phosphate [PtdIns(4)*P*] (25) on the *trans* Golgi. The SRR is the target of protein kinase D (PKD) and casein kinase 1γ2 (CSNK1G2) phosphorylation. The FFAT (2 phenylalanines in an acidic tract) motif interacts with the integral membrane proteins VAP-A and VAP-B on the ER (71), and a C-terminal START-related domain extracts Cer from the ER membrane and delivers it to the *trans* Golgi (7). The schematic shows coding variants in *CERT1* (NP_005704) in our cohort of 31 individuals above the gene diagram and other individuals identified from clinical databases (DECIPHER, version 9.31, ClinVar, and VKGL) below it (Supplemental Table 1). Colors indicate the age of onset; gray indicates that no information is available. The distribution of gnomAD singleton missense variants for healthy individuals is plotted below. (**B**) Range of severity in motor delays compared with the 75th percentile (light gray) and 25th percentile (dark gray), adapted from values published by the Denver Developmental Screening Test II. White and black circles indicate delayed sitting and walking ages, respectively; asterisks indicate that the individual needs sitting or walking support. White circles with an arrow indicate individuals who are currently immobile or have not yet developed independent walking. (**C**) Heatmap shows the degree of intellectual disability (ID), speech delay (SD), and motor delay (MD) of the patients bearing frequent *CERT1* mutations. See Supplemental Table 2 for scores. C1, cluster 1; C2, cluster 2; C3, cluster 3; C4, cluster 4.

Most of the *CERT1* variants reported so far have been associated with intellectual disability (13) (Figure 1A, and Supplemental Table 1; supplemental material available online with this article; https://doi.org/10.1172/JCI165019DS1), but the clinical phenotype has been characterized for only 1 of these individuals, who carries a p.S135P variant (13). We therefore sought *CERT1* mutation carriers from multiple international disease consortia and databases (see Methods and Supplemental Methods). We identified 50 patients who carry a potentially pathogenic variant in *CERT1* (45 in the coding sequence; hg19:NM_005713.3, Figure 1A, and Supplemental Tables 1 and 2) (17). We obtained thorough clinical histories for 31 patients: 27 from the present cohort, 3 who were initially included in genetic screening consortia for intellectual disability (8, 10, 12), and the patient who had been characterized clinically (13) (Supplemental Tables 2 and 3, and Supplemental Clinical Appendix for each patient). Family segregation confirmed that *CERT1* variants occurred de novo in 93% (25 of 27) of patients. Biparental samples were not available for 4 of the patients (referred to herein as subject 1 [S1], S19, S20, and S24; see Supplemental Clinical Appendix). The p.V326F variant in S21 was inherited from her reportedly unaffected father, whereas the p.A449V variant in S26 was inherited from her mother, who was diagnosed with intellectual disability (Supplemental Table 2 and Supplemental Clinical Appendix).

The detailed clinical information we were able to gather (see Supplemental Clinical Appendix and Supplemental Table 2) revealed cognitive, motor, and speech delays of variable degrees of severity. Of the patients for whom we had birth information, only 4 were of average weight at birth; most were born slightly to significantly underweight. Similarly, only 4 of the individuals did not show some form of developmental delay by the end of the first year of life (4 of 26, 15%), with the latest onset being at age 4 years (Figure 1, A–C). Fifteen (of 24) patients had neonatal feeding difficulties, often with hypotonia or failure to thrive. These were likely early manifestations of what would later become frank motor delays, affecting 26 of 29 patients (Figure 1B). Intellectual disability ranged from mild to profound, as per the criteria of the Diagnostic and Statistical Manual of Mental Disorders, 5th Edition (DSM-5) (18) (see Methods); the latter individuals are nonverbal, lack age-appropriate daily living skills, and require safety supervision. Neurobehavioral abnormalities frequently led to a diagnosis of autism spectrum disorder (ASD) (19 of 27, 70%); some patients displayed stereotypical hand movements (14 of 18), self-injurious behavior (9 of 19), high pain tolerance (9 of 18), disrupted sleep patterns (9 of 21), attention deficit–hyperactivity disorder (10 of 19), or aggression (6 of 20). Multiple seizure types were reported (16 of 29). Neuroimaging frequently revealed a thin corpus callosum, ventriculomegaly, delayed myelination, and cerebellar atrophy (Supplemental Figure 1B).

Subtle facial dysmorphisms included anteverted nares with a depressed or broad nasal bridge, enlarged earlobes, synophrys, micrognathia, dental anomalies (protruding incisors and diastema), and palatine ridges (Supplemental Figure 1A and Supplemental Figure 2, A–E). Anomalies affecting the hands, feet, or digits included third/fourth finger syndactyly, club foot, or hallux varus (sandal gaps); the first metatarsal also tended to be short, whereas the fifth fingers tended to be long (Supplemental Figure 2F).

The majority (27 of 31, 87%) of variants from our enrolled patients occurred in the region between the pleckstrin homology (PH) and C-terminal START-related (START) domain (Figure 1A), as was the case for previously reported variants (13). These variants populated 4 distinct subregions, whereby the geometric mean distance (δg) between variants within each spatial group indicated a greater likelihood of clustering compared with random permutations (Figure 1A). Human *CERT1* produces at least 2 splicing variants that are both widely expressed (13). In this report, we have used *CERT1* isoform 1 (also known as CERT__L__) for both aa numbering, overexpression, and structural experiments.

The first 4 serine residues of the serine-rich region (SRR) (132-SMVSLVSGASGYSATSTSS-150) are hotspots for mutations (cluster 1): p.S132 (*n* = 7; 4 enrolled), p.S135 (*n* = 4; 2 enrolled), p.S138 (*n* = 4; 2 enrolled), p.S141 (*n* = 1; 1 enrolled) (Figure 1A). An alanine substitution at p.T166 was found in 5 study participants in our cohort (4 enrolled; cluster 2). Between residues 240 and 254 (cluster 3), missense variants were found at p.D240 (*n* = 1), p.G243 (*n* = 4; 4 enrolled), p.T247 (*n* = 1; 1 enrolled), and p.T251 (*n* = 1; 1 enrolled). Four variants, p.V326F, p.A329P, p.L330V, and p.L330P, are located at the C-terminal end of the CERT FFAT motif (cluster 4).

For the variants that recurred in multiple individuals, we were able to sketch out broad genotype-phenotype correlations. The most severe phenotypes — with congenital or perinatal onset, profound-to-severe intellectual disability, and the greatest motor delay — resulted from mutations at p.S132 and p.S135 (Figure 1, B and C, and Table 1). Individuals bearing mutations at p.S138, p.T166, or p.G243 tended to not have perinatal difficulties and achieved early developmental milestones (S8 did not show difficulties until 4 years of age) but then regressed or slowed in their development. Seizures probably contribute to the developmental delays: neither individual with a p.S138C mutation had seizures and have only moderate intellectual disability, but S13, the most severely affected of the p.T166A carriers, has an epileptic encephalopathy that apparently halted her development at 19 months of age. All variants associated with more severe phenotypes were predicted to be deleterious by Combined Annotation-Dependent Depletion, version 1.6 (CADD v1.6), Rare Exome Variant Ensemble Learner (REVEL), Mendelian Clinically Applicable Pathogenicity (M-CAP), and Eigen (Supplemental Table 4). Approximately half of the variants among the more moderately affected individuals had inconsistent pathogenicity predictions across algorithms. On average, variants among patients had significantly greater CADD v1.6, M-CAP, REVEL, and Eigen pathogenicity scores than did Genome Aggregation Database (gnomAD) singleton missense variants (*P* < 0.001, Mann-Whitney *U* test).

Variants p.D59E (15), p.T166A (19), and p.F182L (9) were previously identified in ASD cohorts (Supplemental Table 1). To determine the extent to which *CERT1* mutations contribute to autism, we conducted a targeted de novo analysis interrogating the Simons Foundation Powering Autism Research (SPARK) initiative database, which includes genomic data from nearly 35,000 individuals with ASD (See Supplemental Methods). No de novo protein-altering variants were observed in the Simons exome cohort. Denovo-db (version 1.6.1) confirmed the negative findings for the exome cohort but identified 1 de novo genome missense mutation (pos=74721285, T>C, c.880A>G, p.(T294A), exon6) in 1 of 516 trios. This analysis, along with the fact that none of the individuals in our cohort was diagnosed with ASD as a primary condition, indicates that *CERT1* variants are unlikely to be a significant contributor to autism but instead cause a recognizable neurodevelopmental syndrome distinct from ASD, which we will refer to as CerTra syndrome.

### CerTra mutations disrupt CERT regulation.

CERT oscillates between cytosolic and membrane-associated forms (20), according to its activation state in the homeostatic cycle (7). Active, membrane-associated CERT provides Cer to SM synthase 1 (SMS1) for its conversion to SM, with concomitant production of diacylglycerol. The diacylglycerol produced in the SM synthase 1 reaction triggers a signaling response involving the recruitment of protein kinase D (PKD) to the *trans* Golgi (21–23). PKD initiates CERT phosphorylation at p.S132 (7); and CSNK1G2 (casein kinase 1 γ 1) then phosphorylates the rest of the SRR phosphosites (24–27). Following phosphorylation, CERT undergoes a conformational change that leads to its cytosolic redistribution and inactivation (20, 26, 28); all this can then be reversed by the action of the ER phosphatase PP2Cε (protein phosphatase 2C epsilon) (29). Mutations leading to abnormally activated CERT redistribute the protein to Golgi membranes and cytosolic spots, while inactivating mutations redistribute it to the cytosol (30, 31).

**Figure 2:**
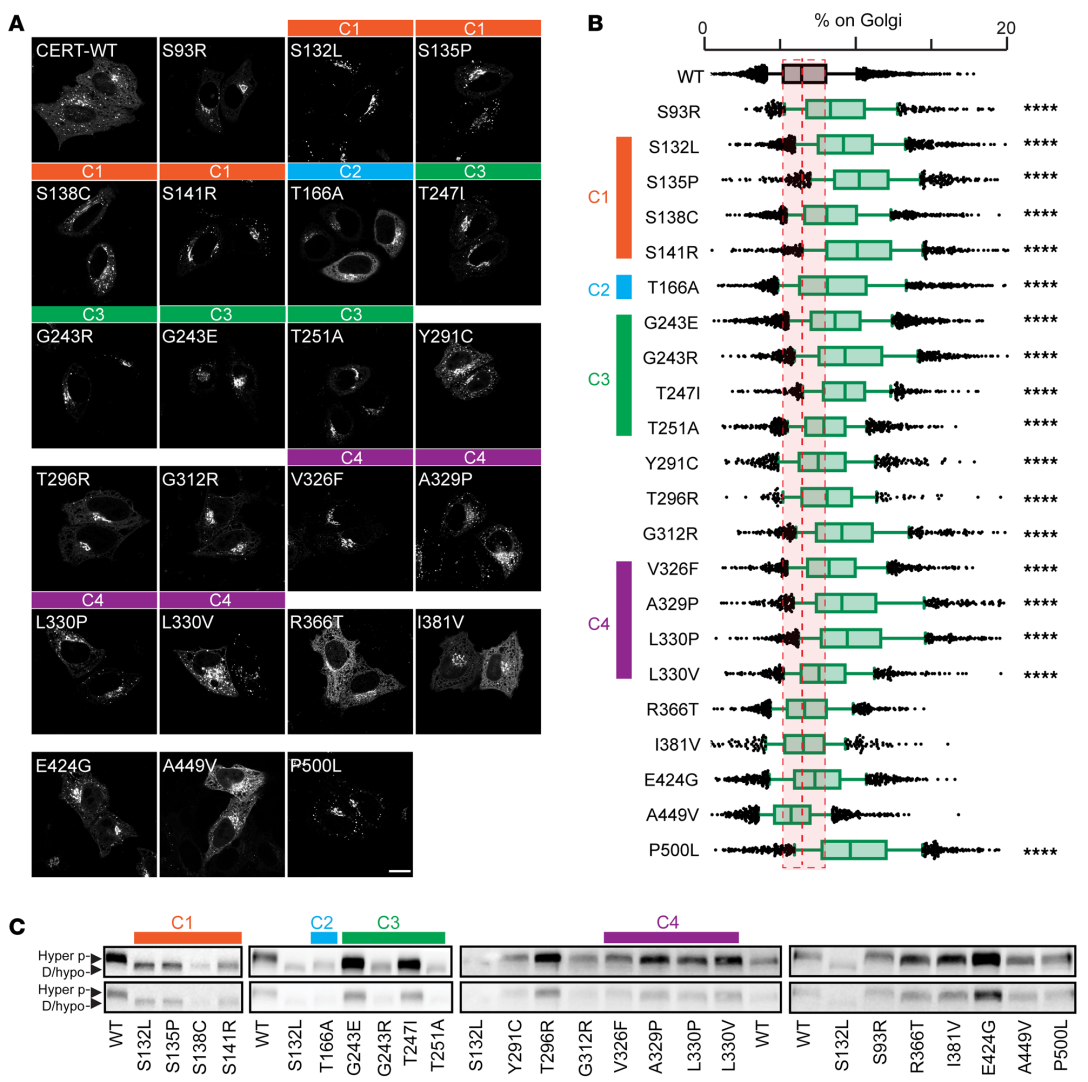
Disease-causing variants result in CERT misregulation. (**A**) CERT-GFP WT and mutant localization in HeLa cells analyzed by confocal microscopy. Scale bar: 20 μm. (**B**) Percentage of CERT-GFP WT and mutants associated with the Golgi complex in HeLa cells. Cells were stained with Hoechst and anti-GM130 antibody and analyzed by automated fluorescence microscopy (*n* >1,000 cells per condition; ****P < 0.0001, 1-way ANOVA, effect size >15%). WT CERT is shown in gray, and CerTra mutants are shown in green. Data are shown as box-and-whisker plots. Bars represent the median value of each data set. (**C**) Western blot of HeLa cells expressing CERT-GFP WT or mutants (*n* = 3). Hyperphosphorylated (Hyper p-) and de-/hypophosphorylated (D/hypo-) bands are indicated by arrowheads. The clusters, as in Figure 1, are indicated throughout **A**–**C**.

To determine whether disease-causing variants alter CERT localization, we used automated microscopy to capture GFP-tagged WT and CerTra-associated CERT variants in transfected HeLa cells (see Supplemental Methods). Eighteen of the 22 CERT mutants exhibited a greater tendency than WT to associate with the perinuclear membranes and cytosolic spots (Figure 2, A and B). These included all variants mapping to clusters 1 to 4 (Figure 1A), those located between clusters 3 and 4, the p.S93R in the PH domain, and the p.500L in the START domain. We observed a similar phenotype in patient-derived fibroblasts bearing the p.G243R mutation (Supplemental Figure 3, A–C). Four consecutive variants (p.R366T, p.I382V, p.E424G, p.A449V) did not show a different distribution compared with CERT WT.

We next evaluated the phosphorylation pattern of CERT WT and mutants by SDS-PAGE (Figure 2C). WT CERT migrates on SDS gels as a doublet, with the fast- and slow-migrating bands corresponding to the active/hypophosphorylated and the inactive/hyperphosphorylated forms of CERT, respectively (24). By contrast, all variants belonging to clusters 1, 2, and 3 showed hypophosphorylation, whereas mutations outside of these clusters did not. Mass spectrometry–based assessment of selected variants belonging to clusters 1, 2, and 3 showed that these *CERT1* mutations did not affect CERT phosphorylation outside the SRR or SRR monophosphorylation (likely on p.S132), and suggests that CSNK1G2-mediated SSR phosphorylation is defective in these mutants (Supplemental Figure 3D).

**Figure 3:**
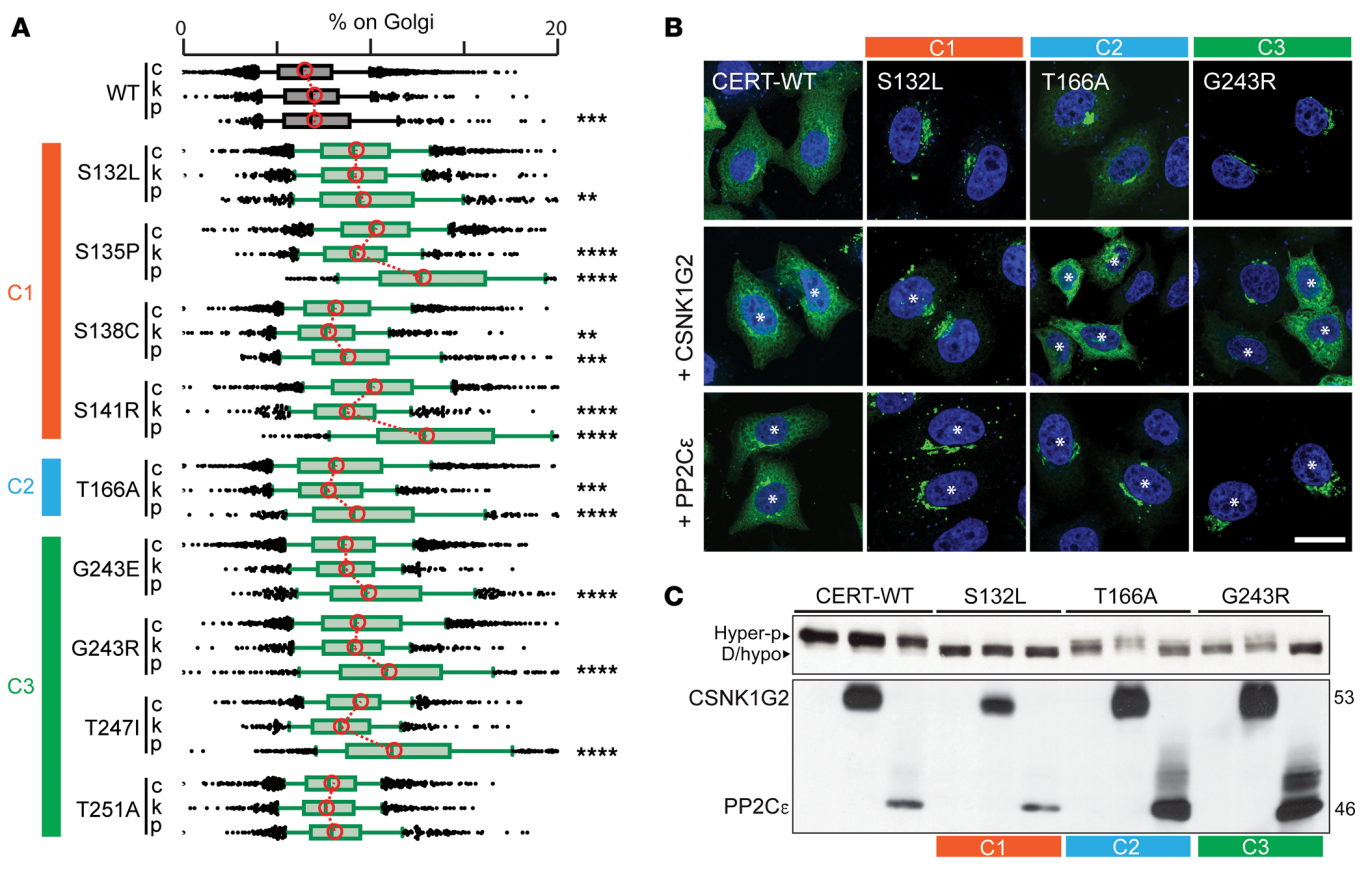
Disease-causing CERT variants are susceptible to phosphoregulation. (**A**) Percentage of CERT-GFP WT and mutants from clusters 1, 2, and 3 associated with the Golgi complex in HeLa cells overexpressing CSNK1G2-HA (k) or PP2Cε-HA (p). Cells were stained with Hoechst, anti-GM130 antibody, and anti-HA antibody and analyzed by automated fluorescence microscopy (*n* >500 cells per condition; **P < 0.01, ***P < 0.001, and ****P < 0.0001, 1-way ANOVA). Bars represent the median value of each data set. (**B**) Subcellular localization of CERT-GFP WT, p.S132L, p.T166A, and p.G243R in HeLa cells expressing PP2Cε-HA or CSNK1G-HA. Cells were stained with DAPI (blue) and anti-HA antibody and analyzed by confocal microscopy. Asterisks indicate cotransfected cells. Scale bar: 20 μm. (**C**) Western blot analysis of HeLa cells coexpressing CERT-GFP WT or mutants with CSNK1G2-HA or PP2Cε-HA (*n* = 3). C, cluster (as represented in Figure 1).

Although we expected hypophosphorylation and increased membrane recruitment for CERT mutants within the SRR (cluster 1) (13, 27), this was not intuitive for clusters 2 and 3. We therefore investigated the regulation of the 2 most frequent *CERT1* mutations in these clusters, p.G243R, for which some biochemical characterization was already reported (30), and p.T166A. These 2 variants were effectively phosphorylated at p.S132 by PKD in both in vitro and cell-based assays (Supplemental Figure 3, D and E). Moreover, the p.S132 phosphomimetic mutant (HGS 130-132 DDD) (27) in the p.G243R and p.T166A backgrounds did not differ in either localization or SDS-PAGE migration (Supplemental Figure 3, F–H). This supports the idea that the cluster 2 and 3 mutations affect CERT regulation downstream of PKD phosphorylation. To test this possibility, we coexpressed CSNK1G2 or PP2Cε with the hypophosphorylated CERT mutants. Overexpression of CSNK1G2 led to reduced membrane association of most mutants (p.S132L served as a negative control) (Figure 3, A–C), indicating that these can be phosphorylated and inactivated by CSNK1G2. Interestingly, although PP2Cε overexpression had little effect on WT CERT phosphorylation or localization, consistent with previous results (27), hypophosphorylated CERT mutants were sensitive to PP2Cε overexpression (Figure 3, A–C).

Thus, CerTra-associated *CERT1* mutations generally disturb CERT regulation and promote its membrane association. Mutations in clusters 1, 2, and 3 also reduce the pool of hyperphosphorylated/inactive CERT by influencing the CERT phosphorylation cycle that depends on the consecutive action of PKD and CSNK1G2 kinases and on the PP2Cε phosphatase.

### CerTra mutations disrupt sphingolipid homeostasis.

**Figure 4:**
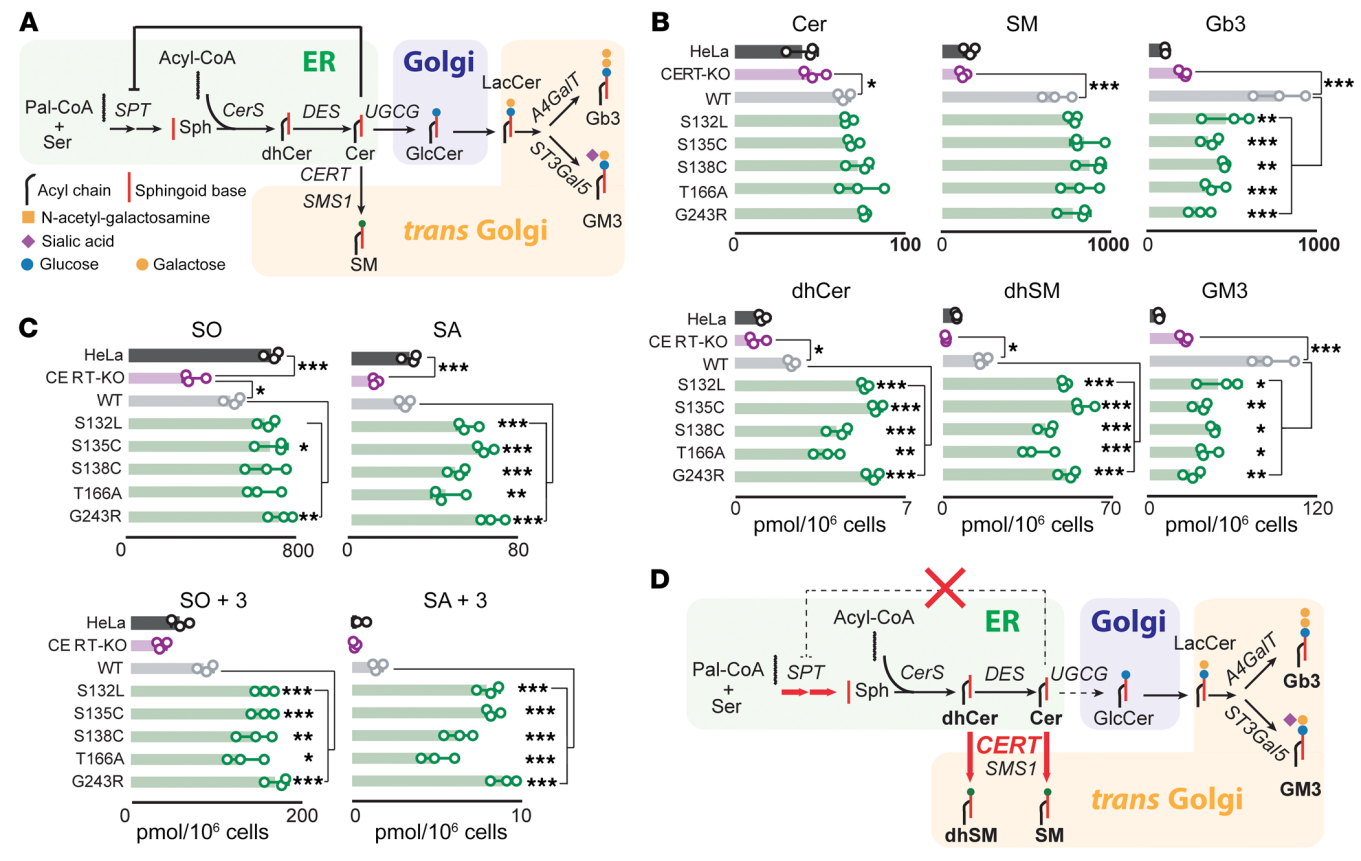
Several *CERT1* mutations increase sphingolipid levels. (**A**) Schematic representation of the de novo sphingolipid biosynthetic pathway with the main enzymes involved (shading indicates the prevalent intracellular localization of synthetic reactions). (**B**) Mass spectrometry profile of sphingolipids in HeLa *CERT1*-KO cells overexpressing selected *CERT1* mutants from clusters 1, 2, and 3. Values are total levels across the major fatty acid chain lengths of the indicated sphingolipids. The levels of individual species are reported in Supplemental Figure 4A. *n* = 3. Data are the mean ± SD. *P < 0.05, **P < 0.01, and ***P < 0.001, by 1-way ANOVA. (**C**) Effect on the LCB of CERT mutations in HeLa cells. LCB profiles were evaluated by incorporation of an isotope labeled (2,3,3-d^3^,^15^*N*)-l-serine. *n* = 3. Data are the mean ± SD. *P < 0.05, **P < 0.01, and ***P < 0.001, by 1-way ANOVA. (**D**) The de novo sphingolipid biosynthetic pathway as modified by *CERT1* mutations in clusters 1, 2, and 3. CerS, ceramide synthases; DES, dihydroceramide desaturase; SMS1, sphingomyelin synthase 1; UGCG, glucosylceramide synthase; ST3Gal5, GM3 synthase; A4GalT, Gb3 synthase; Sph, sphingolipid.

Sphingolipid biosynthesis starts at the ER with the generation of Cer (32–34). Cer is then transported to the Golgi complex by vesicular carriers for the production of glucosylceramide and downstream glycosphingolipids, or transferred directly to the *trans* Golgi by CERT for its conversion to SM (6) (Figure 4A).

We profiled sphingolipids in *CERT1*-deficient (*CERT1*-KO) HeLa cells expressing 6 CERT mutants tagged with GFP. *CERT1* KO causes a defect in Cer-to-SM conversion (35) that is expected to be reverted by the expression of active CERT. Overexpression of WT CERT in *CERT1*-KO cells, in fact, induced a 5-fold increase in SM levels coupled with an unexpected increase in Cer and complex glycosphingolipids (Figure 4B and Supplemental Figure 4A). Overexpression of CERT mutants also increased SM and Cer levels but markedly reduced glycosphingolipid levels compared with WT levels. Unexpectedly, CERT overexpression increased dihydroceramide (dhCer) and dihydrosphingomyelin (dhSM), and this was even more pronounced with mutant overexpression (Figure 4B and Supplemental Figure 4A). Variants associated with more severe manifestations in patients (i.e., at p.S132, p.S135, and p.G243) were associated with the greatest increases.

We observed that *CERT1* overexpression produced an overall increase in sphingolipid levels (Figure 4B). To determine whether this was due to increased synthesis, decreased catabolism, or both, we used a D_3_-^15^*N*-serine labeling approach (see Methods). Lipids were extracted and subjected to chemical hydrolysis to release long-chain bases (LCBs) from sphingolipids and dihydrosphingolipids. These LCBs reflect total levels of steady-state and newly synthesized sphingolipids, represented by sphingosine (SO) and sphingosine+3 (SO+3), respectively, while steady-state and newly synthesized dihydrosphingolipids are represented by sphinganine (SA) and sphinganine+3 (SA+3), respectively. Overexpression of CERT mutants had little effect on SO but increased SO+3 by 200%. All mutants had twice the amount of SA compared with WT CERT, but SA+3 levels rose at least 300% in the case of the mildest mutant, p.T166A, and considerably more for the other mutants (Figure 4C).

These data indicate that CERT activity promotes de novo sphingolipid production, possibly by relaxing the Cer-dependent inhibition of serine palmitoyltransferase (SPT) (36) (Supplemental Figure 5A). To test this hypothesis, we repeated our metabolic labeling experiment in parental and *CERT1*-KO HeLa cells treated with the CERT inhibitor *N*-[(1R,3S)-3-hydroxy-1-(hydroxymethyl)-3-phenylpropyl]-dodecanamide (HPA-12) (37) or, as a control, with myriocin (Myr), an inhibitor of SPT and therefore of sphingolipid production (Supplemental Figure 4A). Treatment with HPA-12 reduced sphingolipid synthesis more than 80% (similar to what was obtained by treating cells with Myr) in HeLa cells but had no effect on *CERT1*-KO cells, in which sphingolipid synthesis was already greatly reduced (Supplemental Figure 5B).

We confirmed the metabolic alterations induced by overexpression of *CERT1* mutants in *CERT1*-KO cells in patient-derived fibroblasts bearing the p.G243R mutation and lymphoblasts bearing the p.T166A mutation. Patient cells produced substantially higher amounts of dhSM and dhCer than did their WT counterparts (Supplemental Figure 5, C and E). In contrast, inhibition of CERT activity with HPA-12 markedly reduced total dhCer and dhSM in both WT and patient-derived cells.

We observed a similar profile in steady-state sphingolipid levels and new synthesis when we examined patient cells after labeling with D_3_-^15^*N*-serine (Supplemental Figure 5, D and F). SA+3 backbone levels were much higher in patient-derived cells than in controls. The increase in sphingolipid synthesis observed in patients’ cells was blocked by the administration of HPA-12, confirming that these effects are indeed caused by altered CERT activity (Supplemental Figures 4 and 5).

These data indicate that CERT activity is inherently coupled to the homeostatic regulation of de novo sphingolipid synthesis and suggest that frequent CerTra mutations cause an increase in CERT activity (Figure 4D). 

### CerTra mutations reveal an uncharacterized structural domain in CERT.

The structures of the PH and START domains of CERT have been solved independently and as a complex (20, 38, 39), but little is known about the region between these 2 domains. It has been assumed to be largely unstructured except for a predicted coiled-coil segment that mediates CERT oligomerization upon UV irradiation (40, 41). Given the appreciable effect of CERT mutations in clusters 2 and 3 (Figure 1C), we decided to explore the structural context of these clusters. To this end, we subjected recombinant CERT to hydrogen-deuterium (H/D) exchange mass spectrometry (HDX-MS), which allows the study of protein dynamics and folding by assessing the solvent accessibility of protein surfaces (42, 43).

**Figure 5:**
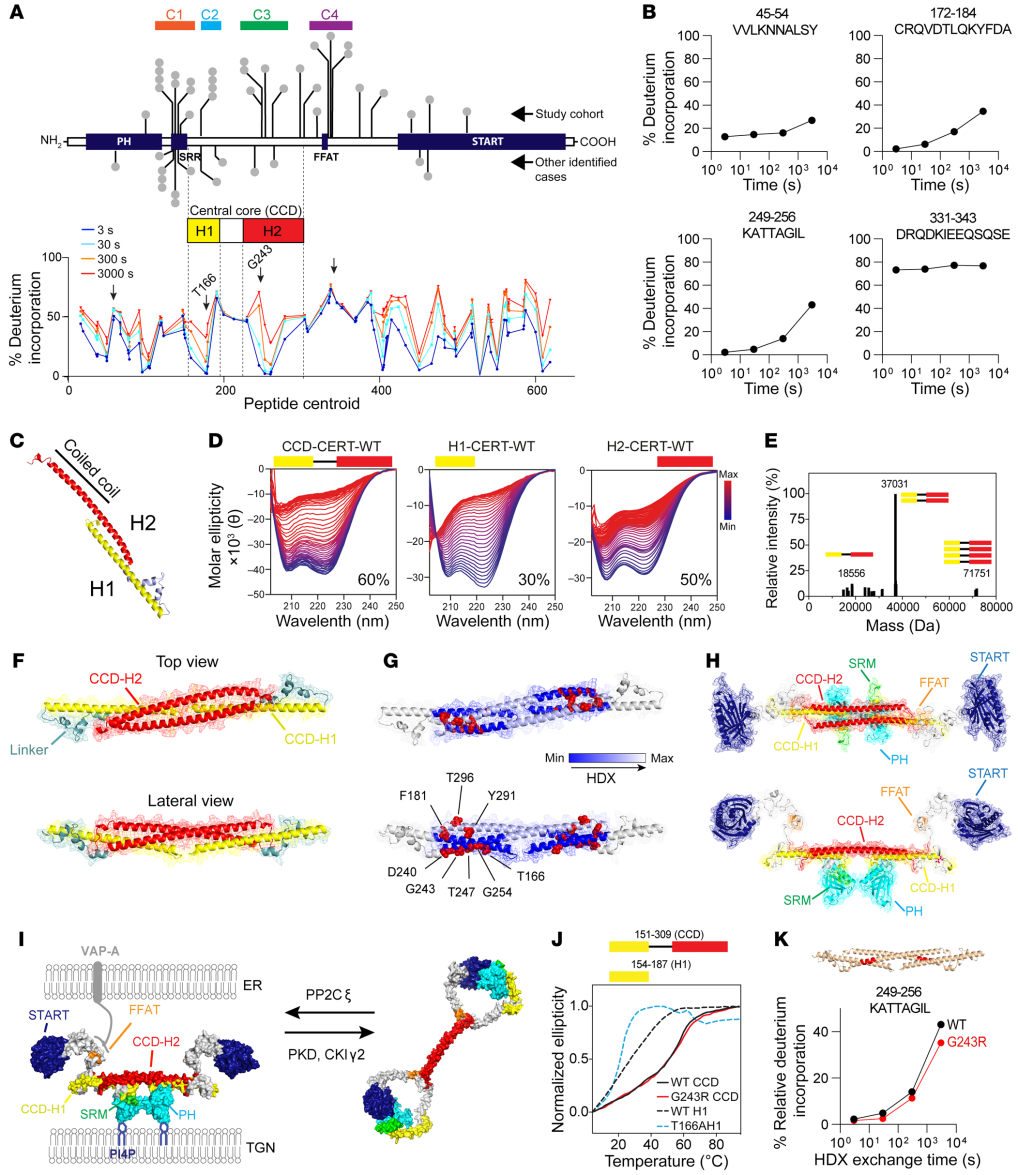
Disease-causing *CERT1* mutations affect the central core structure of CERT. (**A**) Domain organization of CERT with its CCD and the predicted H1 and H2 helices. The global percentage of HDX is shown for all peptides graphed according to their central residue number. The mean of 3 experiments is shown. (**B**) Deuterium incorporation over time of 4 selected peptides (highlighted with arrows on the HDX profile). Data are from Supplemental Tables 5 and 6. (**C**) Molecular model of CERT’s CCD based on contact prediction. Helix H1 is shown in yellow and H2 in red. (**D**) Thermostability of intervening regions showing a difference in HDX by circular dichroism: the samples were heated from 4°C to 94°C; the percentage indicates the helicity of each construct at 20°C. (**E**) Deconvoluted mass spectrum of purified recombinant CERT 151-309 WT. The value 18.5 kDa represents the molecular weight of the monomer, 37 kDa a dimer, and 71.5 kDa a tetramer. (**F**) Molecular model of CERT 151-309 WT as an antiparallel dimer. (**G**) Model showing the location of aa mutated in CerTra syndrome. The areas differentially exposed to deuterium exchange are indicated according to the color scale. (**H**) Molecular model of CERT WT as an antiparallel dimer. (**I**) Molecular model of CERT WT at the ER-TGN membrane contact site in its active and inactive conformations. (**J**) Thermostability of CERT 154-187 WT and p.T166A and CERT 151-309 WT and p.G243R by circular dichroism. (**K**) Peptide in the CCD displaying decreases in exchange in the p.G243R mutant compared with WT. These changes are mapped on the hypothetical CCD structure.

As expected, the HDX-MS profile of CERT highlighted the presence of amides protected from H/D exchange at early time points of exchange at the N- and C-terminal regions, corresponding to the PH (aa 23–117) and START (aa 389–618) globular domains (Figure 5, A and B). The areas from aa 187 to 218 and from aa 300 to 381 were highly accessible to solvent and therefore probably contained no or a very dynamic secondary structure. Strikingly, regions encompassing cluster 2 and 3 mutations (i.e., aa 152–187 and aa 240–300) were heavily protected from exchange, indicating secondary structure formation and/or their engagement in protein-protein interactions (i.e., CERT oligomerization).

Structural modeling based on trRosetta (44) and AlphaFold (45) predicted the existence of 2 helices encompassing residues 154–187 (H1) and 242–304 (H2) (Figure 5C). Coiled-coil predictors (46) also suggested the existence of a coiled-coil region spanning part of H2 (aa 263–303) (Supplemental Figure 6A). Circular dichroism confirmed that purified CERT 151–309, hereafter called the CERT central core domain (CCD) in reference to a structurally analogous region characterized in the lipid transfer protein OSBP (47), and the synthetic peptides of H1 and H2 were indeed helical; the CCD had 60% helical content and a melting temperature of 43°C (Figure 5D).

Residue coevolution calculations using direct coupling analysis (44) on an ad hoc alignment supported a coiled-coil arrangement of H1 and H2, but the calculations also proposed pairs of contacting residues that could not be satisfied in the monomeric model (Supplemental Figure 6B). Interestingly, native MS (48) of the CERT CCD indicated that dimers predominate (theoretical MW of 18.5 kDa, observed MW of 37 kDa) (Figure 5E). When we evaluated the stoichiometry of recombinant full-length CERT with size-exclusion chromatography coupled to multiangle light scattering (SEC-MALS) (49), we found the absolute molar mass of the full-length CERT molecule to be, on average, 142.7 kDa, which, again, is compatible with a dimeric form (Supplemental Figure 6, C–F).

We therefore asked whether CERT dimerizes through its CCD domain. SEC-MALS analysis of the START domain alone (aa 389–618) indicated that it is a monomer, an oligomeric state also shared by the PH domain alone (26). By contrast, the construct encompassing the PH plus CCD domains (aa 1–341) was dimeric. Thus, although a recent study reported that CERT is organized as a homotrimer or a higher oligomer (50), our data strongly suggest that CERT dimerizes through its CCD (Supplemental Figure 6, D–F).

We then modeled the CCD dimer by rigid docking (see Supplemental Methods). Of the 6 best dimer models obtained, 5 had an antiparallel orientation (Supplemental Figure 6G, Supplemental Data Sets 1–7). Antiparallel orientation was also supported by coevolution restraints unexplained by the 3D model of the monomer (Figure 5F). However, not all predicted contacts are explained by a unique conformation, perhaps because the dimer is highly dynamic. Nonetheless, when CERT mutations were mapped onto the highest-scoring dimer model, they populated the interface between the H1 and H2 helices of the dimeric CCD, a region particularly protected from solvent as assessed by HDX-MS (Figure 5G). Here, residues p.D240, p.G243, p.T247, p.T251, and p.G254 define the side of the H2 helix that interfaces with H1 in the segment between p.T166 and p.F182, whereas p.Y291 and p.T296 form the H2 helix in the other CCD monomer contacting the same H1 portion with a different orientation (Figure 5G).On the basis of this new evidence, in addition to the existing x-ray structures and the CCD model, we propose the following 3D organization of the full-length CERT dimer: the dimer adopts a T-shaped conformation whereby the 2 PH domains are kept in close proximity at the extremity of the T stem, and the 2 START domains are at the extremities of the 2 T arms (Figure 5H and Supplemental File 6). Upon SRR phosphorylation, CERT undergoes a conformational change, such that the PH domain first interacts with the phosphorylated SRR (26) and then forms a complex with the START domain (20). In the context of our structural model, this conformational change implies a rearrangement of the interface between H1 and H2 of the CCD, which may be impaired by CerTra mutations that map to this region (Figure 5I).

We analyzed the effect of CERT mutations on the CCD structure and evaluated the melting curves of the synthetic peptides CERT 154–187 and purified CERT 151–309 WT and their mutant counterparts. We found that p.T166A decreased CERT H1 helicity and destabilized it, while p.G243R had negligible effects on CCD secondary structure and stability (Figure 5J and Supplemental Figure 6H). Nonetheless, when we compared the HDX profile of recombinant full-length CERT WT with p.G243R, we observed diminished solvent accessibility for the p.G243R mutant in a region encompassing aa 249–256 (Figure 5K), an H2 region coordinating with H1. This suggests that, rather than impairing CCD structure, p.G243R impairs CCD dynamics and possibly its interactions with other proteins. These results suggest a model whereby the CCD enables the conformational change that takes place upon CERT phosphorylation to deactivate the protein and signal that sufficient levels of SM production have been reached. CerTra-causing *CERT1* mutations in the CCD disturb this conformational change either by altering CCD stability (p.T166A) or CCD dynamics/interactions (p.G243R).

### CERT gain of function alters sphingolipid metabolism, brain size, and locomotion in Drosophila melanogaster.

**Figure 6:**
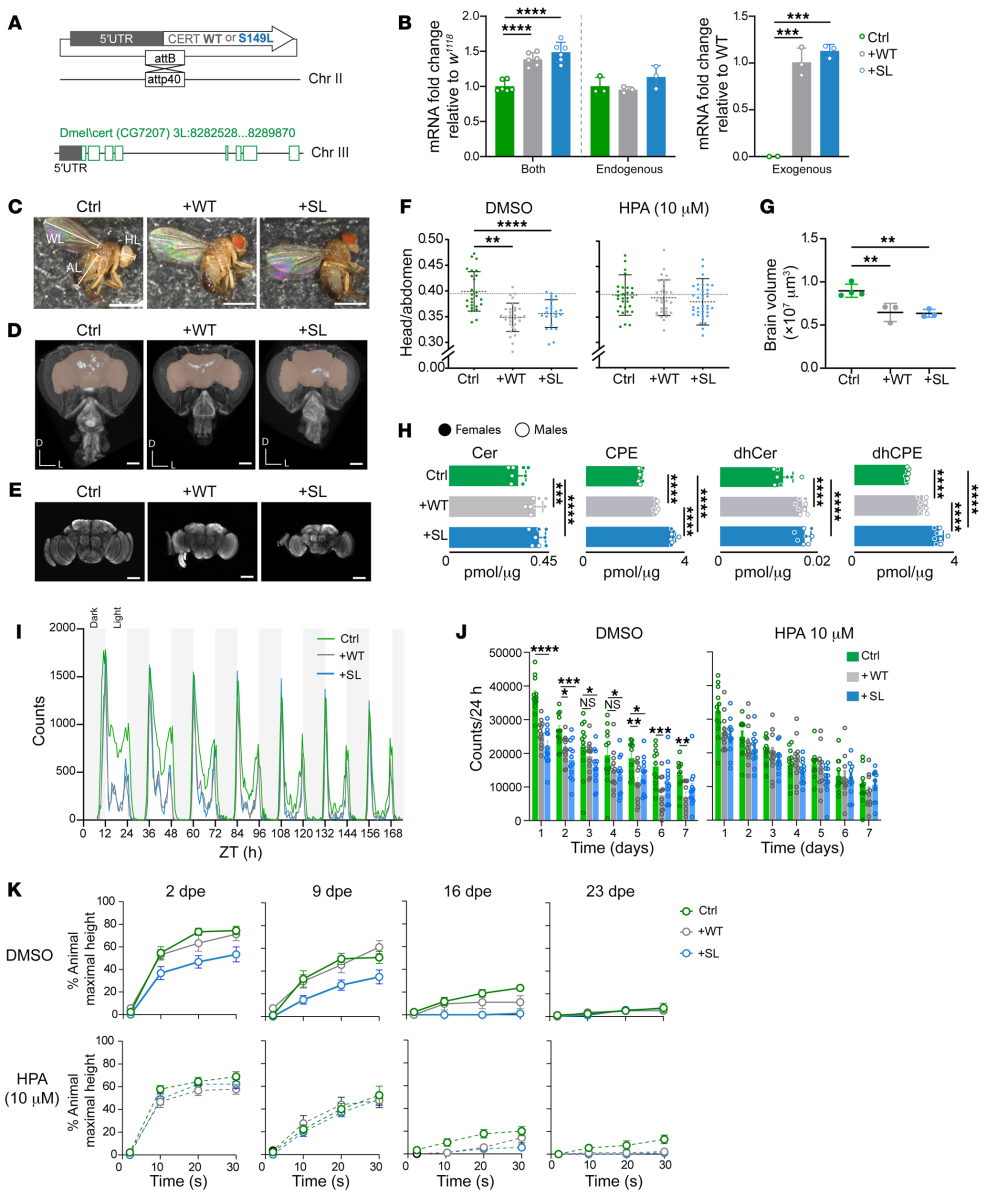
CERT gain of function causes neurological defects in *D. melanogaster*. (**A**) Schematic of transgenic *dCERT* flies on the *w^1118^* background. *dCERT^WT^*, +WT; *dCERT^p.S149L^*, +SL; Chr, chromosome. (**B**) Quantification of endogenous or exogenous *dCERT* mRNA levels in flies on the *w^1118^* background (Ctrl) in *dCERT*-transgenic flies. Data indicate the log_2_ fold change over Ctrl or +WT (*n* = 6; data are the mean ± SD). (**C**) Representative specimens of Ctrl, +WT, and +SL adult flies. Head length (HL), abdomen length (AL), and wing length (WL) were measured. Scale bars: 1 mm. (**D**) 3D rendering of a micro-CT scan of the heads from Ctrl, +WT, and +SL adults (frontal view). Brain volume is highlighted in pink. Body axes are dorsal (**D**) and left (L). Scale bars: 100 μm. (**E**) *Z*-projections of confocal stacks of whole-mount adult Ctrl, +WT, and +SL adult brains (frontal view) immunostained with anti-nc82. Scale bars: 100 μm. (**F**) HL/AL ratio of flies reared on DMSO or 10 μM HPA-12 (HPA) (Ctrl, *n* = 26 or 30, +WT *n* = 31 or 31, and +SL, *n* = 25 or 38). (**G**) Brain volume for Ctrl, +WT, and +SL flies as determined by confocal microscopy (*n* = 3–4; data are the mean ± SEM). (**H**) Mass spectrometry profile of sphingolipids in Ctrl, +WT, and +SL adult heads (*n* = 8). (**I**) Locomotion of Ctrl, +WT, and +SL flies plotted as total counts per 30 minutes over time (*n* = 16). (**J**) Locomotion of flies pretreated with 0 or 10 μM HPA-12 (*n* = 12). (**K**) Climbing ability of flies at 2, 9, 16, and 23 days post eclosion (dpe) after vigorous mechanical stress (*n* = 9). Data shown are the mean ± SEM. *P < 0.05, **P < 0.01, ***P < 0.001, and ****P < 0.0001, by 1-way ANOVA.

Altogether, our data indicate that CerTra mutations disrupt CERT autoregulation, resulting in an inappropriate gain of CERT function and excessive sphingolipid production. To test whether this is sufficient to induce neurological manifestations in an animal model, we increased the *CERT1* dosage in *D*. *melanogaster*. The metabolic activity of *D*. *melanogaster* CERT (dCERT) is similar to that of mammalian CERT (51) and shares 43% sequence identity with human CERT, including all 6 of the most recurrent CerTra variants. We generated *D*. *melanogaster* strains on the *w^1118^* background (hereafter referred to as control [Ctrl]) bearing 2 extra copies of either *dCERT^WT^* (hereafter referred to as +WT) or *dCERT^p.S149L^* (hereafter referred to as +SL; equivalent to the most common and severe CerTra mutation, p.S132L) under the control of its endogenous promoter (Figure 6A; see also Supplemental Methods).

Total *dCERT* mRNA levels were 50% higher than those in Ctrl flies in both +WT and +SL strains, with the increase being completely ascribable to the extra exogenous copies of *dCERT* (Figure 6B). The increased gene dosage did not shorten the lifespan of transgenic *Drosophila*; in fact, both the male and female animals lived slightly longer (Supplemental Figure 7A). The transgenic strains had wing and abdomen sizes comparable to those of Ctrl flies (Supplemental Figure 7B), but smaller heads and brains (Figure 6, C–G, and Supplemental Figure 7, C and D). Feeding HPA-12 to +WT and +SL larvae (see Supplemental Methods) restored head size, suggesting that increased *dCERT* activity was responsible for this phenotype (Figure 6F). The levels of Cer and dhCer were significantly and similarly increased in both +WT and +SL strains. Cer-phosphoethanolamine (CPE, the *Drosophila* equivalent of mammalian SM) (52) and dihydroceraminde (dhCPE) levels were also increased in both strains, but more substantially in the +SL strain (Figure 6H and Supplemental Figure 7, E–G). These changes were similar to those observed in whole *Drosophila* bodies (Supplemental Figure 7E) and resembled those we observed in mammalian cells (Figure 2B), suggesting that *dCERT* gain of function affects sphingolipid metabolism in flies in a manner similar to the effects of CerTra mutations in humans. It is worth noting that glycosphingolipid levels were not significantly altered in +WT or +SL strains, suggesting that changes in Cer, CPE, and their dihydro counterparts were sufficient to induce neurological phenotypes in these strains (Supplemental Figure 7E).

As most patients with CerTra experience motor delay, we monitored the locomotor activity of the +WT and +SL flies (53). Both +WT and +SL flies showed locomotor hypoactivity compared with Ctrl flies (Figure 6I). Pretreatment with HPA-12 (10 μM) for 7 days, which reduced sphingolipid levels (Supplemental Figure 7H), rescued this phenotype in the transgenic lines but had little effect on Ctrl flies (Figure 6J). Moreover, HPA-12 treatment had no effect on the locomotion of a *w^1118^* derivative *Drosophila* strain bearing a large chromosomal defect in which the *dCERT* gene was deleted (hereafter referred to as KO) (Supplemental Figure 7I), suggesting that the HPA-12 effect on locomotion requires *dCERT* expression.

Although the +WT and +SL strains share motor hypoactivity, they do have distinct metabolic phenotypes (Figure 6H), which we hypothesized should produce some difference in phenotype. Because patients with CerTra frequently have seizures, we decided to look for seizure-like reactions, such as the paralysis that seizure-susceptible flies often experience after vigorous mechanical stimulation (54, 55). Our strains did not exhibit paralysis upon mechanical stimulation, but the +SL flies showed impaired negative geotaxis that could be corrected by HPA-12 treatment (Figure 6K). Notably, negative geotaxis in nonstressed animals was indistinguishable across genotypes (Supplemental Figure 7J).

These data indicate that an increase in *dCERT* activity in *Drosophila* induced sphingolipid metabolic changes similar to those observed in CerTra syndrome, impaired locomotion, and caused central nervous system vulnerability to mechanical shock in the most severely affected line.

## Discussion

In this study, we report that multiple de novo heterozygous variants in *CERT1* caused an autosomal dominant developmental syndrome we named CerTra, which is characterized by various degrees of developmental delay, motor delay, cognitive impairment, behavioral abnormalities, and seizures. Several CerTra mutations reduce or abolish the capability of CERT to be inactivated in response to excessive SM production. This leads to uncontrolled Cer transfer out of the ER, with several consequences: (a) reduced Cer at the ER relaxes the homeostatic inhibition of SPT (56, 57), resulting in increased de novo sphingolipid synthesis; (b) reduced Cer at the Golgi, the site for glucosylceramide synthesis, impairs glycosphingolipid production; and (c) excessive CERT activity likely competes with Cer desaturase activity at the ER, leading to the transfer of a significant amount of dhCer to the *trans* Golgi and production of dhSM. Elevated dihydrosphingolipid levels cause neuropathology. For example, biallelic loss-of-function mutations in *DEGS1*, which catalyzes the final conversion of dhCer to Cer (Figure 2A), result in increased dihydrosphingolipid levels, causing a multisystem neurological disorder of both the central and peripheral nervous systems, characterized by hypomyelination and leukodystrophy (MIM #618404) (58, 59). Recessively inherited loss-of-function mutations in alkaline ceramidase 3 (*ACER3*) also result in increased dihydrosphingolipid formation and a leukodystrophy phenotype (MIM #617762) (60). Impaired glycosphingolipid synthesis is a further cause of neuropathology: loss-of-function mutations in *ST3GAL5* or *B4GALNT1*, which encode 2 key enzymes in ganglio-series glycosphingolipid synthesis (i.e., GM3 and GM2 synthases), cause neurodevelopmental regression (MIM #609056) and spastic paraplegia (MIM #609195), respectively (61–66). Recently, a specific group of mutations in SPT subunits were reported to cause childhood-onset amyotrophic lateral sclerosis (67–69) and hereditary spastic paraplegia (70). All identified mutations disturbed the homeostatic control of de novo sphingolipid synthesis, resulting in greatly elevated sphingolipid levels.

Several CerTra mutations cause lipid metabolic derangements similar to those associated with the above-listed conditions, nonetheless, the molecular mechanisms of CERT dysregulation appear to be varied. Mutations in the SRR directly impair CERT-inactivating phosphorylation, whereas mutations in the CCD hinder the conformational change that follows this event. We have not directly addressed the effects of other mutations close to the FFAT motif or in the PH and START domains. These mutations likely affect CERT interaction with VAP proteins (71) and the final events of CERT inactivation that involve intramolecular interactions between the PH and START domains (24). Four consecutive variants (p.R366T, p.I381V, p.E424G, and p.A449V) do not affect CERT localization or the phosphorylation state. When analyzed in greater depth, these variants present some doubts about their actual pathogenicity: (a) each of them is associated with a single patient, and they are not part of a recognizable cluster of mutations; (b) p.I381V has been found in 1 healthy individual; (c) p.A449V has been found in an individual whose mother and father presented with intellectual disability independently of the mutational state of *CERT1*; (d) p.R366T has been found in an individual for whom the very little information we have would point toward a very mild phenotype. Further studies will be required to assess the actual role of these variants and, more in general, to untangle the cellular mechanisms by which sphingolipid metabolism disruption leads to CerTra syndrome. Nonetheless, the characterization of this rare disease entity and of the most frequent variants associated with it has already revealed unforeseen operating principles of sphingolipid homeostasis, led to the definition of a new structural region in CERT, and delineated a possible use of CERT inhibitors for the treatment of patients with CerTra.

## Methods

### Study participant identification and clinical characterization

Initial screening and identification of anonymized individuals harboring *CERT1* variation was conducted using the following public databases and tools: ClinVar (https://www.ncbi.nlm.nih.gov/clinvar/), Decipher (72), GeneMatcher (73), and VKGL-NL Rotterdam (https://www.vkgl.nl/nl/) (Supplemental Table 1). Each patient underwent a full clinical examination by a neurologist and/or medical geneticist. Clinical data were directly abstracted from medical records provided by the referring clinicians and included a behavioral assessment and EEGs. Developmental delay and cognitive ability tests were performed on patients using the following tests: Gesell Developmental Schedules, the Chinese National Health Commission Developmental Evaluation Scale, the Wechsler Intelligence Scale for Children, the Peabody Picture Vocabulary Test, 4th Edition (PPVT-4), and Bayley Scales of Infant Development II. When possible, a standardized assessment of impairment in conceptual, social, and practical domains for each patient was performed in accordance with the DSM-5 and noted to be mild, moderate, severe, or profound. The degrees of intellectual disability were classified according to the following verbal and nonverbal IQ scores: mild (IQ of 50–55 to ~70), moderate (IQ of 35–40 to 50–55), severe (IQ of 20–25 to 35–40), and profound (IQ below 20–25).

To characterize craniofacial/skeletal dysmorphia, we performed a deep phenotyping analysis for all of the patients whose families agreed to provide photographs. This was done by the team of Eva Bermejo-Sánchez at the Institute of Rare Disease Research (IIER) in Madrid, Spain. Dysmorphology analyses were performed by experimenters blinded to the variant detected for each patient. Each parameter was compared with age-, sex-, and ethnicity-matched healthy individuals. Briefly, a detailed reading of all the clinical reports was performed to extract a first list of dysmorphic features that was then used to evaluate all of the patients. All the available photographs were assessed on the basis of those features, giving a matrix of 96 rows that were scrutinized for all patients. Thus, the same traits were assessed by the same observer using homogeneous criteria for all the patients. See the Supplemental Clinical Appendix for all the features for each patient.

### Pathogenicity analysis

All identified variants were analyzed in accordance with American College of Medical Genetics and Genomics (ACMG) guidelines for variant interpretation and classification (74). Minor allele frequencies (MAFs) of all *CERT1* variants were obtained from gnomAD (75), BRowse All Variants Online (BRAVO) (https://bravo.sph.umich.edu), and 1000 Genomes Project (https://www.genome.gov/27528684/1000-genomes-project). Functional annotation of variants was carried out with ANNOtate VARiation (ANNOVAR) (76) using M-CAP (77), REVEL (78), Eigen (79), and CADD (79) pathogenicity scores. As a general guideline, pathogenicity is predicted for variants with scores over 0.025 for M-CAP, over 0.5 for REVEL, over 0.5 for Eigen, and over 20 for CADD. Prediction scores for CerTra variants were compared with other missense variants found in singleton cases among the general population in gnomAD (i.e., missense variants reported in only 1 healthy individual in gnomAD).

The analysis of variant clustering was performed as previously described (17, 80). In short, to determine whether the observed variants in patients (17 positions) cluster linearly more than expected compared with random permutations, we calculated the geometric mean distance (δg) between all missense variants on the *CERT1* gene. We calculated δg by taking the mean distance normalized for the transcript length of *CERT1* (hg19:NM_005713.3) over all variant combinations of *x_i_* and *x_j_* of the missense variants, where *x* represents the position for variants *i* and *j*, respectively. A total of 1 × 10^8^ permutations were performed, and statistical significance was determined (Fisher’s exact test) on the basis of how many times the permuted mean distance was smaller or equal to the mean geometric distance observed in our cohort (i.e., significant clustering was determined if the permuted mean distance was larger than what was observed in our cohort).

### Immunofluorescence staining and image analysis

HeLa cells were grown on glass coverslips that were treated according to the experimental protocol, fixed with 4% paraformaldehyde for 15 minutes at room temperature (RT), and washed 3 times with PBS. After fixation, cells were blocked with 5% BSA and permeabilized with 0.5% saponin for 20 minutes at RT, followed by a 1-hour incubation with selected antibodies against the antigen of interest in the blocking solution. Cells were then washed 3 times with PBS and incubated with the appropriate isotype-matched, Alexa Fluor–conjugated secondary antibodies diluted in blocking solution for 30 minutes. After immunostaining, cells were washed 3 times in PBS and once in water to remove salts. After Hoechst staining for nuclei, the samples were mounted with Fluoromount-G (Southern Biotech, 0100-01) on glass microscope slides and analyzed under a Leica SP8 confocal microscope with a 63× oil objective (1.4 NA),a Zeiss LSM700 with a 40× oil objective (1.3 NA), or a Stellaris-5 with a 40× oil objective (1.3 NA). Optical confocal sections were taken at 1 Airy unit under nonsaturated conditions with a resolution of 1,024 × 1,024 pixels and a frame average of 2. Images were then processed using Fiji software (https://imagej.net/Fiji) (81).

### Statistics

#### Experimental design.

For quantification of protein expression in patient-derived cell lines, values from at least 3 independent experiments were used. At every stage of the study, the experimenter was blinded to the identity of the control and patient-derived cell lines. For example, experimenter 1 made a list of samples and controls to be tested, and experimenter 2 randomized this list and relabeled the tubes; experimenter 2 was the only person with the key to identification of the samples. These samples were then distributed to experimenter 3 to culture the cells, and then to experimenter 1 to perform Western blot analysis, and finally, experimenters 1 and 4 analyzed the data. Only then was the key applied to identify the samples.

#### Software and statistical analysis.

Statistical significance (*P* < 0.05) was analyzed using GraphPad Prism 8 (GraphPad Software), Excel (Microsoft), and R-4.2.3 (The R Project for Statistical Computing). Statistical details and the number of replicates for each experiment can be found in the figures and legends. The range of expression levels in Western blots was determined from at least 3 independent experiments. The Student’s *t* test was used to compare the means between 2 groups, and ANOVA was used to compare the means among more than 2 groups.

### Study approval

All study procedures were defined under protocol no. AAAS7401, which was approved by the IRB of Columbia University Irving Medical Center (VAG); the Cambridgeshire Research Ethics Committee for the whole of the United Kingdom; the IRB “Commissie MensgebondenOnderzoek Regio Arnhem-Nijmegen” under protocol no. 2011/188; the Hospital Universitario “Virgen de la Arrixaca,” Wroclaw Medical University; San Gerardo Hospital (Monza, Italy); Children’s Health Ireland at Temple Street Hospital (Dublin, Ireland); the ethics committee of the Medical Faculty at Leipzig University (224/16-ek and 402/16-ek); and the ethics committee of the Instituto de Salud Carlos III.

Full study enrollment (i.e., acquisition of clinical data for further analysis) was conducted only after written, informed consent/assent was obtained for each study participant. The study also adhered to tenets outlined by the Declaration of Helsinki. All patient-related study procedures were conducted according to the respective ethics committees of each participating institution. Specific written, informed consent was also acquired for all participants whose photographs appear in the manuscript.

### Data availability

This study made use of data generated by the DEFIDIAG study sponsored by the INSERM (France). A full list of centers that contributed to the generation of the data is available from https://defidiag.inserm.fr/ and via email from c16-110.coordo.isp@inserm.fr. This work also used Simons Simplex Collection (SSC) data including whole-exome sequencing (raw and processed) and associated phenotypic data obtained through Simons Foundation Autism Research Initiative (SFARI) Base (SFARI Request ID: 12030.1.1). This study makes use of data generated by the DECIPHER community. A full list of centers that contributed to the generation of the data is available at https://deciphergenomics.org/about/stats and via email from contact@deciphergenomics.org.

## Author contributions

C Gehin, MAL, and LC designed and performed experiments, analyzed and interpreted the data, and drafted the manuscript. WL collected all clinical symptoms, analyzed and interpreted clinical data, and drafted the manuscript. SH supported experimental work and contributed to the writing of the manuscript. SZ and LAA performed structural simulations. VK provided recombinant PKD. BEM performed the HDX-MS. JEB performed and supervised HDX-MS. AV provided technical support. PH synthesized synthetic peptides. KH and TY provided *CERT1*-KO cell lines. PDLR supervised the structural simulations. BL, SR, JPB, and ER designed the *Drosophila* lines. BDM designed and supervised the behavioral tests with *Drosophila*. MVC designed and constructed the apparatus used for the negative geotaxis assay. SB supervised and supported the micro-CT experiment under the supervision of AMJ. YG, TYL, and SSC performed the ASD association analysis. EHG provided patient-derived fibroblasts and unaffected, healthy age- and sex-matched fibroblasts for participant 14. ELM and EBS performed craniofacial/skeletal deep phenotyping analysis. EHG, APAS, JHS, HG, KX, QZ, SX, CRF, CPB, MJL, MB, NSC, VW, ELM, EBS, BMD, RO, BP, VS, DG, MM, AS, AT, YH, MHW, FS, A Murgia, EL, RS, CG, RP, CZ, CK, VLG, LAD, IK, ERJ, IV, PFA, AG, ALB, FTMT, GC, PK, AMA, A Marwaha, NLC, MJF, EBR, VKG, VMS, BWVB, and MAB provided clinical data, genetic information, variant revalidation, and comments on the manuscript. ABRM and JMB interpreted and analyzed the MRI data. TH, GD, and VAG conceived the study, analyzed and interpreted the data, and wrote the manuscript. All authors approved the final version of the manuscript.

## Supplementary Material

Supplemental data

Supplemental data set 1

Supplemental data set 2

Supplemental data set 3

Supplemental data set 4

Supplemental data set 5

Supplemental data set 6

Supplemental data set 7

Supplemental tables 1-4

Supplemental table 5

Supplemental tables 6-7

## Figures and Tables

**Table 1:**
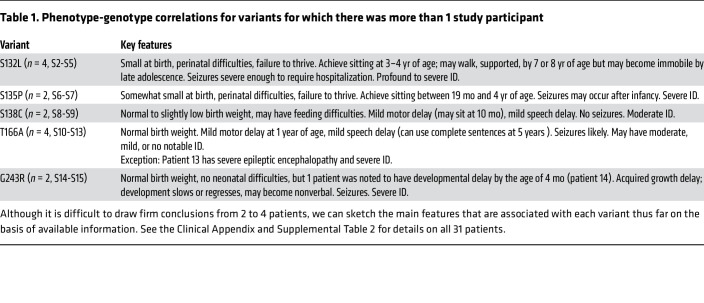
Phenotype-genotype correlations for variants for which there was more than 1 study participant

## Version 1 03/28/2023

In-Press Preview

## Version 2 05/15/2023

Electronic publication

## Version 3 11/03/2025

Upddated text and reference per corrigendum
