# Corrigendum to CERT1 mutations perturb human development by disrupting sphingolipid homeostasis

**DOI:** 10.1172/JCI200195

**Published:** 2025-11-03

**Authors:** Charlotte Gehin, Museer A. Lone, Winston Lee, Laura Capolupo, Sylvia Ho, Adekemi M. Adeyemi, Erica H. Gerkes, Alexander P.A. Stegmann, Estrella López-Martín, Eva Bermejo-Sánchez, Beatriz Martínez-Delgado, Christiane Zweier, Cornelia Kraus, Bernt Popp, Vincent Strehlow, Daniel Gräfe, Ina Knerr, Eppie R. Jones, Stefano Zamuner, Luciano A. Abriata, Vidya Kunnathully, Brandon E. Moeller, Anthony Vocat, Samuel Rommelaere, Jean-Philippe Bocquete, Evelyne Ruchti, Greta Limoni, Marine Van Campenhoudt, Samuel Bourgeat, Petra Henklein, Christian Gilissen, Bregje W. van Bon, Rolph Pfundt, Marjolein H. Willemsen, Jolanda H. Schieving, Emanuela Leonardi, Fiorenza Soli, Alessandra Murgia, Hui Guo, Qiumeng Zhang, Kun Xia, Christina R. Fagerberg, Christoph P. Beier, Martin J. Larsen, Irene Valenzuela, Paula Fernández-Álvarez, Shiyi Xiong, Robert Śmigiel, Vanesa López-González, Lluís Armengol, Manuela Morleo, Angelo Selicorni, Annalaura Torella, Moira Blyth, Nicola S. Cooper, Valerie Wilson, Renske Oegema, Yvan Herenger, Aurore Garde, Ange-Line Bruel, Frederic Tran Mau-Them, Alexis B.R. Maddocks, Jennifer M. Bain, Musadiq A. Bhat, Gregory Costain, Peter Kannu, Ashish Marwaha, Neena L. Champaigne, Michael J. Friez, Ellen B. Richardson, Vykuntaraju K. Gowda, Varunvenkat M. Srinivasan, Yask Gupta, Tze Y. Lim, Simone Sanna-Cherchi, Bruno Lemaitre, Toshiyuki Yamaji, Kentaro Hanada, John E. Burke, Ana Marija Jakšić, Brian D. McCabe, Paolo De Los Rios, Thorsten Hornemann, Giovanni D’Angelo, Vincenzo A. Gennarino

Original citation: *J Clin Invest*. 2023;133(10):e165019. https://doi.org/10.1172/JCI165019

Citation for this corrigendum: *J Clin Invest*. 2025;135(21):e200195. https://doi.org/10.1172/JCI200195

The authors recently became aware of inadvertently omitted text and a reference citation on page 8. The correct text is provided below.

Circular dichroism confirmed that purified CERT 151–309, hereafter referred to as the CERT central core domain (CCD) in reference to a structurally analogous region characterized in the lipid transfer protein OSBP (47), and the synthetic peptides of H1 and H2 were indeed helical; the CCD had 60% helical content and a melting temperature of 43°C (Figure 5D).

The HTML and PDF files have been updated.

The authors regret the error.

## Version 1 11/03/2025

Electronic publication

