# Supplementary material for: CERT1 mutations perturb human development by disrupting sphingolipid homeostasis
Source: J Clin Invest. 2023 Mar 28;133(10):e165019. doi: 10.1172/JCI165019 (PMC10178846; doi:10.1172/JCI165019)
Supplement: Supplemental data [file jci-133-165019-s008.pdf]

Supplemental Data

CERT1 mutations perturb human development by disrupting sphingolipid homeostasis

Table of Contents

Supplemental Figures and Legends…………………………………………..………………..……….1-7

List of Supplemental Tables and Files legends………………………………………..…………………….8

Supplemental Methods………..…………………………………………………………..…....……9-19

Clinical Appendices……………………………………………………………………..…...……20-41

References………………………………………………………………………………………41-42

Supplemental Figures

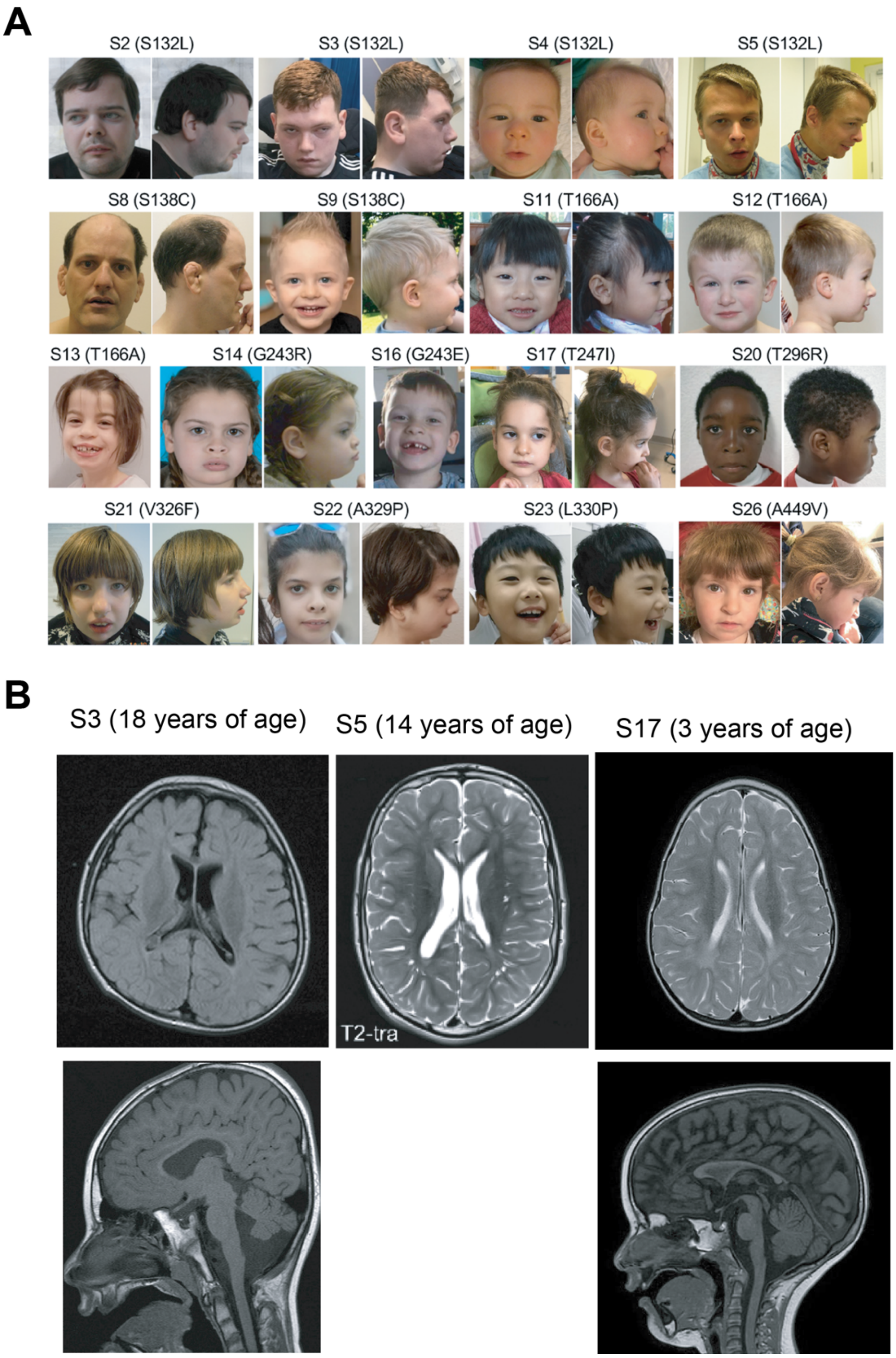

Supplemental Figure 1. Characterisation of CerTra patients. (A) Frontal and lateral facial photographs of affected subjects (age in parentheses). Subject 2 (24 years); Subject 3 (18 years); Subject 4 (1 year); Subject 5 (17 years); Subject 8 (50 years); Subject 9 (1 year); Subject 11 (3 years); Subject 12 (5 years); Subject 13 (8 years); Subject 14 (8 years); Subject 16 (5 years); Subject 17 (4 years); Subject 20 (7 years); Subject 21 (12 years); Subject 22 (8 years); Subject 23 (5 years); Subject 26 (2 years, 6 months). **(B)** Representative brain MRI images from Subjects 3, 5 and 17. (S3) Sagittal (*left*) and axial (*right*) T1 weighted images demonstrate thinning of the corpus callosum along with cerebellar atrophy and mild ventriculomegaly. (S5) The axial T2 weighted image demonstrates multiple T2 hyperintensities within the white matter, some of which present like microcysts which likely represent prominent perivascular spaces. (S17) Sagittal (*left*) T1 and axial (*right*) T2 weighted images show thinning of the corpus callosum as well as relative paucity of white matter with abnormal T2 hyperintensity most marked at the periatrial regions.

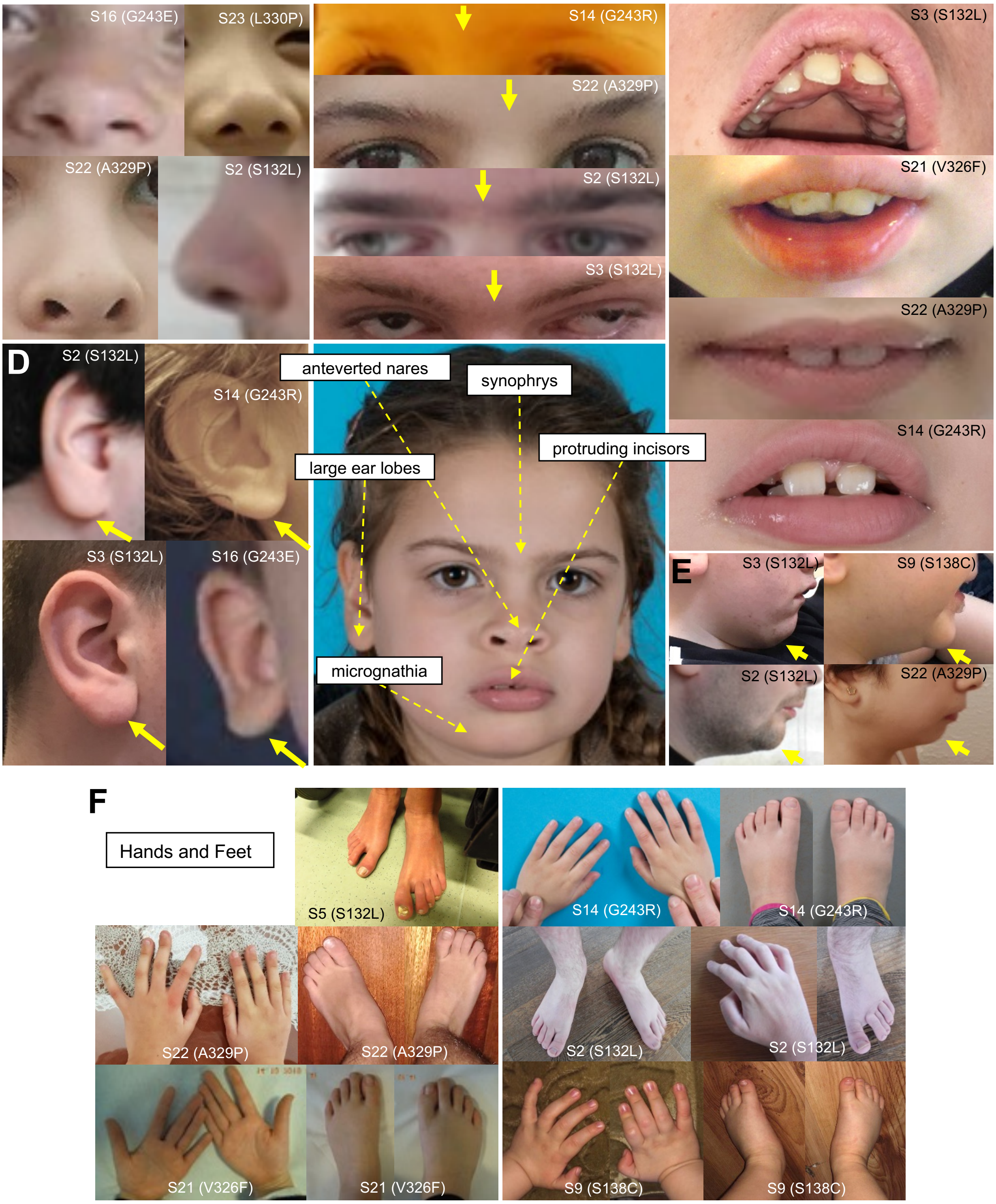

Supplemental Figure 2. Craniofacial and peripheral dysmorphologies in CerTra subjects. Enlargement of craniofacial dysmorphologies in patients showing: **(A)** anteverted nares, **(B)** synophrys, **(C)** protruding incisors, **(D)** large ear lobes, **(E)** micrognathia, and **(F)** hand and foot abnormalities.

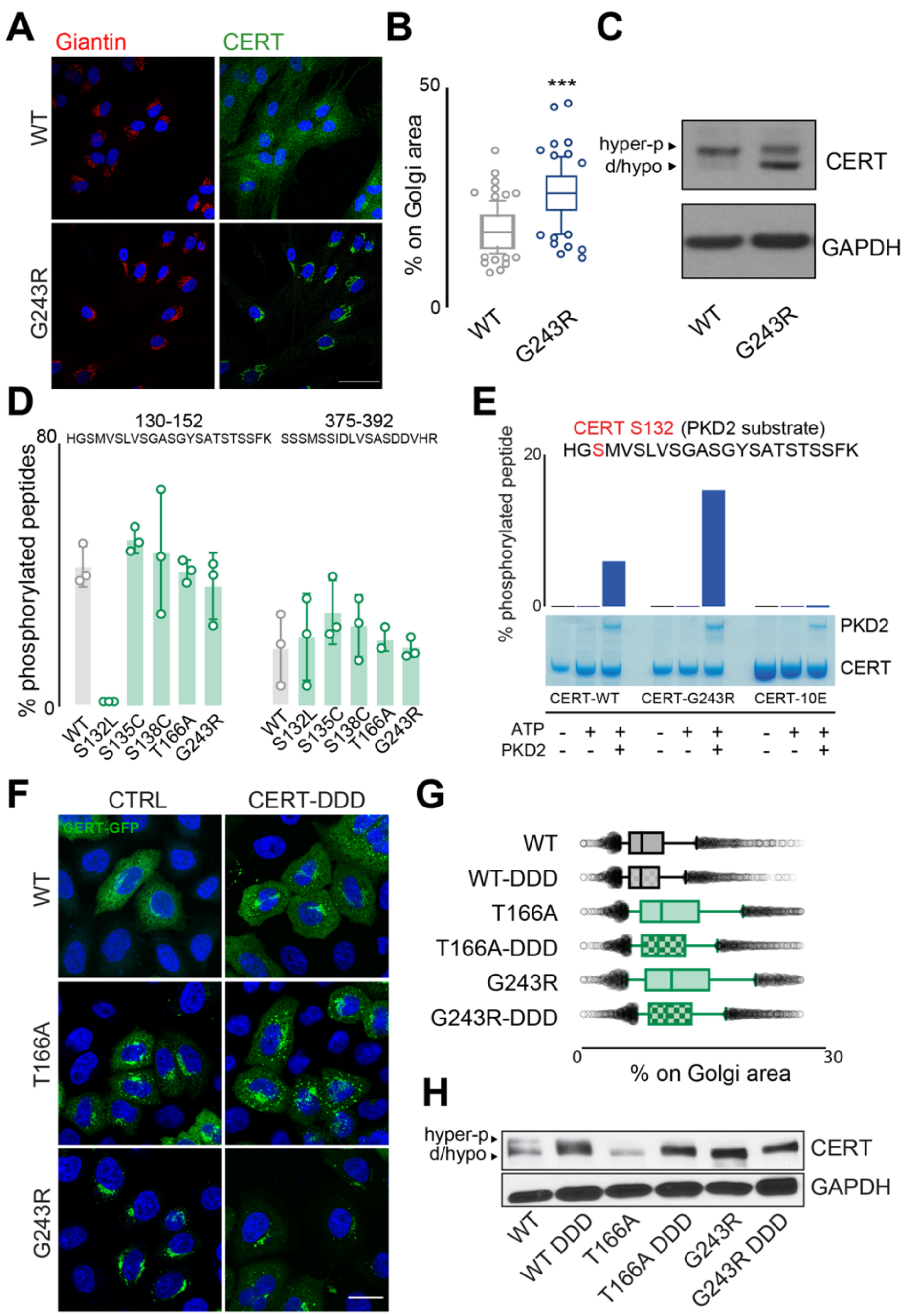

Supplemental Figure 3. Phosphorylation assessment of CERT mutants. (A) Steady-state localization of endogenous CERT in human fibroblasts from a healthy individual and from Subject 14 (p.G243R). Fibroblasts were stained with Hoechst, anti-Giantin (red), and anti-CERT antibodies (green) and analysed by confocal microscopy. Scale bar, 50 µm. **(B)** Percentage of endogenous CERT associated with the Golgi complex in WT fibroblasts and those bearing the p.G243R mutation (n=85 and 88 for WT and Mutant; ***p <0.001 [Student's t-test]). **(C)** Western blot analysis of WT and p.G243R mutant human fibroblasts. **(D-E)** Percentage of phosphorylated peptides detected in immunoprecipitated CERT-GFP WT and mutants expressed in HeLa cells (n=3; data are means ± SD) **(D)**, or in recombinant CERT WT, p.G243R or 10E in the presence of purified PKD2 and ATP (n=3; data are means ± SD) **(E)**. **(F)** Localization of CERT-GFP WT and DDD mutants expressed in HeLa cells. Cells were stained with Hoechst and anti-GOLPH3 antibody and analysed by confocal microscopy. Scale bar, 20 µm. **(G)** Percentage of CERT-GFP WT and mutants associated with the Golgi complex in HeLa cells. Cells were stained with Hoechst and anti-GM130 antibody and analysed by automated fluorescence microscopy (n>3,000 cells per condition). CERT WT in grey, CERT1 mutants in green, Bars represent the median value of each dataset. **(H)** Western blot analysis of HeLa cells expressing CERT-GFP WT or DDD mutants.

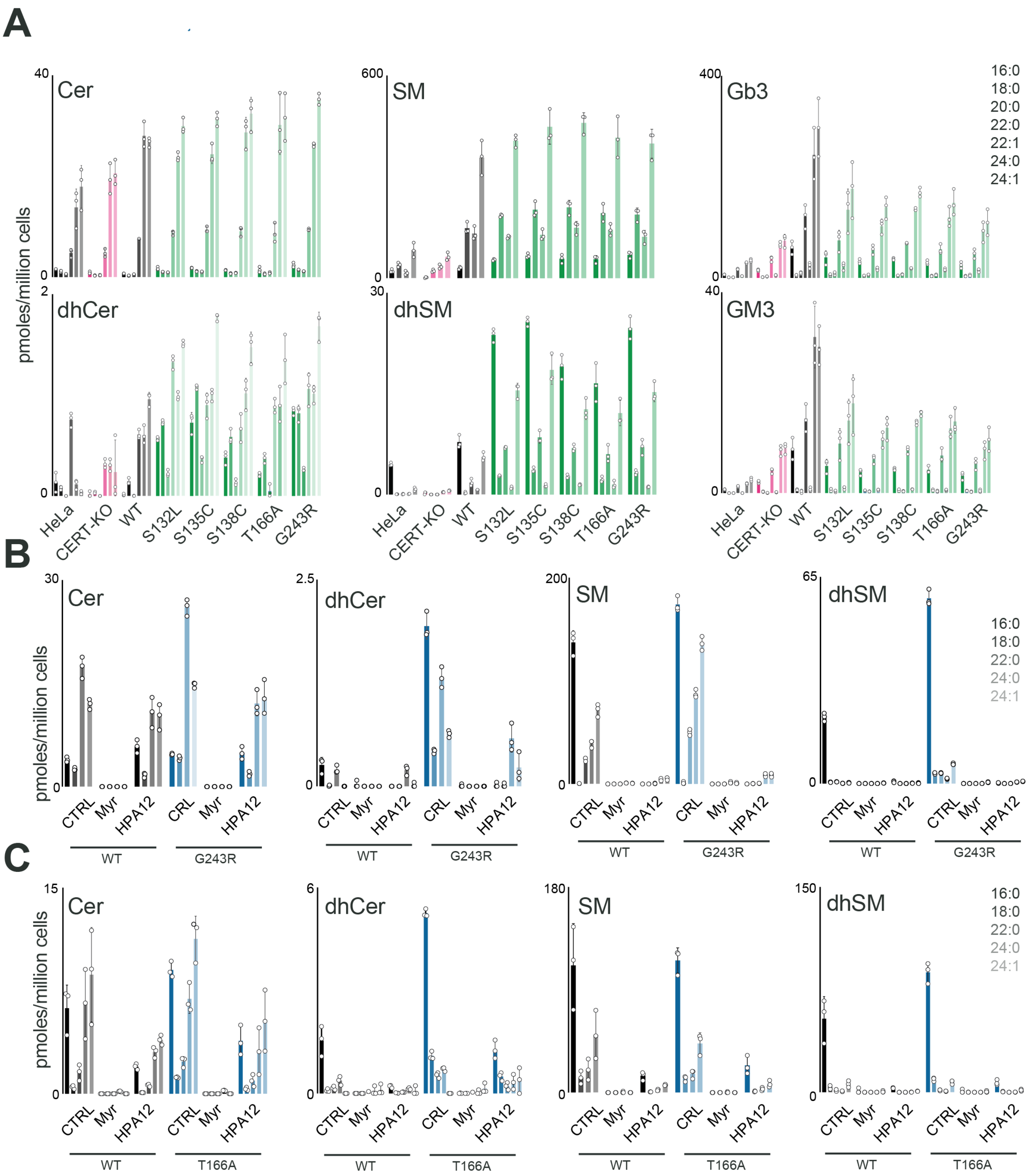

Supplemental Figure 4. *CERT1*-induced sphingolipid change and rescue by CERT inhibitor: acyl chain composition. (**A**) Mass spectrometry profile of sphingolipids in Hela cells overexpressing selected CERT mutants from Cluster 1, 2, and 3. (**B**) Mass spectrometry profile of sphingolipids in WT and p.G243R human fibroblasts treated with myriocin or HPA-12. (**C**) Mass spectrometry profile of sphingolipids in WT and p.T166A human lymphoblasts treated with myriocin or HPA-12. Acyl chain composition of each sphingolipid class is represented by a gradient of colours: 18:1/16:0, 18:1/18:0, 18:1/20:0, 18:1/22:0, 18:1/22:1, 18:1/24:0 and 18:1/24:1, from darkest to lightest shade. Cer, ceramide; dhCer, dihydro-ceramide; SM, sphingomyelin; dhSM, dihydro-sphingomyelin; Gb3, globotriaosylceramide; GM3, monosialodihexosylganglioside. (n=3; data are means ± SD).

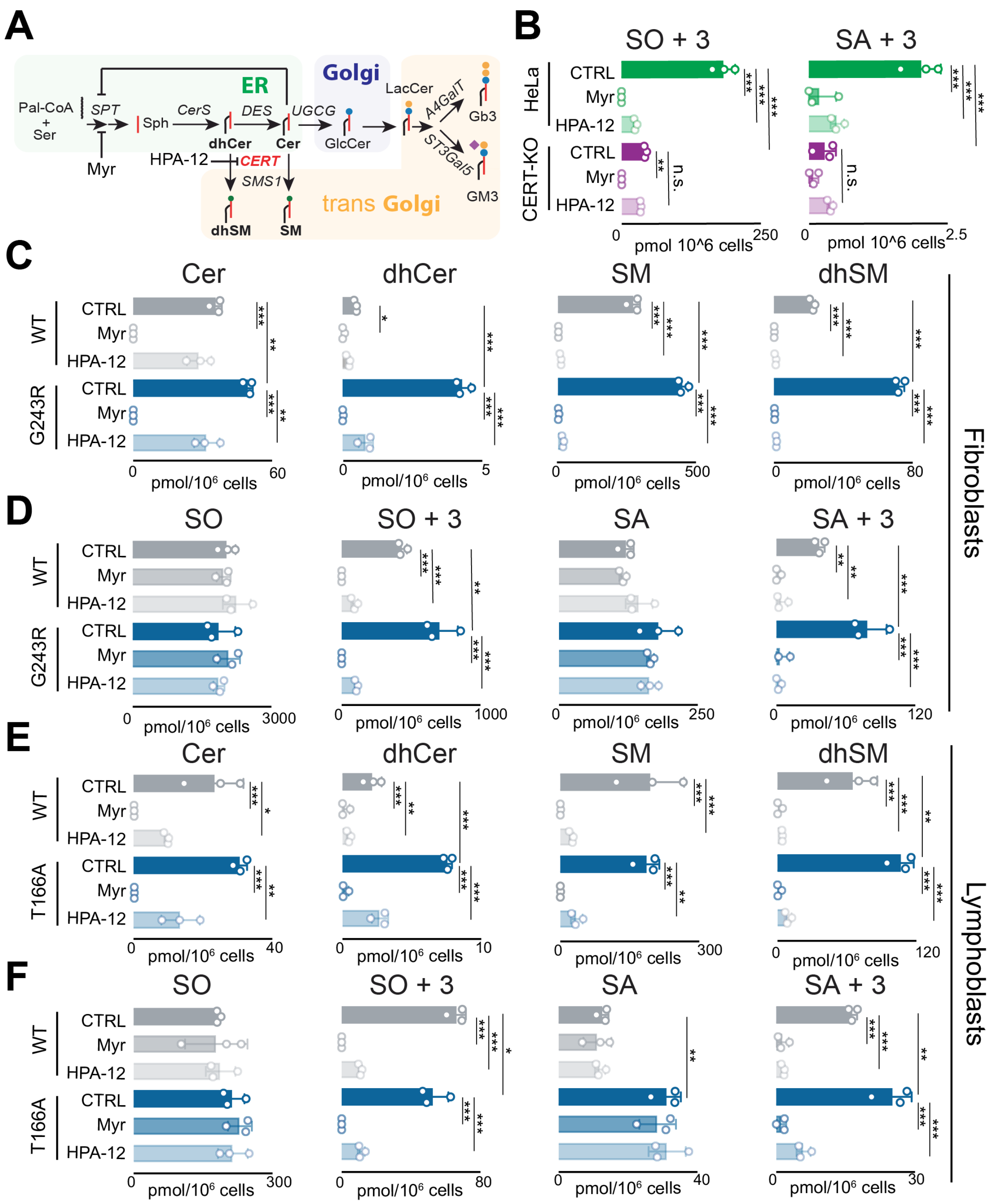

Supplemental Figure 5. Rescue of *CERT1* mutant-induced sphingolipid changes by CERT inhibition. (A) Diagram of the *de novo* sphingolipid biosynthetic pathway indicating the sites of action of Myr and HPA-12 inhibitors. The homeostatic negative feedback loop involving ER Cer in the inhibition of the *SPT* enzyme is also depicted. **(B)** Mass spectrometry profile of LCBs C18SO and C18SA in *CERT1*-KO HeLa cells after inhibition by HPA-12. LCBs were evaluated following incorporation of (2,3,3-D^3, ^{15}N)- L-serine. Cells treated with myriocin were used as control. **(C)** Mass spectrometry profile of sphingolipids in WT and p.G243R fibroblasts treated with myriocin or HPA-12. **(D)** Mass spectrometry profile of long chain bases incorporation in WT and p.G243R human fibroblasts treated with myriocin or HPA12. **(E)** Mass spectrometry profile of sphingolipids in WT and p.T166A human lymphoblasts treated with myriocin or HPA-12. **(F)** Mass spectrometry profile of long chain base incorporation in WT and p.T166A human lymphoblasts treated with myriocin or HPA-12. (n=3; data are means ± SD; *p <0.05, **p <0.01; ***p <0.001 [Ordinary one-way ANOVA]).

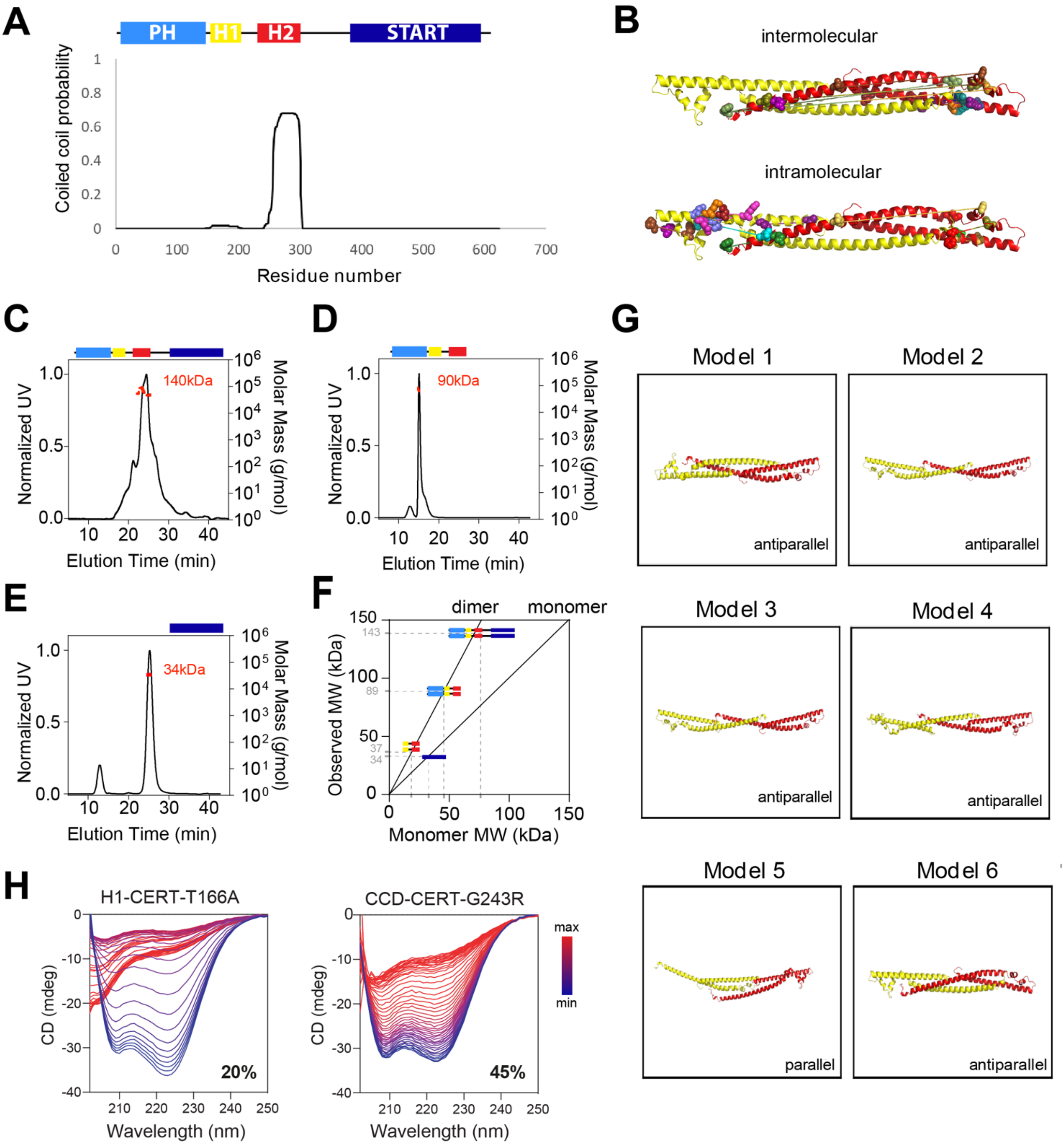

Supplemental Figure 6. Characterization of the central core structure of CERT. (A) Coiled coil probability of full-length CERT WT obtained using Multicoil 2. A schematic representation of CERT domain organisation is showed on top. **(B)** Molecular modelling of CERT CCD. **(C)** SEC-MALS profile of full-length recombinant CERT WT, **(D)** PH-CCD domains (aa 1-341), **(E)** START domain (aa 389-618), **(F)** correspondence between expected and observed molecular weight for the different CERT constructs. The different regions of CERT are represented in blue (PH), red (H1), yellow (H2) and dark blue (START). **(G)** The best six molecular models (from 1 to 6) of the CERT CCD dimer by rigid docking. **(H)** Thermostability of CERT 151-187 p.T166A (*left*) and CERT 151-309 p.G243R (*right*) by circular dichroism; colour scale indicates temperature ranging from 4°C to 94°C.

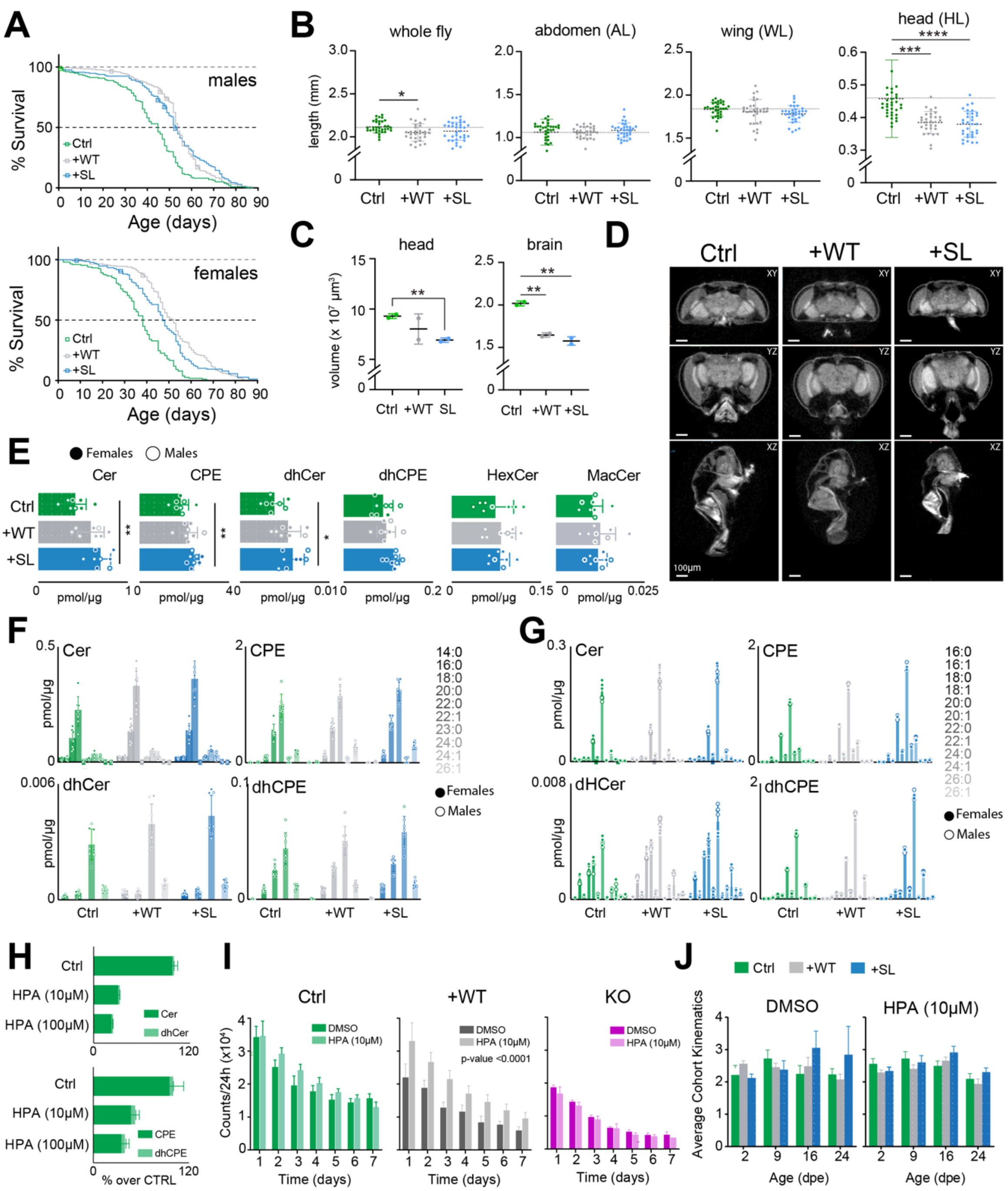

Supplemental Figure 7. Characterisation of *dCERT* gain of function in *Drosophila melanogaster*. (A) Lifespan of Ctrl, +WT, and +SL male (top, n=166, 238, 217) and female (bottom, n=166, 199, 148) flies maintained on standard medium at 25oC. **(B)** Raw measurement of Ctrl (n=33), +WT (n=30) and +SL (n=33) whole fly and body parts [abdomen (AL), wing (WL) and head (HL)] lengths. **(C)** Quantification of head and brain volumes from µ-CT scans of Ctrl (n=2), +WT (n=2) and +SL (n=2) flies (**p <0.01, [Ordinary one-way ANOVA]). **(D)** Top XY (top), Frontal YZ (middle) and Sagittal XZ (bottom) view of Ctrl, +WT, and +SL flies scanned with µ-CT at 3.3 µm resolution. **(E)** Mass spectrometry profile of sphingolipids in Ctrl, +WT, and +SL male (n=4) and female (n=4) flies; Cer, ceramide; dhCer, dihydro-ceramide; CPE, ceramide phosphoethanolamine; dhCPE, dihydro-ceramide phosphoethanolamine; HexCer, hexosylceramide; MacCer, mactosylceramide; (*p <0.05, **p <0.01; [Ordinary one-way ANOVA]). **(F)** Acyl chain composition of each sphingolipid class of Ctrl, +WT, and +SL male and female heads and, **(G)** entire flies represented by a gradient of colours. **(H)** Mass spectrometry profile of sphingolipids in Ctrl males treated with DMSO, 10 µM or 100 µM HPA-12. Data shown are the percentage of untreated flies. **(I)** Locomotor activity of Ctrl, +WT, and KO adult male flies over 7 days; n= 8 per group. **(J)** Quantification of the climbing ability of *dCERT* transgenic flies under normal conditions. Data shown is mean ± SEM.

Supplemental Table and File Legends:

Supplemental Table 1. Genetic and phenotypic summary of other subjects with *CERT1* variants extracted from various public access databases and not included in this project.

Supplemental Table 2. Summary of molecular and clinical symptoms of 31 individuals with *CERT1* mutations.

Supplemental Table 3. Summary of sequencing methodology and results of other genetic analyses.

Supplemental Table 4. Pathogenicity score calculated for variants in the cohort compared to healthy singleton controls in gnomAD.

Supplemental Table 5. Raw HDX-MS data for CERT WT and p.G243R

Supplemental Table 6. Data processing statistics for HDX-MS analysis

Supplemental Table 7. List of site-directed mutagenesis and qPCR Oligos used in this study

Supplemental Files 1-6. Atomic coordinates of CCD dimer models shown in Supplementary Figure 6

Supplemental Files 7. Atomic coordinates CERT dimer models shown in Figure 5

Supplemental Methods

Sequencing and genetic analyses

A table summarising all genetic testing procedures and results is provided in Supplemental Table 4. Before the identification of *CERT1* mutations by trio or singleton exome sequencing, most patients underwent extensive genetic screenings that included karyotyping, chromosomal microarray analysis (CMA), FMR1 repeat expansion and metabolic testing, none of which were significant. *CERT1* variants were identified by either research or clinical diagnostic whole exome sequencing. Methodology and analysis pipelines for each are provided in detail in Supplemental Table 4. Allelic segregation was verified in all except four subjects (S1, S19, S20 and S24) for whom familial samples were not available.

SPARK database analysis

Quality-controlled processed VCF files (for exomes) were obtained from the SPARK database and the genomic region on chromosome 5 harbouring *CERT1* was extracted. The processing and analysis of raw sequencing data is described elsewhere (1). In brief, for exomes, genotypes for 15,995 individuals from 10,686 families were obtained from SPARK database. 251 variants (SNPs and INDELs) within *CERT1* (chromosome 5: 75368438 – 75511667, Hg19) were extracted from the VCF files using BCFTools (2). Eight multi-allelic variants were filtered out using PLINK2 (3) resulting in 243 bi-allelic variants within this cohort. The Simons collection consists of trios and quads of affected individuals that span multiple neurological disorders including ASD and Asperger's syndrome. Using the phenotype information file provided by the SPARK database, we extracted 1,488 full trios (healthy parents, affected child) and 220 Quads (healthy parents, one affected child and the other not) for ASD. Next, the genotypes of the ASD individuals were compared to their parents to identify potential *de novo* mutations. Due to NGS-related genotyping errors and the rarity of *de novo* mutations, any *de novo* variant observed more than two times in different trios and quads was discarded. One intronic mutation (rs759188375, 5:75410964_T/C) was found within one quad. Exomes and genomes were investigated for *de novo* mutations in Simons collection using denovo-db (v.1.6.1).

Cell lines and culture conditions

Unaffected healthy primary skin human fibroblasts (HF) and HF from patients with CERT p.G243R mutation (Subject 14) were provided by Erica H. Gerkes (University of Groningen). Cells were grown in DMEM high glucose, GlutaMAX™ (Gibco, USA) supplemented with 10% (v/v) foetal bovine serum (FBS), 4.5 g/L glucose, 2 mM L-glutamine, 1 U/mL penicillin and streptomycin under controlled atmosphere (5% CO_2 and 95% air) at 37°C. Lymphoblastoid suspension cell cultures from the patient with p.T116A were provided by Christiane Zweier and Hui Guo (Friedrich-Alexander-Universität Erlangen-Nürnberg and Central South University). Cells were grown in RPMI 1640 medium (Invitrogen, #11875093) supplemented with 10% heat-inactivated fetal bovine serum (Atlanta Biological, Flowery Branch, #S11195H) and 1% penicillin/streptomycin (GenDepot, #CA005-010). Cell cultures were maintained at 37°C in a humidified

incubator supplemented with 5% CO_2. Medium was renewed every 2 to 3 days.

Drug treatments

HeLa cells were treated with 2.5 µM myriocin (stock solution at 5 mM in DMSO, LabForce AG sc-359032) in complete media for 15 hours. HeLa Cat-8 and HeLa *CERT1*-KO were treated with 1 µM myriocin (2.5 mM, Focus biomolecules, 10-2088) and 10 µM HPA-12 (Cayman Chemical Company 28350, 10 mM stock) for the duration of the SPT activity assay.

Plasmid transfection

Human CERT WT and mutants were inserted into pEGFP-C1 vector (Clontech) to produce protein with eGFP at the N-terminus. Standard molecular biology techniques were used to introduce mutations in the GFP-CERT wild type plasmid by site-directed mutagenesis, see Table 7 for the primer sequences. The other plasmids where the primers are not listed were acquired from Genscript Biotech Corporation. Human CSNK1G2 and PP2Cε were inserted into pcDNA6.2 3HA DEST to produce protein with HA tag at the N-terminus. Plasmids were transfected into HeLa cells with jetPRIME transfection reagent (Polyplus Transfection, 114-15) following the manufacturer's instructions.

Antibodies

The following primary antibodies were used: rabbit anti-COL4A3BP (Sigma Aldrich, HPA035645, RRID: AB_10600700, 1:5,000 for WB, 1:300 for IF), mouse anti-giantin Clone 9B6 (Abcam ab37266, RRID: AB_ 880195, 1:300), mouse anti-GM130 Clone 35 (BDbiosciences, BD 610823, RRID: AB_398142, 1:500), rabbit anti-GOLPH3 (Abcam ab98023, RRID: AB_10860828, 1:300), mouse anti-GAPDH Clone 6C5 (Santa Cruz Biotechnology, sc-32233, RRID: AB_627679, 1:2,000), mouse anti-HA.11 Epitope Tag Antibody (Biolegend, 901516, RRID: AB_2820200, 1:400), rat anti-HA high affinity Clone 3F10 (Roche, 11867423001, RRID: AB_390918, 1:100). The following secondary antibodies were used: donkey A568-conjugated anti-mouse (ThermoFisher Scientific, A-10037, RRID: AB_2534013, 1:400), donkey A568-conjugated anti-rabbit (ThermoFisher Scientific, A-10042, RRID: AB_2534017, 1:400), donkey A647-conjugated anti-mouse (ThermoFisher Scientific, A-31571, RRID: AB_162542, 1:400 or Jackson ImmunoResearch, 715.605.150, AB_2340862, 1:200 for quantitative image analysis), donkey Cy3 anti-rat (Jackson ImmunoResearch, 712.165.153, RRID: AB_2340667, 1:200), donkey HRP-conjugated anti-rabbit (Jackson ImmunoResearch, 711-035-152, RRID: AB_10015282, 1:10,000), and donkey HRP-conjugated anti-mouse (Jackson ImmunoResearch, 715-035-150, RRID: AB_2340770). Hoechst was purchased from Life Technologies (H3570, 1:2,000 from 10mg/mL stock).

Quantitative cell imaging

HeLa cells were seeded in a µ-Plate 96 Well Black (IBIDI, 89626) at a concentration of 8 x 10^4cells/well for 48 h transfection experiment or 1.2x10^4 cells/well for 16 h transfection experiment. CERT-GFP WT or mutant plasmids were transfected using TransIT-X2® Transfection Reagent (Mirus Bio, MIR 6000). Cells

were fixed in 3% paraformaldehyde for 20 min before staining with antibodies diluted in a PBS solution of 1% BSA and 0.05% saponin. After washing with an automated plate washer (BioTek EL406), cells were incubated for half an hour with appropriate secondary antibodies and nuclei were stained by Hoechst. Cells were left in PBS and imaged by ImageXpress® Micro Confocal microscope (Molecular Devices, Sunnyvale, CA); for each well, 49 frames were taken in widefield mode with a 40X objective. Images were quantified using MetaXpress Custom Module editor software to first segment the image and generate relevant masks, which were then applied on the fluorescent images to extract relevant measurements.

SDS-PAGE and Western blotting

After treatment, the cells were washed three times with PBS and lysed in a buffer consisting of 20 mM MOPS pH 7.0, 2 mM EGTA, 5 mM EDTA, 60 mM β-glycerophosphate, 30 mM NaF, 1 mM Na_3VO_4, 1% (v/v) Triton X-100, phosphatase inhibitor (PhosSTOP™, Sigma-Aldrich) and protease inhibitor (cOmplete™ Protease Inhibitor Cocktail, EDTA free, Roche). The lysates were clarified by centrifugation and quantified with Pierce™ BCA Protein Assay Kit (ThermoFisher) according to the manufacturer's instructions. Samples were prepared by adding 4x SDS sample buffer, denatured at 95°C for 5 min and resolved by SDS-PAGE and immunoblot. For immunoblotting, the membrane strips containing the proteins of interest were blocked in TBS-T/ 5% BSA for 45 min at RT, and then incubated with the primary antibody diluted to its working concentration in the blocking buffer for 1h at RT. After washing with TBS-T, the strips were incubated for 1 h with the appropriate HRP-conjugated secondary antibody diluted in the blocking buffer. After washing with TBS-T, the strips were incubated with the ECL solution for 3 min and exposed to x-ray films, which were then scanned. The intensity of the bands and preparation of images was done using Fiji (4) and Adobe Illustrator 2020.

Recombinant protein expression and purification

Human CERT WT and mutants were expressed using pET29b (+) vector (Novagen) to produce protein with His6 plus Twin-Strep tags at the N-terminus. All point mutations were generated using the QuikChange Lightning Kit (Agilent Technologies). The CERT fragments were amplified from the full-length vectors and subcloned into pET29b (+) via NcoI/XhoI. CERT WT and mutant full-length and fragment proteins were expressed and purified as follows: briefly, BL21 [DE3] (Sigma-Aldrich, CMC0014-20X40UL) harbouring the appropriate plasmids were grown at 37°C to OD600 = 0.4 to 0.6. Protein expression was induced by the addition of IPTG to 0.2mM and cultures were grown for an additional 16 h at 18°C. Cells were lysed by sonication in Lysis Buffer (50 mM Tris-HCl pH7.4, 250 mM NaCl, 10% (v/v) glycerol, 0.5 mM DTT, Protease Inhibitor Cocktail, benzonase), and clarified by centrifugation at 20,000 *x g* for 30 min at 4°C. The supernatant was loaded on HisTrap Excel (GE Healthcare Life Sciences), and protein was eluted in 50 mM Tris-HCl pH7.5, 500 mM NaCl, 500 mM Imidazole, 0.5 mM DTT. Full-length and fragment proteins were polished on HiLoad 16/600 Superdex 200pg and HiLoad 16/600 Superdex 75pg, respectively.

PKD *in vitro* kinase assay

The PKD *in vitro* kinase assay was performed as described elsewhere (5) with minor modifications. Each 30 µL reaction contained 50 mM Tris pH 7.4, 10 mM $MgCl_2$, 100 µM ATP, 2 mM dithiothreitol, 5 µg purified CERT protein and 1 µg purified recombinant PKD2 kinase (kind gift of Dr. Alberto Luini, ICB, Naples, Italy). The mixture was incubated for 30 min at 37°C, and then the reactions were stopped by adding 10 µL of 4x SDS sample buffer. After SDS-PAGE, the gels were stained with Coomassie Blue and the indicated protein bands were excised for mass spectrometry analysis.

Immunoprecipitation assays

HeLa cells transfected with the indicated GFP-tagged plasmids were lysed in 20 mM MOPS pH 7.0, 2 mM EGTA, 5 mM EDTA, 60 mM β-glycerophosphate, 30 mM NaF, 1 mM Na3VO4, 1% (v/v) Triton X-100 and protease inhibitor. 1 mg total lysate was mixed with 1 µg of anti-GFP IgG1k (Roche, 11814460001, clones 7.1 and 13.1, RRID: AB_390913) overnight, and then incubated for 1 h with Dynabeads protein A (Invitrogen, 10002D). Proteins were eluted by adding sample buffer (0.125 M Trizma base, 4% SDS, 20% glycerol, 10% β-mercaptoethanol, pH 6.8), separated by SDS–PAGE, stained with Coomassie Blue and the indicated protein bands were subjected to mass spectrometry analysis.

Mass spectrometry sample preparation

SDS-PAGE gel pieces were washed twice in 50% ethanol (Merck-Millipore) and 50 mM ammonium bicarbonate (AB, Sigma-Aldrich) for 20 min and dried by vacuum centrifugation. Sample reduction was performed with 10 mM dithioerythritol (Merck-Millipore) for 1 h at 56°C. Samples were washed-dried as described above and then alkylated with 55 mM Iodoacetamide (Sigma-Aldrich) for 45 min at 37°C in the dark. Samples were washed-dried again and digested overnight at 37°C using modified mass spectrometry grade trypsin (Trypsin Gold, Promega) at a concentration of 12.5 ng/µl in 50 mM AB and 10 mM $CaCl_2$. Resulting peptides were extracted in 70% ethanol, 5% formic acid (FA, Merck-Millipore) twice for 20 min with continuous shaking. Samples were dried by vacuum centrifugation and stored at -20°C.

Peptides were desalted on C18 StageTips (6) and dried by vacuum centrifugation prior to LC-MS/MS injections. Samples were resuspended in 2% acetonitrile (Biosolve), 0.1% FA and nano-flow separations were performed on a Dionex Ultimate 3000 RSLC nano UPLC system (ThermoFischer Scientific) on-line connected with a Q Exactive Orbitrap Mass Spectrometer (ThermoFischer Scientific) or Q Exactive HF Orbitrap Mass Spectrometer (ThermoFischer Scientific). A capillary precolumn (Acclaim Pepmap C18, 3 µm-100Å, 2 cm x 75 µm ID) was used for sample trapping and cleaning. A capillary column (75 µm ID; in-house packed using ReproSil-Pur C18-AQ 1.9 µm silica beads; Dr. Maisch; length 50 cm or 15 cm) was then used for analytical separations at 250 nL/min over 90 min biphasic gradients.

Mass spectrometry data analysis

Raw data were processed using SEQUEST, MS Amanda and Mascot in Proteome Discoverer v.2.4 against a concatenated database consisting of the Uniprot human reference proteome (Release 2019_06, 97766 sequences) and CERT sequences. Enzyme specificity was set to trypsin and a minimum of six amino acids was required for peptide identification. Up to two missed cleavages were allowed. For the search, carbamidomethylation was set as a fixed modification, whereas oxidation (M), acetylation (protein N-term), PyroGlu (N-term Q), and Phosphorylation (S,T,Y) were considered as variable modifications. Data was further searched using X! Tandem and inspected in Scaffold 4.11.1 (Proteome Software, Portland, USA). Spectra of interest were manually validated.

HDX-MS

HDX reactions comparing CERT WT and p.G243R mutant were conducted in 50µL reaction volumes at a final concentration of 0.58 µM. Exchange was initiated by addition of 48.2 µL of D_2O solution (20 mM HEPES pH 7.2, 100 mM NaCl, 94.3%D_2O) for a final concentration of 90.9% D_2O. Deuterium exchange reactions were carried out for 3, 30, 300, 3000 s at 20°C. Exchange was stopped by the addition of 20 µL of ice cold acidic quench buffer at a final concentration of 0.6M guanidine-HCl and 0.9% formic acid. Samples were then immediately frozen in liquid nitrogen until mass analysis. Protein samples were rapidly thawed and injected onto an integrated fluidics system containing a HDx-3 PAL liquid handling robot and climate-controlled (2°C) chromatography system (LEAP Technologies), a Dionex Ultimate 3000 UHPLC system, as well as an Impact HD QTOF Mass spectrometer (Bruker) (6). The protein was run over two (one at 10°C and the other at 2°C) immobilised pepsin columns (Trajan; ProDx protease column, 2.1 mm x 30 mm PDX.PP01-F32) at 200 µL/min for 3 min. The resulting peptides were collected and desalted on a C18 trap column (Acquity UPLC BEH C18 1.7mm column (2.1 x 5 mm); Waters 186003975). The trap was subsequently eluted in line with an ACQUITY 1.7 µm particle, 100 × 1 mm2 C18 UPLC column (Waters), using a gradient of 3-35% B (Buffer A 0.1% formic acid; Buffer B 100% acetonitrile) over 11 min immediately followed by a gradient of 35-80% over 5 min. Mass spectrometry experiments were acquired over a mass range from 150 to 2200 m/z using an electrospray ionisation source operated at a temperature of 200°C and a spray voltage of 4.5 kV. The resulting MS/MS datasets were analysed using PEAKS7 (PEAKS), and a false discovery rate was set at 1% using a database of purified proteins and known contaminants (7). HDExaminer Software (Sierra Analytics) was used to automatically calculate the level of deuterium incorporation into each peptide. All peptides were manually inspected for correct charge state and presence of overlapping peptides. Deuteration levels were calculated using the centroid of the experimental isotope clusters. Differences in exchange between WT and p.G243R in any peptide were considered significant if they met all three of the following criteria: >5% change in exchange, >0.4 Da difference in exchange, and a p value <0.01 using a two tailed student t-test.

All HDX-MS raw data is available in Supplementary Table 4; data analysis statistics are in Supplementary Table 5 according to the guidelines (8). The mass spectrometry proteomics data have been

deposited in the ProteomeXchange Consortium via the PRIDE partner repository (8) with the dataset identifier PXD027356.

Size-exclusion chromatography with multi-angle light scattering (SEC-MALS)

SEC-MALS was performed on a HPLC system (Thermo Fisher) connected to a light scattering detector (mini DAWN TREOS, Wyatt). 100 µL of freshly purified protein (concentration 1-2 mg/mL) was injected on a Superdex 75 300/10 GL column (GE Healthcare) at a flow rate of 0.5 mL/min. UV absorption and light scattering were recorded and processed using the ASTRA software (version 6.1, Wyatt).

Circular dichroism (CD)

Far-UV CD was used to assess both the secondary structure and the thermostability of CERT. Purified proteins were clarified by centrifugation and the concentrations adjusted to 2 µM for the full-length constructs, 10 µM for CERT 151-309 and 30 µM for CERT 154-187. CD measurements were carried out using a Chirascan V100 (Applied Biosystems) and 1 mm pathlength quartz cuvettes (Hellma 110-QS). The ellipticity was measured from 195 to 250 nm at 25°C, and the data points were acquired continuously every 1 nm at a speed of 50 nm/min with a digital integration time of 0.5 s and a bandwidth of 1.0 nm. A sample containing buffer only was analysed and subtracted from each signal. The temperature of the 6-cuvette holder was monitored and controlled by a Quantum Northwestern CD 250 Peltier system. Raw spectra were plotted as the mean residue molar ellipticity (θ MRE). Secondary structure was calculated from the BeStSel web server (9, 10).

Intact mass measurements in native-like conditions

To preserve non-covalent interactions, intact mass measurements were performed in native-like conditions by injecting the samples onto MAbPac SEC-1 column (300 Å, 5 µm, 4 x 150 mm, Thermo Fisher Scientific, Sunnyvale, CA, USA) using a Dionex Ultimate 3000 analytical RSLC system (Dionex, Germering, Germany) coupled to a HESI source (Thermo Fisher Scientific, Bremen, Germany). The isocratic separation was performed within 7 min at a flow rate of 300 µL/min and 50 mM ammonium acetate as mobile phase. Eluting fractions were analysed on a high resolution Q Exactive HF-HT-Orbitrap-FT-MS benchtop instrument (Thermo Fisher Scientific, Bremen, Germany). High-mass-range (HMR) mode was activated with 15 000 resolution, in-source CID of 50 eV, AGC target of 5e6 and averaging 2 µscans. The scan range was set to 900-7000 m/z. Data analysis was performed with Protein Deconvolution 4.0 (Thermo Fisher Scientific, Sunnyvale, CA, USA) using the Xtract algorithm with the following settings: 95% fit factor, 25% remainder threshold, signal-to-noise threshold 1.

Structure prediction

A MSA of homologous proteins was produced starting from CERT human sequence (Uniprot Id Q9Y5P4) by searching the whole Uniprot (11) database (downloaded on 2020/09/02) and Metaclust database

(version 2018_06) (12) using phmmer (13) with default parameters. Sequences which presented a fraction of gaps higher than 10% were neglected in the analysis, resulting in a total of 675 available sequences.

The coevolutionary analysis was performed using lbsNN software (14) with one hidden quadratic layer and l2 weight and bias regularisation, both with parameter 0.001. A total of 50 different models were trained and averaged, with the goal of reducing the noise due to the low number of available sequences. In order to avoid effects due to sampling biases, the weight of each sequence in the analysis was computed as the inverse of the number of sequences with more than 90% sequence identity to it. The 10 pairs of residues of the linker region which corresponded with the higher average coevolutionary couplings were compared to the structure obtained by trRosetta (via Robetta web server) (15, 16). The presence of strong couplings between pairs of residues that are very far in the predicted structure suggested the linker region can dimerize in an antiparallel arrangement, and that the dimer is relevant for the correct functioning of the protein. We employed Zdock v3.0.2 (15, 16) in order to predict a set of most probable dimeric arrangements of the structured linker region. Most of the models predicted by Zdock display an antiparallel arrangement of the subunits, in coarse agreement with the results from DCA.

Complementation assays and sphingolipidomics analysis

For sphingolipid labelling assays, patient derived cells, HeLa-WT, and *CERT1*-KO cells were cultured in Dulbecco's Medium (DMEM, Sigma-Aldrich, St. Louis, MO, USA) with 10% FCS. L-serine labelling assay and SPT activity measurements were performed as described elsewhere (17). Briefly, cells were plated in 6-well plates until a confluence of 70 to 80%. Media was exchanged for L-serine free DMEM (Genaxxon Bioscience, Ulm, Germany) containing 10% FBS and 1% penicillin and streptomycin supplemented with isotope-labelled D_3-^{15}N-L-serine (1 mM) (Cambridge Isotope Laboratories, MA, USA) and cells grown for another 16 h. For complementation assays HeLa WT and KO cells were transiently transfected with empty vector or CERT WT or mutant plasmid vectors using Viromer yellow (Lipocalyx GmbH, Germany) transfection reagent. Labelling was performed 48 h post transfection for 16 hours. HPA-12 (10 µM) when used was mixed with the labelling media. DMSO was used as vehicle control for the labelling assay.

Sphingolipidomics analysis of the D_3-^{15}N-L-serine labelled lipids was carried out as described elsewhere (17). Frozen cell pellets were resuspended in 50 µL PBS and extracted with 1 mL Methanol/MTBE (methyl-tert-butyl ether)/Chloroform (MMC) [4:3:3; (v/v/v)] at 37°C (1400 rpm, 30 min). Internal lipid standards include D_7SA (d18:0), D_7SO (d18:1), dhCer (d18:0/12:0), ceramide (d18:1/12:0), glucosylceramide (d18:1/8:0), SM (d18:1/18:1(D_9)), and D_7-S1P. The single-phase supernatant was collected, dried under N_2 and dissolved in 70 µL methanol. Untargeted lipid analysis was performed on a high-resolution Q Exactive MS analyzer (Thermo Scientific) as described earlier (18). LCB analysis of hydrolysed lipids was essentially performed as described (17). LCB were quantified on a QTRAP 6500+ LC-MS/MS System (Sciex).

***Drosophila* husbandry and drug treatment**

Drosophila stocks were maintained on standard fly medium. All flies were reared at 25ºC on a 12 / 12 h light/dark cycle at 50% humidity. Where applicable, 0, 10 or 100 µM HPA-12 (Cayman Chemical Company 28350) in 20% DMSO and 20% food dye (to ensure that the drug was ingested) was applied to the food surface and allowed to dry at room temperature for 2 d prior to use. For morphology rescue, 5 males and 10 females of appropriate genotypes were sorted upon eclosion and transferred to fresh tubes containing 0 or 10 µM HPA-12 for 3 d egglay period before removal and letting larvae develop on 0 or 10 µM HPA-12 until eclosion.

Generation of +WT and +SL flies

The endogenous 5'UTR of *CERT1* followed by the coding sequences of either *CERT1* WT or p.S149L were synthesised (Twist Bioscience) and subcloned into a vector with *mini-white* [w+] selection marker and PhiC31 attB recombination site. Insertions were generated in the AttP40 site on chromosome II, transformants were balanced and then backcrossed with w^{1118} for six generations to produce the transgenic flies used in this study.

Real Time qPCR

Total RNA was isolated from 10 virgin / day post eclosion (dpe) 1 males per condition using QiaShredder and RNeasy kits (Qiagen), according to manufacturer's instructions. Reverse transcription was performed using 250 ng of RNA with random hexamers and SuperScript II (Invitrogen). Real-time PCR was performed on a 7900HT Fast Real-Time PCR system (Applied Biosystems) using PowerUp SYBR Green (Applied Biosystems). Actin (CG12051) and Elongation Factor 1 alpha (CG1873) were used as housekeeping genes; all primer sequences are listed in Table 7. Data shown are normalised to w^{1118} background for total and endogenous dCERT levels, and to $dCERT^{WT}$ for exogenous dCERT level.

***Drosophila* morphology analyses**

Male flies of appropriate genotypes were sorted upon eclosion and transferred to fresh food vials every 2 d. At dpe 7 flies from each genotype were collected and anaesthetised on ice. Images were taken using a stereo microscope (Leica S9i) and analysed using Fiji (ImageJ v1.53c, National Institutes of Health, Bethesda, MD). Whole fly length was measured by drawing a line from the pedicel to the tip of the cercus. Individual body parts from each animal was measured by drawing a line either from the base of the haltere to the tip of the cercus (abdomen length, AL), from the stem of the radial vein (R) to the end of the fourth and fifth branches (R4+R5) of the radial vein (wing length, WL) or from the pedicel to the cervix (head length, HL) as described in Figure 5C. To calculate the head/abdomen ratio, raw head length (HL) was divided by abdomen length (AL) for each animal. For the morphology rescue experiment, body parts were measured on dpe 3 male from each genotype grown on 0 or 10 uM HPA-12.

***Drosophila* immunochemistry**

Brains from adult male flies of appropriate genotypes were dissected in cold 1x PBS and fixed in 4% paraformaldehyde (1 h, RT). Samples were washed multiple times in 1x PBS, PBT (PBS + 0.1% Triton-X100), PBTB (PBT + 0.2% BSA) and then blocked in PBT + 2% normal goat serum (NGS) (30 min, RT). Brains were subsequently incubated one night at 4°C in primary antibody blocking solution followed by an additional night at 4°C for secondary antibody incubation. Mouse anti-Bruchpilot (Developmental Studies Hybridoma Bank (DSHB) at the University of Iowa, Iowa City, IA, nc82, RRID: AB_2314866, 1:100) was used as primary antibody and goat anti-mouse AlexaFluor 488 (Thermo Fisher Scientific, A-11029, RRID: AB_2534088, 1:400) as secondary antibody. After antibody labelling, brains were washed in PBT and mounted with Fluoromount-G® (Southern Biotech, 0100-01). Samples were imaged on a Leica SP8 confocal microscope using a 5x dry objective lens (HC PL Fluotar, 5x/0.15, Leica Microsystems). Optical confocal sections were taken at 1 Airy unit under non-saturated conditions with a resolution of 1024x1024 pixels and frame average 4. Images were then processed using Fiji software and brain volumes were measured using the Fiji 3D object counter (19).

μ-CT

μ-CT of Drosophila heads was performed as described elsewhere (20) with minor modifications. Heads from dpe 5 adult male flies of appropriate genotypes were transferred to a 1.5 mL Eppendorf tube containing 1 mL of 0.5% PBST (1x PBS + 0.5% Triton X-100). Tubes were capped, tapped gently on the bench top and incubated for 5 minutes. Fly heads were then fixed in 1 mL Bouin's solution (5% acetic acid, 9% formaldehyde, 0.9% picric acid; Sigma Aldrich, St. Louis, MO) for 24 h, washed 3×30 min in 1 mL of μ-CT Wash Buffer (0.1 M Na2HPO4, 1.8% Sucrose) and stained with 1 mL of a 0.1 N solution of I2KI (Lugol solution) for 5 d before washing them in two changes of ultrapure water. Stained heads were mounted in a p20 pipette tip filled with water, sealed on both sides with dental resin to prevent evaporation during μ-CT scans and placed in a home-made 3D printed holder.

Samples were scanned using a Scanco μCT 100 cabinet microCT scanner. X-Ray source voltage and current settings: 45 kVp, 200 μA, 9 W power. Native detector settings at a resolution of 3.3 μm and an integration time of 357 ms were used for scans (~4h/head), which resulted in about 1,556 projection images. Frame averaging over 3 projection images was performed to increase signal-to-noise ratio. Following the scan, 3D images were reconstructed using a built-in software package provided by Scanco and exported as DICOM files for postprocessing.

To analyse and segment the brains, Dragonfly (v2021.3, Object Research Systems (ORS) Inc, Montreal, Canada, 2018; software available at http://www.theobjects.com/dragonfly) was used. Dragonfly was operated on a Alienware workstation (Intel(R) Core(TM) i5-7300HQ CPU @ 2.50GHz with 16 CPUs, 16GB RAM, NVIDIA GeForce GTX 1050 Ti with 4 GB available graphics memory). Brains were manually segmented by defining Regions Of Interest (ROIs) using the 2D paintbrush tool over all slices. The 3D segmented brains were then used to compute the volume and were meshed using an unstructured grid for

visualisation. Segmentation of the head was performed using an upper Otsu threshold to select the whole head and calculate the head volume.

***Drosophila* sphingolipidomics**

Crude lysates were prepared by homogenising dpe 1 flies using a Precellys 24 tissue homogenizer (Bertin Technologies, Montigny-le-Bretonneux, France, 6000 rpm, 3 x 30 s) and Zirconia beads in 250 µL of ice-cold PBS. 50 µL of the lysate was aliquoted using wide mouth pipette tips for extraction. Lipid extraction and analysis was performed as above for HeLa cells. Protein concentration was determined by dissolving the extracted pellets in 8M urea followed by Bradford assay.

***Drosophila* Activity Monitor (DAM)**

The locomotion of individual flies was monitored continuously for 7 d in a Drosophila activity monitor (DAM), as reviewed elsewhere (21, 22). Briefly, male flies of appropriate genotypes were sorted upon eclosion and transferred to individual 5 x 80 mm polycarbonate tubes. A small amount of food was inserted at one end and sealed with wax and parafilm to prevent drying out; cotton batting was used to plug the other end. The tubes were then inserted into a MB5 Multibeam Activity Monitor (TriKinetics Inc) where 17 independent infrared beams bisect each tube from top to bottom, allowing the recording of movement at any location within the active length of the tube. As the fly walks back and forth these beams are interrupted in sequence and are recorded as total counts per 30 min. Where applicable, newly eclosed flies were pre-treated for 7 d on 0 or 10 uM HPA-12 prior to loading activity tubes. The apparatus was housed at 29°C on a 12 / 12 h light/dark cycle at 50% humidity.

Negative geotaxis motor ability assay

Male flies of appropriate genotypes were sorted upon eclosion and transferred to fresh food vials every second day. At the indicated age, groups of 4 – 8 animals were transferred to 15 cm long empty glass vials without food, tapped three times, and the ability to climb was video-recorded. The cohort kinematics or mean y-position over time (velocity) for all the flies in each vial was calculated using FreeClimber (23), an open-source Python-based automated quantification platform. At least 50 individuals were analysed for each condition at each time point. To test for seizure susceptibility, groups of 8 – 10 flies were transferred to 9 cm long empty plastic vials without food, agitated for 10s on a VWR vortexer at maximum strength and the ability to climb was video recorded. This procedure was repeated twice more after a 10 min rest interval. The percentage of flies able to reach the top of the vial was calculated at 2, 10, 20 and 30 s. At least 40 individuals were analysed for each condition at each time point.

Longevity Assay

Male and female flies of appropriate genotypes were sorted upon eclosion and transferred to fresh media every second day. Expired animals were counted and removed upon transfer until all flies were dead.

Quantification and Statistical Analysis

Experimental Design. For protein quantification from patient-derived cell lines, we used values from at least three independent experiments. At every stage of the study, the experimenter was blinded to the identity of control and patient-derived cell lines. For example, Experimenter #1 made a list of samples and controls to be tested, and Experimenter #2 randomised this list and re-labeled the tubes; Experimenter #2 was the only person with the key to identify the samples. These samples were then distributed to Experimenter #3 to culture the cells, then to Experimenter #1 to perform western blots, and lastly Experimenters #1 and #4 analysed the data. Only then was the key applied to identify the samples.

Software and statistical analysis. Statistical significance was analysed using GraphPad Prism 8 (https://www.graphpad.com/ scientific-software/prism/) and Excel Software (Microsoft). Statistical details and number of replicates for each experiment can be found in the figures and the legends. The range of expression levels in western blots was determined from at least three independent experiments.

Clinical Appendix

Subject 1 (S93R)

Subject 1 is an 8-year-old Caucasian girl of Dutch descent born after normal pregnancy and delivery at term (40 weeks + 3 days). She was small at birth, with weight and length of 2.4kg and 45cm, respectively. Infantile feeding difficulties were noted. Sleep disturbances were reported during early childhood. Motor, speech, and mild intellectual delays were evident upon examination. She began walking at 16 months with a broad-based gait; she was able to run at 5 years of age.

Behaviorally, she exhibits physical and emotional aggression, head-banging, and features suggestive of ADHD and autism spectrum disorder (ASD). Her behavioral issues require continuous supervision. The parents report short sleep cycles (she falls asleep around 8pm and awakens at around 3am).

At the most recent examination (age 8 years), her IQ was estimated to be 55. Speech was limited to 1-2-word sentences. She remained smaller than average: weight 22.2kg (-2.1 SD), height 118.8cm (0.4 SD). Microcephaly was suspected by her most recent occipitofrontal circumference (OFC) of 47.5cm (-2.5 SD). A brain MRI showed mild encephalopathy although this was attributed to an environmental cause. Variant 4-finger crease was noted in her right hand. Otherwise, the patient was considered to be in good physical health. Whole exome sequencing (WES) of the patient identified the missense variant, c.279T>G (p.S93R), in *CERT1* (NM_005713.3). Parental samples were not available for testing. The inheritance is presumed *de novo* as there is no family history of disease. Despite the fact that this variant is predicted to be weakly pathogenic by *in silico* algorithms and occurs in a weakly conserved position along the protein (PolyPhen2 (HVAR = 0.872)), it is not found in the general population according to gnomad.broadinstitute.org and bravo.sph.umich.edu (assessed September 2021).

Subject 2 (S132L)

Subject 2 is a 24-year-old Caucasian man (Danish ancestry) with a history of developmental delay and infantile-onset febrile seizures. He was born through normal vaginal delivery at term (birthweight: -1 SD). Developmental delay was noted at 8 weeks. Febrile seizures began at 10 months (twice convulsive status epilepticus), and he continued to have seizures until approximately 22 years of age. Seizures were typically focal to bilateral tonic-clonic and impaired consciousness. Due to bouts of severe epilepsy, constant surveillance was needed especially at night. Attacks generally require immediate hospitalization and intensive care. Gross motor delay was apparent from the record of late sitting (42 months) and support-dependent walking at 6 years of age. There is no growth delay but the patient is generally hypotonic.

An IQ score was never formally calculated but is estimated to be below 20 according to the DSM-V scale. A history of behavioral issues include self-injury (arm-biting), which became less frequent at age 15-17 years. His parents report excessive involuntary movements, persistent shaking and a propensity for object mouthing. An absence of eye contact and good visual memory are noted but a formal diagnosis of ASD has never been made. His parents report the patient as having a high pain threshold.

An interpretation of periventricular leukomalacia (PVL) was made from an MRI at age 7 years, but computed tomography (CT) scanning at age 19 years did not show demyelination or other signs of leukodystrophy; rather, it showed moderate central to frontal as well as diffuse cerebral atrophy. Brain EEG waveform patterns showed diffuse slowing, variable localization, no epileptic discharges nor photosensitivity. Together, all tests are consistent with global cerebral atrophy.

At the most recent examination (age 23 years), the patient showed relatively normal growth parameters, but remains non-verbal, still requires support for walking, and show stereotypic features. On a functional level, the patient requires assistance with routine activities such as eating and using the bathroom.

Craniofacial/skeletal features: high forehead, slightly down-slanted palpebral fissures, midface hypoplasia, medial/central flaring of eyebrows, brushy and broad eyebrows, synophrys, downslanting palpebral fissures, swelling lower eyelids, groove below lower eyelids, short nose, anteverted nares, broad/bulbous nasal tip, short columella, long philtrum, cupid's bow upper lip, large ears, hypoplastic antitragus, large ear lobe, pes valgus, hallux varus (sandal gap), broad/short and hammer toes, overriding toe(s), hypoplastic/short nails on toes, broad distal phalanges (toes), and hypermobile DIP-joints in both hands.

Direct sequencing of the *SLC9A6* and *UBE3A* genes was negative for disease-causing mutations. Karyotyping, aCGH, metabolic screening and subtelomeric MLPA were also normal. Whole exome sequencing (WES) of the patient and both parents identified a *de novo* missense variant, c.395C>T (p.S132L) (rs1064794019), in *CERT1* (NM_005713.3) in the patient. This variant is absent in the general population according to gnomAD (gnomad.broadinstitute.org) and BRAVO (bravo.sph.umich.edu), last accessed September 2021. This variant has been reported in at least 5 other individuals with similar clinical features: three individuals in two large case-control studies (24, 25), one individual submitted by geneDX (accession: SCV000567600.3) (https://www.ncbi.nlm.nih.gov/clinvar/variation/419654/) and another included in the present study (Subject 3, see Table 1).

Subject 3 (S132L)

Subject 3 is an 18-year-old Caucasian man of British ancestry born by normal vaginal delivery at 39 weeks (birthweight: 3kg). As an infant, he had feeding difficulties and failed to thrive, sleep disturbance, requiring percutaneous endoscopic gastrostomy (PEG) and fundoplication. He was diagnosed with neonatal global developmental delay and had febrile seizures at 18 months of age, and began having focal and myoclonic seizures from 21 months of age. Fine motor dysfunction includes possible sensory issues, as the patient was unable to grab objects until 6 years of age. Patient was unable to sit without support until 4 years and 6 months and learned to walk with the support at age 8, before becoming immobile at age 12 years. Although an IQ score was never formally calculated, the intellectual disability is clinically estimated to be profound.

Systemically, the patient has a history of gastrointestinal disturbances characterized by episodes of severe constipation and of diarrhea. The cause is uncertain although the patient previously had malrotation

for which he underwent Ladd's procedure. There have also been recurrent urinary tract infections. He has exotropia of the right eye but no other significant visual impairment aside from myopia and astigmatism. The patient has mild scoliosis and osteoporosis. Occipital frontal circumference (OFC) measurement taken at age 13 years was 52.2 cm (2nd percentile) and his weight was 40 kg (25th centile).

A slight prominence of the temporal horn of the right lateral ventricle and a rather generously-sized fourth ventricle with widely open foramina were noted on MRI at 5 years. At age 17 years, the patient continued to have tonic-clonic and absence seizures, with the former requiring hospitalization at times. A brain EEG recording at this age showed diffuse slowing of background rhythm, although no epileptiform activity was noted. His repeat brain MRI performed at 18 years revealed subtle asymmetry in the size of cerebellar hemispheres. Frontal lobes appear small relative to the remainder of the brain, and there is thinning of the corpus callosum, slightly enlarged ventricles, ex vacuao changes and an apparent absence of subcortical white matter throughout (see Supplemental Figure 1B). Currently, at age 18 years, growth parameters are within normal limits but the patient remains immobile and requires complete supervision in daily living. Brachycephaly with occipital flattening and mild turricephaly was also noted.

Craniofacial/skeletal dysmorphia include: sloping forehead, synophrys, short nose, anteverted nares, cupid's bow upper lip, large ears and lobes, prominent ears, uplifted ear lobe, hypoplastic antitragus, temporal narrowing/ narrow front, brachycephaly, occipital flattening, turricephaly, micrognathia, palpebral ptosis, pronounced philtrum, diastema of upper central incisors, lateral thickenings under the lower lip, 3rd-4th fingers syndactyly, gothic palate/ marked alveolar ridges, gum hypertrophy.

Karyotyping, Fragile X repeat expansion testing and aCGH were normal. Whole exome sequencing (WES) of the patient and both parents identified a *de novo* missense variant, c.395C>T (p.S132L) (rs1064794019), in *CERT1* (NM_005713.3). This variant is absent in the general population according to gnomAD (gnomad.broadinstitute.org) and BRAVO (bravo.sph.umich.edu), last accessed September 2021. This variant has been detected in at least 5 other individuals with similar clinical features: three individuals in two large case-control studies (24, 25), one individual submitted by geneDX (Accession: SCV000567600.3) (https://www.ncbi.nlm.nih.gov/clinvar/variation/419654/) and another included in the present study (see Supplemental Table 1 and 2).

Subject 4 (S132L)

Subject 4 is a 13-month-old boy of Dutch descent diagnosed with severe global developmental delay and noted clinical abnormalities at birth. He was delivered at 37 weeks + 6/7 days by uncomplicated cesarean section at a birth weight of 2.97kg (10th-50th percentile). He exhibited feeding difficulties and failure-to-thrive (weight-to-height = -2.1 SD). At 10 months, was evaluated according to the Bayley Scales of Infant Development-III and developmental age was found to be equivalent to 1 month. He exhibited delays in motor milestones (head control=hold at 45 degrees, gross=0.2 months and fine=0.4 months) and as such can neither sit nor walk. His verbal ability is currently limited to making noises. He is generally a quiet and passive infant. Behaviorally, he makes no eye contact or social smile and adopts a typical posture gazing at

his right hand (see photograph on the right). Brain EEG was normal. He is hypertonic and stiff after waking but after a while becomes more active and normotonic. Currently, weight-to-height falls at -2.13 SD, height 0.46 SD and OFC -0.73 SD. Metabolic screening has been normal. There are no apparent systemic issues although there is suspected sensorineural hearing loss and suspected cerebral visual impairment. At the most recent examination, he was noted to have a lock of hypopigmented (white) hair and very dry skin.

Dysmorphic features: prominent forehead, short nose, anteverted nares, midface hypoplasia, cupid's bow upper lip, broad/bulbous nasal tip, hypoplastic alae nasi, palpebral ptosis, swelling lower eyelids, long philtrum, full upper eyelids, full cheeks, protruding columella, everted lower lip, medial/central flaring of eyebrows, epicanthal folds, short palpebral fissures, hammer toes, 2nd-3rd toes proximal syndactyly, temporal narrowing/ narrow front, pronounced philtrum.

Genetic work up included analysis of copy number variation by aCGH which identified a deletion at arr[GRCh37] 7q21.12(87668378_87739123)x1, mat. A trio-exome-based intellectual disability panel (1000 genes) identified *de novo* variant, c.779C>T (p.S132L), in *CERT1* (NM_005713.3). This variant is also pathogenic in Subjects 2, 3, and 5 (Supplemental Table 2).

Subject 5 (S132L)

Subject 5 is a 20-year-old man of German descent with noted developmental delay at 0 months. He was delivered at 36 weeks + 4 days at a birth weight of 3.12kg grams, length of 48 cm (0.71 SD) with no other reported neonatal issues. He was diagnosed with severe intellectual disability and epilepsy at early but unknown age. Infantile cerebral palsy was noted.

At the most recent examination, he appears to be hypotonic and short in stature. His current weight is 46 kg (-2.7kg), height 165 cm (-1.8SD) and OFC 55.5 cm (-0.66 SD). His intellectual disability is severe (estimated IQ range 20-35 according to the DSM-V scale) and he is non-verbal. There was profound motor delay, as he only began to walk, with assistance, at 7 years of age. Currently, his gait is noted to be ataxic. Hypomyelination was noted in neuro-imaging. A cMRI at 4 years of age showed increased subcortical T2-weighted intensity throughout the cerebrum, with occipital dominance (see panel below, and Supplemental Figure 1B). Prominent Virchow-Robin spaces were also noted, some of which were microcystic in the occipital region in both hemispheres. EEG recordings showed seizure susceptibility; he has had one documented seizure. No progression was found in follow-up imaging within the last 10 years. He has high tolerance

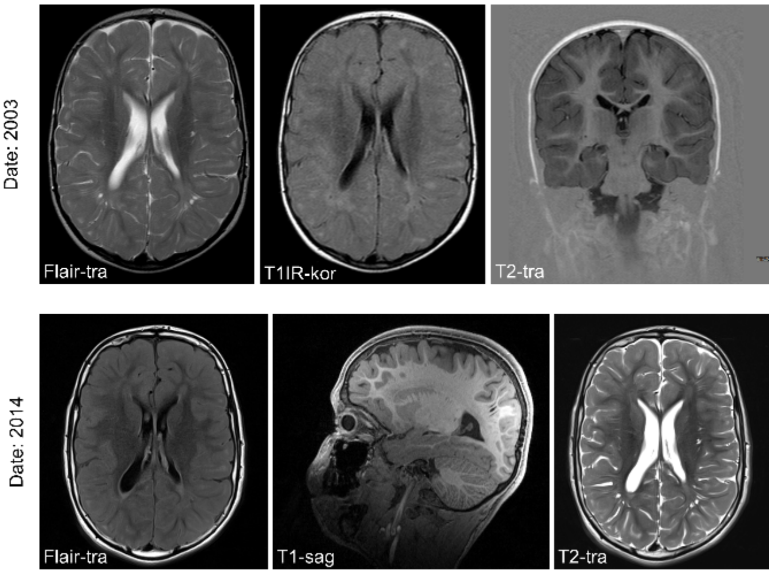

for pain and multiple allergies.

Dysmorphic/skeletal features: high forehead, anteverted nares, cupid's bow upper lip, hypoplastic alae nasi, protruding columella, everted lower lip, hammer toes, flared nares, micrognathia, long face, hallux varus (sandal gap), deep-set eyes, epicanthus, hypoplastic ear lobe, broad/short toes, hypoplastic/short nails on toes, broad eyebrows, short distal phalanx in toes, brachycephaly, occipital flattening, scoliosis.

Trio-based exome sequencing performed in both Subject 5 and his parents identified the *de novo* variant c.779C>T (p.S132L) in *CERT1* (NM_005713.3). This variant is also pathogenic in Subjects 2, 3, and 4 (Supplemental Table 2).

Subject 6 (S135P)

Note: This patient was reported by Murakami et al., (26), and the following clinical details were confirmed by the authors and summarized below.

Subject 6 is a 23-year-old woman of Japanese descent born to unaffected non-consanguineous parents. She was born at full term (gestational age = 37 weeks, 5 days) following an uneventful pregnancy but was small (birth weight = 2.46 kg, height = 44.0 cm, OFC = 33.2 cm). Severe delay in developmental milestones were noted, along with hypotonia and failure to thrive. Although she attained head control at 6 months of age, she was unable to sit without support until age 4 years. She was treated for febrile seizures at age 5, which were believed to have been triggered by a bout of influenza. MRI of the brain at this age showed delayed myelination in the cerebrum and corpus callosum hypoplasia. At 6 years old she was unable to stand or speak meaningful words and her IQ was estimated to be below 35. Follow-up brain MRI at age 23 confirmed leukodystrophy, which was especially apparent in the frontal lobe. Physical examination at this time identified other systemic complications related to growth, central nervous system, and muscular tonus.

Craniofacial/skeletal dysmorphia include: short nose, anteverted nares, diastema of upper central incisors, uplifted ear lobe, midface hypoplasia, groove below lower eyelids, upslanting palpebral fissures, hypertrichosis, bushy eyebrows, hypotelorism, long palpebral fissures, flat nasal bridge, flared nares, short philtrum. She also develop scoliosis and torticollis.

Trio exome sequencing identified a *de novo* variant, c.403T>C, (p.S135P), in *CERT1* (NM_005713.3). This variant is also pathogenic in Subjects 7 (Supplemental Table 2).

Subject 7 (S135P)

Subject 7 is a 2-year, 3-month-old Caucasian girl with developmental delay conceived by intracytoplasmic sperm injection (ICSI) and born from an otherwise healthy bichorial biamniotic pregnancy. She was delivered vaginally at 34 weeks at a birth weight of 1.98 kg (-1.7 SD), length 43 cm (-1.7 SD) and OFC 31 cm (-1.2 SD). She exhibited infantile feeding difficulties due to gastro-esophageal reflux and failure-to-thrive. Examination at 6 months revealed global delay.

Her developmental milestones were thus far are head-holding (3 months) and sitting (19 months). She has not yet begun to walk. There is also apparent speech delay. She is considered to have severe intellectual

disability, though she is still too young to be evaluated for IQ. Behaviorally, she exhibits autistic behaviors such as stereotypic and repetitive behavior and anxiety in response to changes in environment but, unlike some other CerTra patients, shows no sign of aggression or automutilation. At 9 months of age, MRI of the brain showed delay in myelination and dilated ventricles, without abnormalities in head size. EEG of the brain was normal. Neurologically, the patient has not yet had any documented seizures and does not suffer from sleep disturbances. Examination of the fundus was normal although she is astigmatic. There are no apparent neurosensory issues such as hearing loss or abnormal sensitivity to pain.

At her current age she remains short in stature and appears hypotonic. Her most recent growth parameters are weight 9.,4kg (1%), 82 cm (-2 SD) and 47 cm (-1 SD). With the exception of developmental delay and gastro-esophageal reflux, she is otherwise systemically healthy. Blood count, ionogram was normal. TSH and immunoglobulin tests were also normal.

Craniofacial/skeletal dysmorphia include: epicanthus, anteverted nares, low set ears, thin upperlip, micrognathia, buccal hypotonia. Single transverse palmar crease in the hands was also noted.

Family history is negative for significant disease. Fragile X (CGG expansion), array-CGH testing and analysis of the SNRPN locus were negative. Trio genome sequencing of the Subject 7 and both parents identified a *de novo* c.403T>C, (p.S135P) in *CERT1* (NM_005713.3) in the proband. This variant is absent from the general population () and is predicted deleterious by in silico algorithms: M-CAP = 0.108, REVEL = 0.516, CADDv1.6 = 27.9 and Eigen = 0.853. This variant was also reported in Subject 6.

Subject 8 (S138C)

Note: Subject 8 is a case that was included in de Ligt et al. (27) from a large screening for intellectual disability. The following information was summarized from the article along with further clinical details we obtained from the clinicians.

Subject 8 is a 50-year-old Caucasian man of Dutch descent. He was born by vaginal delivery at full term; birthweight was reportedly normal but subsequent infantile feeding difficulties were noted. Development was normal (walking age ~1 year old) until 4 years of age, after which there was a reported regression in speech (few-words) and motor development. Immediate family history was negative except for a nephew (sister's son) who reportedly had pervasive developmental delay-not otherwise specified (PDD-NOS).

At his most recent examination (age 50 years), the patient appears to have moderate intellectual disability (formal IQ score was not calculated but is estimated to be between 36-49 according to DSM-V) and behavioral issues such as general anxiety, aggression, hypersensitivity to touch, and self-injurious behavior, which are consistent with autism spectrum disorder (ASD). His weight is 79.4kg; height is 177.7cm and OFC 55.8cm (20th percentile). Systemically, the patient is generally healthy.

Brain MRI or EEG were performed. There is no history of epilepsy or seizures. Basic metabolic screening, including carbohydrate-deficient transferrin for congenital disorders of glycosylation (CDG), was normal. Mild facial dysmorphia were noted: deeply set eyes, a long face and a prominent forehead.

Fragile X and aCGH testing were normal. Whole exome sequencing identified a *de novo* missense

variant, c.413C>G (p.S138C), in *CERT1* (NM_005713.3). This variant is absent in the general population from both gnomAD (gnomad.broadinstitute.org) and BRAVO (bravo.sph.umich.edu), assessed September 2021. This variant has been detected in another subject in our cohort (Subject 9, see Supplemental Table 2), who is currently only 21 months old but already exhibits anxiety and repetitive behaviors that might develop into an ASD phenotype. Unlike Subject 8, however, Subject 9 experienced developmental delay early in life.

Subject 9 (S138C)

Subject 9 is a 3.5 year-old Caucasian boy of Polish descent. He was born at term (40 weeks) by vaginal delivery with no perinatal complications. He was slightly smaller than average size: birth weight was 3.35 kg (10th – 50th percentile), length 51cm (10th – 50th percentile) and OFC 34 cm (10th – 50th percentile). Microcephaly was noted in infancy. Infantile feeding difficulties were also noted, but this was likely due to an allergy to cow's milk leading to precipitous drop in weight at 3 months. Neurodevelopmental issues and moderate intellectual disability were noted at 6 months. Brain MRI and EEG testing at 13 months were normal.

Self-supported sitting did not occur until 10 months. At 21 months, he shows speech delay and he is hypotonic. General growth parameters are now somewhat above average—weight 12.5kg (90th) and height 87cm (75th percentile)—but OFC at 50cm (90th – 97th percentile) suggests acquired macrocephaly. Repetitive movements were observed and he reportedly suffers from anxiety and timidity in unfamiliar places and need daily support. He also experiences sleep disturbances.

Apparent craniofacial/skeletal dysmorphia include: high and prominent forehead, temporal narrowing/ narrow front, high frontal hairline, hair growth in the lateral parts of the front, retrognathia/micrognathia, cupid's bow upper lip, hypoplastic ear lobe, broad/bulbous nasal tip, full cheeks, lateral thickenings under the lower lip, everted upper and lower lips, medial/central flaring of eyebrows, swelling lower eyelids, groove below lower eyelids, lateral flaring of eyebrows, short columella, prominent ears, uplifted ear lobe, down-slanting palpebral fissures, anteverted nares, long philtrum, hammer toes, hypoplastic/short nails on toes, broad distal phalanges (toes), hallux varus (sandal gap), clubfeet, overriding toe(s), broad/short toes, 2nd-3rd toes proximal syndactyly, short distal phalanx in toes, clubfeet

Whole exome sequencing (WES) of the patient and both parents, performed at a diagnostic laboratory (MEDGEN), identified a *de novo* missense variant, c.413C>G (p.S138C), in *CERT1* (NM_005713.3). This variant is absent in the general population according to gnomAD (gnomad.broadinstitute.org and bravo.sph.umich.edu; assessed September 2021) and is also reported in Subject 8 in this study (Supplemental Table 2).

Subject 10 (T166A)

Note: Subject 10 is a case that was included in Iossifov et al. (28), after screening for autism spectrum disorder. The clinical details are summarized below.

He is a 9-year-old boy with behavioral issues; intellectual disability (VIQ = 54; NVIQ = 47); and

seizures. Short stature and growth delays were not reported. Trio WES identified *de novo* variant c.496A>G (p.T166A) in *CERT1* (NM_005713.3). This variant has been detected in three more individuals enrolled in this study (Subjects 11, 12, and 13, see Supplemental Table 2).

Subject 11 (T166A)

Subject 11 is a 3-year old girl. At the age of one year she presented with motor delay; autism spectrum disorder (ASD); ADHD; mild to moderate intellectual disability (PPVT IQ score = 49). She did not have speech delay, seizures, feeding difficulties, hypotonia, short stature, or gastrointestinal problems. Her brain MRI was normal. Apparent craniofacial dysmorphia include: cupid's bow upper lip, hypoplastic ear lobe, full cheeks, lateral thickenings under the lower lip, medial/central flaring of eyebrows, swelling lower eyelids, groove below lower eyelids, short columella, upslanting palpebral fissures, prominent chin, broad eyebrows.

Molecular Inversion Probe (MIP) screening in the patient and parents identified a *de novo* variant, c.496A>G (p.T166A), in *CERT1* (NM_005713.3), which is also in Subjects 10, 12, and 13.

Subject 12 (T166A)

Subject 12 is a 5-year-old Caucasian boy of German descent delivered by C-section at the 37th week (birth weight 2.7 kg; length 51cm; OFC 33cm) with an otherwise unremarkable pre- and perinatal history. He presented at 10 months with mild motor delay and recurring febrile seizures. The patient learned to sit and walk at 10 and 18 months, respectively. An evaluation at 37 months revealed his developmental age to be 22 months according to the Bayley Scales of Infant Development.

At 5 years of age, he continues to exhibit mild speech delay, although he is able to speak in complete sentences. There are no overt behavioral abnormalities, but he was reported to have difficulty concentrating. In total, the patient suffered from seven febrile seizures (three of which were complicated). Brain MRI and EEG recordings were normal. The most recent physical exam found weight and height in the lower centiles (16 kg, 3rd-10th percentile; 105cm, 3rd-10th percentile) and OFC between the 10th and 25th centile (50 cm). Aside from mild recurrent bronchopulmonal infections, the patient is otherwise healthy.

Mild craniofacial/skeletal dysmorphic features: prominent forehead, hypoplastic ear lobe, full cheeks, medial/central flaring of eyebrows, swelling lower eyelids, upslanting palpebral fissures, prominent chin, skull/facial asymmetry, prominent ears, protruding columella, flared nares, arched eyebrows, overriding toe(s), temporal narrowing/ narrow front.

Metabolic screening, karyotyping and Fragile X repeat expansion testing were normal. A maternally-inherited duplication of 10q24 was found in chromosomal microarray analysis. Direct sequencing of the sodium voltage-gated channel alpha subunit 1 (*SCN1A*) gene, which is associated with febrile seizures, was negative. Whole exome sequencing (WES) of the patient and both parents identified a *de novo* missense variant, c.496A>G (p.T166A) in *CERT1* (NM_005713.3). This variant is absent in the general population according to both gnomAD (gnomad.broadinstitute.org) and BRAVO (bravo.sph.umich.edu), but occurs at a weakly conserved position in the protein and is predicted to be moderately pathogenic by *in silico*

pathogenicity algorithms: CADD = 23.7, Eigen = 0.3585. This variant was found to be pathogenic in three other subjects in the present study (Supplemental Table 2).

Subject 13 (T166A)

Subject 13 is an 9-year-old Caucasian girl of Irish descent who was born with cleft lip and palate at term (39 weeks + 5 days) via normal vaginal delivery. She had several neonatal complications: feeding difficulties, pharyngeal incompetence, and episodes of stiffening at day 2 of life. Feeding difficulties continued even after repair of the cleft lip. At 6 months of age she had abnormal movements with eye-rolling episodes that were self-resolving (likely seizures). By 9 months of age she exhibited cognitive and developmental delays, and by 18 months developed autistic features.

She was able to sit without support at 9 months, crawl at 15 months and walk at 19 months. She did not babble, point, or clap, and further development stalled after 19 months of age. She has some gait ataxia, falls frequently if not supported, and requires a special chair. At age 4, she was formally diagnosed with autism spectrum disorder (ASD) likely because of repetitive movements (head-banging) and obsessive behaviors; she involuntarily clenches at times while alert. She also experience sleep disturbance. At the same age she was diagnosed with epileptic encephalopathy and later on with severe intellectual disability. She appears agitated at times but has a very friendly disposition and social smile; there is no aggressive behavior. She communicates without clear words or gestures by smiling, crying, or staring through the corners of her eyes. She has healthy skin generally with freckles, but has dermatographia (e.g., red marks noticeable when body is gently lifted up, sore after bandages etc.). However, she has a high pain threshold. Hearing assessment was found to be normal although she had grommets inserted to relieve fluid build-up in the ear.

Brain MRI at age 2 years of age was normal although her EEG showed bursts of generalized spike and slow wave activity. She experiences various types of seizures, multiple times throughout the day. Two episodes required admission to the ICU for intubation/ventilation. The range of seizures over the years include jerky movements, myoclonus, absence, tonic seizures, cluster seizures, and status epilepticus. Anti-epileptic drugs have been tried (Phenobarb, Levetiracepam, Valproate, Phenytoin, Ethosuccimide, Topiramate, Clobazam, Clonazepam, Midazolam, Lacosamide, Zonisamide, Rufinamide, Brivaracetam, Topiramate, Gabapentin), as well as a trial of a ketogenic diet, all without much success. Extensive metabolic, genetic and endocrine work-up was unrevealing. A follow-up MRI brain at 8 years of age revealed diffuse supra- and infratentorial brain atrophy and thinning of the corpus callosum, with an appropriate myelination pattern for her age.

Observed craniofacial/skeletal dysmorphia included: prominent chin, high and prominent forehead, temporal narrowing/ narrow front, cupid's bow upper lip, hair growth in the lateral parts of the front, tapering fingers, brachydactyly, prominent ears, lateral flaring of eyebrows, pronounced philtrum, short philtrum, tubular nose, long face, thickening of all parts of the ear (except the earlobe), hammer toes, hypoplastic/short nails on toes, broad distal phalanges (toes), broad/short toes, 2nd-3rd toes proximal syndactyly, short distal phalanx in toes.

Whole genome sequencing (WGS) of Subject 13 and both parents identified a *de novo* variant in the patient, c.496A>G (p.T166A) in *CERT1* (NM_005713.3), which was subsequently confirmed by Sanger sequencing. This variant was found to be pathogenic in three other subjects in the present study (Subject 10, 11, and 12, see Supplemental Table 2).

Subject 14 (G243R)

Subject 14 is an 8-year-old girl of Dutch and Moroccan parentage. The maternal uncle was reported to have had trisomy 13. Perinatal history was unremarkable (gestation: 40+1, Apgar score 7-8-9) although the newborn was on continuous positive airway pressure (CPAP) for a short period of time; birth weight was 3.4 kg (0 SD). She did not exhibit feeding difficulties or failure-to-thrive.

Developmental delay was noted from 4 months of age with emerging behavioral abnormalities including head banging, aggression, wrist-biting, hand-wringing, hand-flapping, severe bruxism in subsequent years. Delays in motor development were apparent in late sitting and walking ages at 15 months and 2 years 8 months, respectively. Developmental regression caused loss of her first words. A formal diagnosis of autism spectrum disorder (ASD) was made along with intellectual disability (estimated IQ range was 20-35 according to the DSM-V scale). She had a febrile seizure at 7.2 years of age (tonic-clonic insult during onset of illness) for which she was prescribed paracetamol (to suppress fevers); episodes of staring have been noted for several years. Patient underwent a 24-hour EEG one month later, which showed asymmetry and frequent epileptic activity with a maximum in the right parietal area Brain MRI/MRS in 2012 and in 2018 after her first epileptic insult did not reveal structural abnormalities but interpretation might have been hindered by movement artefacts. The results were reviewed by a pediatric neuroradiologist who noted no cerebellar abnormalities, normal myelination for her age, normal white matter. Symmetric hyperintense areas at the ventral mesencephalon were noted on Diffusion-Weighted Imaging (DWI), but their significance is unclear and may be to imaging artifacts. A second evaluation of the MRI by a radiologist at Columbia University Irving Medical Center (CUIMC) confirmed normal myelination patterns with the added observation of normal basal ganglia, questionable subtle asymmetry in size of cerebellar hemispheres (right<left), small frontal lobes relative to the remainder of the brain and mild thinning of the corpus callosum. At the most recent examination at age 8 years, the patient exhibited severe intellectual disability and cortical visual impairment (~20% vision estimated). The patient is currently non-verbal. Her cognitive level was estimated to be at 9-11 months of age. The patient has a high pain threshold and skin xerosis. She appears to be hypotonic and petite (-2.18 SD) with growth delay. Her Occipital Frontal Circumference (OFC) is -0.8 SD.

Craniofacial/skeletal dysmorphic features: prominent and high forehead, widow's peak, temporal narrowing front, dolichocephaly, midface hypoplasia, medial/central flaring of eyebrows, synophrys, epicanthal folds, full cheeks, short nose, anteverted and flared nares, short columella, full lips, everted lower and upper lip, cuppid's bow upper lip, diastema of upper central incisors, lateral thickenings under the lower lip, large ears, hypoplastic tragus, hypoplastic ear lobe, broad/short and hammer toes, 2nd-3rd toes proximal

syndactyly, hypoplastic/short nails on toes.

Genetic analyses were performed at age 2 years beginning with direct sequencing of the following candidate genes: *MEF2C*, *MECP2*, *FOXG1*, *CDKL5*, *CLN1* and *CLN2*; no known disease-causing mutations were found. Fragile X testing, aCGH, extensive metabolic and lysosomal tests were normal. Whole exome sequencing (WES) of the patient and both parents identified a *de novo* missense variant, c.727G>A (p.G243R), in *CERT1* (NM_005713.3) (See Sanger sequencing traces). This variant was also detected in Subject 15 in our study but is absent in the general population according to gnomAD (gnomad.broadinstitute.org) and BRAVO (bravo.sph.umich.edu), last accessed September 2021. In 2016, the whole-exome sequencing (WES) result of the patient was published as part of a large study of 2,104 trios that sought to identify genes underlying intellectual disability (29).

Subject 15 (G243R)

Note: The genetic analysis and clinical characteristics of this patients were previously reported in a large case-control study of moderate to severe intellectual disability (30).

Subject 15 is a 17-year-old boy diagnosed with severe global developmental delay, severe intellectual disability, self-mutilation, and aggression. Perinatal history was unremarkable except for feeding difficulties. He reportedly began experiencing febrile seizures at the age of 3 years; unprovoked focal motor seizures occurred at age 5 years, which were treated with clobazam. He did not walk until age 5 years. He has episodes of agitation and self-injurious behavior alternating with periods of apathy, which are treated with risperidone, paroxetine and valproate. He has a left renal agenesis and mild right vesicoureteral reflux. Brain computed tomography (CT) showed mild ventriculomegaly and mild periventricular leukomalacia.

At the most recent examination at the age of 17, he had begun using utensils for eating although he could not get dressed by himself. He has now been off anticonvulsants and seizure-free for several years. His speech was limited to a few words; he understands simple commands. His weight was 53.7 kg (10th percentile), height 163.7 cm (3rd -10th percentile) and head circumference 57.3 cm (98th percentile). He had poor eye contact. Craniofacial/Skeletal features noted during examination included: large/broad distal phalanges in fingers, and broad distal phalanges (toes).

Chromosomal microarray revealed a maternally-inherited 0.069 Mb duplication on chromosome 15q26.3 although his mother was examined and was phenotypically normal. *STXBP1* mutation analysis and metabolic studies (plasma lactate and ammonia measurements, plasma amino acids chromatography) were normal. Whole exome sequencing (WES) of the patient and both parents was performed after which a *de novo* missense variant, c.727G>A, (p.G243R) in the *CERT1* (NM_005713.3) gene was identified. This variant is absent in the general population according to gnomAD (gnomad.broadinstitute.org) and BRAVO (bravo.sph.umich.edu), last accessed September 2021.

Subject 16 (G243E)

Subject 16 is a 5-year-old Caucasian boy of Dutch descent. He was born prematurely (36 weeks) by

vaginal delivery. His birth weight was 2.5kg, length 45cm and occipital frontal circumference (OFC) 33cm. Neonatal feeding difficulties and failure-to-thrive were noted and he was placed on a nasal tube for 1 week. He had early-onset (age 1 year) moderate to severe developmental delay and seizures. Independent (unaided) walking began at 30 months.

His IQ is estimated to be below 55; at 5 years of age, his developmental age was estimated to be approximately 12 months, giving a developmental quotient score of 20 (DQ = CA/DA). Severe speech delay and cerebral visual impairment (CVI) were noted along with myopia. The patient exhibits features of autism spectrum disorder and ADHD. He suffers from insomnia, has difficulty falling asleep and often awakes in the middle of the night (around 3:00AM).

At the most recent examination, weight, height and OFC were +0.5 SD, -2 SD, and -1 SD, respectively. The patient has severe constipation which requires colonic lavage. Metabolic screening showed low amino acid levels. The patient is also IgA-deficient and suffers from frequent infections. His skin has a yellow hue. Focal motor seizures are controlled with oxcarbazepine; EEG recordings detected a normal background rhythm and no epileptic discharges. Relevant findings from brain MRI include delayed myelination.

Craniofacial/Skeletal features noted during examination included: high forehead, micrognathia, medial/central flaring of eyebrows, broad eyebrow, upslanting palpebral fissures, swelling lower eyelids, groove below lower eyelids, broad nasal root, short nose, anteverted and flared nares, broad/bulbous nasal tip, short and protruding columella, thin upper lip, prominent ears, hypoplastic antitragus, uplifted ear lobe, hypoplastic/ short nails on fingers, pes planovalgus.

Array-CGH testing was normal. Whole exome sequencing of the patient and his parents identified a *de novo* variant, c.728G>A (p.G243E), in the *CERT1* gene (NM_005713.3). This variant occurs at a highly conserved position along the protein (PolyPhen2 (HVAR = 0.999). It is absent in the general population according to gnomAD (gnomad.broadinstitute.org) and BRAVO (bravo.sph.umich.edu), last accessed September 2021, and is predicted to be deleterious by various *in silico* pathogenicity algorithms: CADD=27.3, M-CAP=0.023, and Eigen=0.7437.

Subject 17 (T247I)

Subject 17 is a 4-year-old Caucasian girl of Italian descent. She was born at term by vaginal delivery and was on the smaller side: birth weight of 3.18 kg, length of 50 cm and OFC of 33 cm. Her neonatal history noted hypotonia and feeding difficulties that included poor suction and later chewing difficulties, though there was no failure to thrive.

Gross motor milestones were severely delayed. Head-holding was achieved at 18 months and sitting without support at 4 years of age. Cognitive delays were not quantified but she appeared to have severe intellectual disability (estimated IQ range 20-35 according to DSM-V scale) and no verbal ability. She exhibits repetitive hand-washing behavior. There is no history of seizures. Neurosensory issues include increased tolerance to pain, problems with smell and taste and possible unspecified mild hearing impairment. The most recent physical exam noted general hypotonia and low weight for her age (10.1 kg, <3 percentile)

but not short stature (90 cm, 25-50th centile). She still has feeding difficulties. Brain MRI at age 3 years showed hypoplasia of the corpus callosum and abnormality of the pons, diffuse paucity of supratentorial white matter with abnormal signal seen most marked in the periatrial white matter (Supplemental Figure 1B).

Craniofacial/skeletal dysmorphias included: high and prominent forehead, cupid's bow upper lip, protruding columella, micrognathia, long face, hypoplastic ear lobe, broad eyebrows, broad/bulbous nasal tip, full cheeks, epicanthal folds, temporal narrowing/ narrow front, high frontal hairline, bushy eyebrows, lateral thickening under the lower lip, malar hypoplasia, everted upper lip, hypoplastic antitragus, hair growth in the lateral parts of the front, dolichocephaly. The patient also show marked dermatoglyphs on the soles and lumbar kyphoscoliosis.

Initial genetic screening consisted of a diagnostic gene panel for Rett or Rett-like syndromes *MECP2, MEF2C, CDKL5, FOXG1, SMC1A, TCF4, SHANK3, SATB2, STXBP1, SCN1A, SCN2A, SCN8A,GRIN2A, GRIN2B, HCN1, SLC6A1, WDR45, ST3GAL5, MFSD* which was negative. Multiplex ligation-dependent probe amplification (MLPA) of 15q11.2 was normal. Trio exome sequencing of Subject 12 and both unaffected parents identified a *de novo* missense variant, c.740C>T (p.T247I), in *CERT*1 (NM_005713.3). The variant is predicted to be deleterious by several algorithms and is absent from the general population on both gnomAD (gnomad.broadinstitute.org) and BRAVO (bravo.sph.umich.edu), last searched September 2021.

Subject 18 (T251A)

Subject 18 is a one-year-old boy with severe intellectual disability and severe speech delay (per Gesell Development Schedules), growth delay with musculoskeletal issues, and hypotonia. At this young age, there is as yet no history of seizures, visual impairment or hearing loss. No dysmorphic features have been reported. Brain MRI and EEG were normal. Molecular Inversion Probe (MIP) screening in the patient and parents identified a *de novo* variant, c.751A>G, (p.T251A), in the *CERT1* gene (NM_005713.3).

Subject 19 (Y291C)

Subject 19 was identified as having moderate intellectual disability; epileptic seizures; developmental regression. Singleton screening identified c.872A>G (p.Y291C) in *CERT1* (NM_005713.3).

Subject 20 (T296R)

Subject 20 is a 7-year-old African-American boy of Nigerian descent delivered by C-section at 41 weeks + 5 days (birthweight was 3.95 kg, 80th centile). Perinatal history was unremarkable. The patient exhibited normal motor development: head control at 4 months, sitting without support at 6 months, and independent walking at 1 year. The presenting symptom, at two years, was myclonic astatic epilepsy (Doose syndrome). Brain MRI was normal but EEG recordings showed mild irritative activity in temporal areas of both hemispheres. The patient was treated with valproic acid, zonisamide and risperidone and stopped having seizures after 5 years of age. Other neurological issues included non-epileptic paroxysmal events related to

emotional frustration.

At the time of examination (7 years and 8 months), the patient exhibited mild speech delay and mild to moderate intellectual disability, with features consistent with ADHD (restlessness and impulsivity). There were no musculoskeletal abnormalities. Growth parameters were normal: weight 34.7kg (91st percentile), height 129cm (61st percentile) and OFC 55cm (96th percentile). Dry skin (xerosis) was noted.

Observed craniofacial/skeletal dysmorphia included: prominent forehead, high frontal hairline, hair growth in the lateral parts of the front, temporal narrowing/ narrow front, retrognathia/ micrognathia, medial/central flaring of eyebrows, bushy eyebrows, broad eyebrows, arched eyebrows, upslating palpebral fissures, full cheeks, flat nasal bridge, flared nares, broad/bulbous nasal tip, short columella, long philtrum, smooth philtrum, full lips, cupid's bow upper lip, diastema of upper central incisors, prominent chin low-set ears, hypoplastic ear lobe, tapering fingers, few palmar creases, brachydactyly

Serology and metabolic screening were normal. Karyotyping (46, XY) and Fragile X (31 CGG repeats) testing were normal. Array-CGH analysis was also normal. Whole exome sequencing of the patient identified presence of a *de novo* missense variant, c.887C>G (p.T296R), in *CERT1* (NM_005713.3) gene. A maternal sample was not available and the father is deceased. The inheritance is presumed *de novo* as there is no family history of disease. Although this variant occurs at a moderately conserved position along the protein (PolyPhen2 (HVAR = 0.872) and is predicted to be only weakly pathogenic by in silico algorithms, it is not found in the general population according to gnomad.broadinstitute.org and bravo.sph.umich.edu (assessed September 2021).

Subject 21 (V326F)

Subject 21 is a 12-year-old Caucasian girl of Italian and Albanian descent with a history of developmental delay and ataxia. She was born at term (40 weeks and 3 days) by vaginal delivery; perinatal history is unremarkable. Her birth measurements were at the low end of normal: weight was 3.2kg, length was 50cm and OFC 34.5cm. She exhibited infantile feeding difficulties and regression of weight, growth, and OFC at around 8-9 months of age. She had mild motor delay, learning to sit without support at 9 months and walk at 18 months. She has an ataxic gait. At her most recent examination (12 years of age), the patient showed limited bi-syllabic speech and, based on this, was estimated to have moderate intellectual disability. She exhibits repetitive/obsessive hand-washing, self-injurious hand-biting, aggression, ADHD (hyperactivity) and other features consistent with autism spectrum disorder (ASD). Behavioral issues were noted at infancy but grew less frequent by 10 years of age. There is no history of seizures. She is underweight (21 kg, 3rd percentile) and has microcephaly (51 cm), also observed in infancy. Brain MRI was normal (last MRI performed in 2016).

Apparent craniofacial/skeletal dysmorphia include: micrognathia, cupid's bow upper lip, hypoplastic ear lobe, broad/bulbous nasal tip, full cheeks, epicanthal folds, lateral thickenings under the lower lip, everted upper lip, hypoplastic antitragus, dolichocephaly, hypoplastic alae nasi, everted lower lip, deep-set eyes, medial/central flaring of eyebrows, pronounced philtrum, uplifted ear lobe, short philtrum, downslanting

palpebral fissures, tubular nose, broad nasal root, full lips, large ears, hammer toes, hypoplastic/short nails on toes, prominent upper central incisors, broad distal phalanges (toes).

Array-CGH testing identified a 114kb duplication at Xp21.2 (arr Xp21.2(29,584,944x2, 29,594,080 - 20,708,100x3, 29,717,001x2). The duplication includes part of the *IL1RAPL1* gene (exon 6). Quantitative PCR confirmed the duplication to be *de novo*, but aCGH and qPCR are not able to establish if this anomaly is a tandem duplication or an insertional unbalanced translocation. A tandem duplication would cause an effective alteration of the *IL1RAPL1* gene. Unfortunately, the family moved to another country and are not available for further study. Direct sequencing of the *MECP2* and *UBE3A* genes were negative. A gene panel consisting of 74 intellectual disability-autism spectrum disorder genes was performed (full citation below), with negative results. Whole exome sequencing (WES) of the patient and both her parents identified a paternally-inherited missense variant, c.976G>T (p.V326F), in *CERT1* (NM_005713.3), predicted highly deleterious by *in silico* algorithms (PolyPhen2 HVAR = 0.964) and is predicted deleterious by three *in silico* pathogenicity algorithms: CADD = 34, REVEL = 0.37 and Eigen = 0.7073. The variant results in substitution of a highly conserved nucleotide (phyloP: 7.52 [-20.0;10.0]), and is not found in the general population according to gnomad.broadinstitute.org and bravo.sph.umich.edu (assessed September 2021).

Subject 22 (A329P)

Subject 22 is an 8-year-old Caucasian (Spanish) girl of a consanguineous marriage (the father's maternal grandmother and the mother's maternal grandfather were first cousins). It was noted by the 5th month of pregnancy that the fetus was small-for-gestational-age (SGA). Normal vaginal delivery occurred at the 38th week (induced 3 days prior). Birth weight was 2.5 kg (10th percentile), length 48 cm (10th -25th percentile) and OFC 32 cm (10th percentile); neonatal jaundice, facial dysmorphisms, failure-to-thrive and hypoglycemia were noted. Neurological symptoms were first noted at 4 months of age (scarce movement, no smile, inability to fix and follow). The patient was able to sit at 12 months and began walking between 15 months and 2 years of age, but her seizures, despite being controlled by levetiracetam, appeared to interfere with her continued development.

She has severe intellectual disability (does not speak), sleep disturbance, and exhibits various behavioral issues including ADHD/hyperactivity, self-injury (anxiety-induced face-hitting), stereotypy (body-rocking) and other characteristics consistent with autism spectrum disorder (ASD). Frequent vomiting occurred from infancy but stopped after switching to a gluten- and casein-free diet. Due to feeding difficulties continuing from infancy, the patient was placed on an extended regimen of hypercaloric supplementation that seems to have enabled gains in height and head circumference (i.e., skeletal growth) while weight has lagged behind (see table below).

Age	*Weight (percentile)*	*Height (percentile)*	*OFC (percentile)*
Birth	2.5kg (10th)	48cm (10th-25th)	32cm (10th)
3 years, 5 months	12.9kg (5th)	95cm (15th)	48cm (10th)
8 years 3 months	20.5kg (3rd-10th)	127cm (50th)	51cm (25th-50th)

At the last examination (8 years, 3 months), the patient remained hypotonic. Neurosensory testing revealed mild right hypoacusia and mild-moderate left hypoacusia. Hearing loss was also noted in her twin sister, mother (partial) and maternal-grandmother. She also shows delayed visual maturation that is likely linked to her overall developmental delay. Brain MRI revealed delayed myelinization, especially in the frontal and temporal lobes. Brain EEG recordings were largely unremarkable, but a video-EEG during sleep showed scarce epileptiform anomalies in left frontotemporal and temporal regions. Lastly, she has mild tricuspid insufficiency and scalp nevi.

The most recent assessment of craniofacial/skeletal dysmorphia include: high forehead, synophrys, short nose, anteverted nares, cupid's bow upper lip, hypoplastic antitragus, micrognathia, diastema of upper central incisors, uplifted ear lobe, midface hypoplasia, medial/central flaring of eyebrows, groove below lower eyelids, broad/bulbous nasal tip, pes valgus, hair growth in the lateral parts of the front, lateral flaring of eyebrows, arched eyebrows, epicanthal folds, short palpebral fissures, upslanting palpebral fissures, deep-set eyes, hypoplastic alae nasi, smooth philtrum, prominent upper central incisors, low-set ears, hypoplastic helix, hypoplastic ear lobe, short upper limbs (<3rd centile), lower limbs are normal but appear to have scant muscular mass distally, hypoplastic/ short nails on fingers, hypertrichosis, distal scant muscular mass in the lower limbs, broad/short and hammer toes, hypoplastic/short nails on toes, 2nd-3rd toes proximal syndactyly, hypothenar hypoplasia, few palmar creases, low-set and widely-spaced nipples.

Karyotyping was normal. No significant CNV's were detected from aCGH testing except a few polymorphic CNV. Subtelomeric multiplex ligation-dependent probe amplification (MLPA) testing was normal. SAICAR, glycosylation and biotinidase screening were also normal. Direct sequencing of the *MECP2* gene detected a synonymous variant, c.603G>A, p.A201= (rs267608504). The variant is likely benign due to its conservative change and occurrence in the general population at a minor allele frequency of 0.00005. Whole exome sequencing (WES) of the patient and both parents identified variation in two candidate genes. Compound heterozygous variants were detected in the *MOCS2* gene. A *de novo* missense variant, c.985G>C (p.A329P), was identified in *CERT1* (NM_005713.3). This variant is absent in the general population according to both gnomAD (gnomad.broadinstitute.org) and BRAVO (bravo.sph.umich.edu), last assessed September 2021. This variant occurs at a highly conserved position in the protein and is predicted to be highly pathogenic by several *in silico* pathogenicity algorithms: CADD = 32, Eigen = 0.68.

Subject 23 (L330P)

Subject 23 is a 5-year-old girl of Chinese descent with a family history of mild intellectual disability in a cousin. She was born at term (36 weeks and 6 days) following an uncomplicated pregnancy, with birth weight of 2.3kg (10th - 50th percentile), length 50cm (50th percentile) and OFC 32.8cm (10th percentile). Maternal Thyroid Stimulating Hormone (TSH) was elevated before conception, but thyroxine restored normal thyroid lab results during pregnancy. First trimester screening was reported to be low risk. The fetal morphology scan at 20 weeks was normal. Oligohydramnios prior to delivery led to a C-section. The newborn was admitted to the NICU for neonatal pneumonia. Neonatal echo and hearing screening were

normal. Follow up hearing test revealed concerns about hearing in her right ear, the details of which are uncertain. At 6 months of age she was diagnosed with asymmetry and developmental delay.

At age 2 years, her developmental quotient (DQ) was measured to be 37 (25<DQ<39), which falls in the severe range of the evaluation scale developed by the Chinese National Health Commission. She exhibits severe autistic behaviors and aggression. Walking age was 20 months. At 3 years of age, she has a vocabulary of about 5 words. There has been no regression. There are no unusual hand movements. She grabs food but does not feed herself. Sleep is normal.

At 3 years of age her weight was measured to be 12.1 kg (10th percentile), height 88.5cm (10th percentile). At this point, microcephaly was noted, with a measured OFC of 46 cm (2nd percentile). Episodic seizures were noted at the most recent examination. Cardiovascular system and skin were normal. Brain MRI and EEG were normal. She had a unilateral inguinal hernia which was repaired. Urine metabolic screening was normal. At her last examination (5 years) her weight was 19kg (50th-70th percentile), height 115cm (50th-70th percentile), and OFC 46cm (2nd percentile).

Craniofacial/Skeletal features noted during examination: sloping forehead, brachycephaly, occipital flattening, micrognathia, bushy and broad eyebrows, epicanthal folds (ethnic), groove below lower eyelids, anteverted nares, short columella, cupid's bow upper lip, diastema of upper central incisors, gothic palate/ marked alveolar ridges, large ears, hypoplastic and uplifted ear lobe.

Metabolic screening, karyotyping/chromosomal microarray and Fragile X testing were normal. A triplication at chromosome 15, 15q11q11.2 (20262224-22751244)*3, was identified by aCGH. Whole exome sequencing of the patient and parents identified a *de novo* missense variant, c.989T>C, (p.L330P) in *CERT1* (NM_005713.3) gene. This variant is absent in the general population according to gnomAD (gnomad.broadinstitute.org) and BRAVO (bravo.sph.umich.edu), last accessed September 2021. The variant occurs at a highly conserved position along the protein and is predicted to be deleterious across several *in silico* pathogenicity algorithms: M-CAP = 0.084, CADD = 30, REVEL = 0.674 and Eigen = 0.7185.

Subject 24 (R366T)

The little clinical information available on Subject 24 notes him or her to have ADHD; impulsivity; hyperactivity; mild intellectual disability; myopia; high forehead; low-set ears. Singleton screening identified c.1097G>C (p.R366T) in *CERT1* (NM_005713.3).

Subject 25 (I381V)

Subject 25 is a 3-year old boy who recently presented with autism spectrum disorder (ASD); mild intellectual disability; behavioral issues; and mild speech delay.

Molecular Inversion Probe (MIP) screening in the patient and parents identified a *de novo* variant, c.1141A>G, (p.I381V), in the *CERT1* (NM_005713.3) gene. This variant was found in an individual (female) of European (non-Finnish) descent in the general population at a minor allele frequency of 0.00000398 in gnomAD (https://gnomad.broadinstitute.org/variant/5-74695182-T-C?dataset=gnomad_r2_1

assessed April 2020).

Subject 26 (A449V)

Subject 26 is a 2-year, 5-month old girl of Spanish (Mediterranean) descent whose parents also have developmental delay. Clinical abnormalities were noted prenatally. She was born by vaginal delivery at term (40 weeks and 5 days) following an uncomplicated pregnancy but was smaller than average: birth weight was 2.8kg (-1.62 SD), length was 47 cm (-2.15 SD) and head circumference was 33 cm (-1.62 SD). She was hypotonic during infancy but did not exhibit feeding difficulties or failure to thrive. She was diagnosed with ventricular septal defect postnatally.

She has achieved major motor milestones: head-holding (3 months), sitting (10 months) and independent walking (24 months). Speech was limited to approximately 5 discernible words at 2 year and 5 months of age. Behaviorally, she exhibits occasional outbursts and stereotyped/ repetitive movements that are consistent with mild autism spectrum disorder (ASD). She has difficulty falling asleep and precocious awakenings, both of which are partially responsive to melatonin. Gastrointestinal issues include constipation. Recent lab tests revealed anemia. Neuro-imaging was not performed.

As of the most recent examination at 2 years, 5 months, she has made some gains in growth parameters: weight is 13 kg (-0.7 SD), height 88 cm (-1.37 SD) and OFC 48.5 cm (-1.08 SD). Both fine and gross motor skills remain impaired.

She has mild craniofacial/skeletal dysmorphisms: cupid's bow upper lip, brachydactyly, tubular nose, flared nares, full cheeks, medial/central flaring of eyebrows, upslanting palpebral fissures, broad/bulbous nasal tip, swelling lower eyelids, protruding columella, lateral thickening under the lower lip, groove below lower eyelids, hypoplastic alae nasi, hypoplastic/short nails on fingers.

aCGH testing was negative for copy number variants. Singleton whole exome screening identified the variant, c.1346C>T (p.A449V), in *CERT1* (NM_005713.3) which was subsequently found to be inherited from her developmentally delayed mother by Sanger sequencing. A history of schizophrenia was noted in her maternal grandmother in whom genetic screening for this *CERT1* variant is currently being arranged. The c.1346C>T (A449V) variant is predicted to be deleterious by Mutation Taster, LRT and Fathmm and is reported in one healthy male of Latino/American admixture in gnomAD (MAF = 0.000004121).

Subject 27 (P500L)

Subject 27 is a 3.5-year old girl of Caucasian and Filipino descent who was born at term (39 wks+4 days) by vaginal delivery at a birth weight of 3.28kg, length 52cm and OFC of 33cm (3rd-10th centile) with an otherwise unremarkable neonatal history. During early infancy, she suffered from transitory feeding difficulties and exhibited failure to thrive, resulting in a body mass index (BMI) in the 3rd percentile. She was noted to have global developmental delay at 5 months of age.

She was delayed in meeting gross motor milestones: head-holding (10 months) and sitting (14 months). Although she learned to walk at 36 months, she exhibited a wide, possibly ataxic gait. At a recent

examination (3 years and 6 months old), her height was normal for her age (95.4 cm), but she was moderately underweight (11.2 kg, <5th centile); OFC measurement (45.5 cm, <3rd percentile) at this time indicated microcephaly. She had severe intellectual disability (54%, Griffiths scale), was non-verbal and showed stereotypic behavior suggestive of autism spectrum disorder (ASD). There was no history of seizures or sleep disturbances, although she appeared to have a high pain tolerance. A functional heart murmur was also noted although ultrasound tests were normal. She is otherwise healthy.

Trio exome sequencing of Subject 13 and both unaffected parents identified a *de novo* missense variant, c.1499C>T (p.P500L), in the *CERT*1 (NM_005713.3). The variant occurs within the functional START domain of CERT1 and is predicted to be deleterious by several prediction algorithms. It is absent from both gnomAD (gnomad.broadinstitute.org) and BRAVO (bravo.sph.umich.edu), last checked September 2021.

Subject 28 (G312R)

Subject 28 is a 12-year and 10-month old Caucasian boy whose early history was unremarkable. He was born at 40 weeks by vaginal delivery at a birth weight of 3.6 kg and normal birth length and OFC. Sitting and independent walking occurred at 8 and 18 months, respectively. He presented with mild motor and speech delays noted at approximately 2 years of age. His first words were delayed and his ability to use multiple words together occurred between ages 3-4 years. Intellectual disability became apparent over time but is relatively mild. A Wechsler Preschool & Primary Scale of Intelligence-IV (WPPSI-IV) full scale intelligence quotient fell <1 percentile at age 4 years and 6 years. He exhibits no significant behavioral anomalies with the exception of ADHD. As of his most recent examination at the age of 12 years, there has been no apparent regression. He is hypotonic and has constipation and headaches. His weight at age 11-years, 9-months was 29.5kg (z = -1.48), height at age 10-years, 10-months was 139.6cm (z = -0.4) and OFC at 9-years, 2-months was 53.5cm (z = -0.76). A hyper-pigmented macule (2.5 x 0.7 cm) was noted on skin on the abdomen; otherwise, there is no unusual hue.

Mild facial dysmorphia include large ears, deep set eyes, long tubular nose with hypoplastic nasal alae, a thin upper lip (see accompanying frontal and lateral facial photographs). There is an accessory nipple.

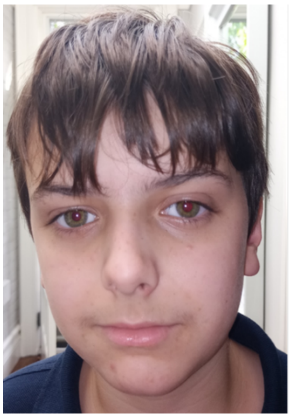

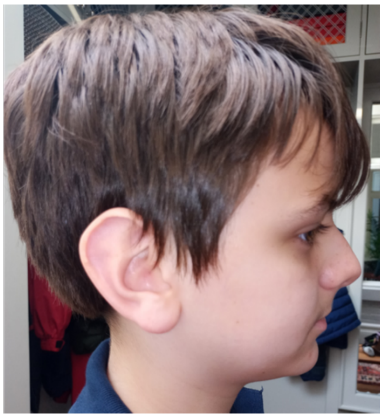

Family history includes a sibling with autism spectrum disorder (level I) and normal cognition, and other family members with dyslexia.

Fragile X and array-CGH screening were negative. A gene panel test of >2,600 genes identified the de novo occurrence the variant, c.934G>C (p.G312R) variant in *CERT1* (NM_005713.3). The variant is absent from gnomAD (https://gnomad.broadinstitute.org/gene/ENSG00000113163?dataset=gnomad_r2_1) and is predicted deleterious by multiple pathogenicity algorithms (CADDv1.6 = 28.9; M-CAP = 0.153).

Subject 29 (L330V)

Subject 29 is a 16-year old girl of Indian descent who was born 36 weeks+5 days by spontaneous vaginal delivery at a birth weight of 2.79 kg (75th – 97th percentile), length 47.5 cm (10th percentile) and OFC of 31.5 cm (3rd percentile). She was noted to be hypotonic. She exhibited motor delays at around 1 year of age but acquired walking by 16 months. At 2 years of age, she was diagnosed with autism spectrum disorder by a psychologist primarily on the basis of stereotyped behaviors, and she had her first seizure, reportedly tonic-clonic. Despite her relatively mild early delays, she has developed severe global developmental delay and severe intellectual disability (she is non-verbal), possibly due to seizures.

Her seizures were treated with levetiracetam (Keppra). At age 11, an EEG study showed epileptiform discharges in the right frontal–temporal region (during sleep), but as of her most recent examination at 15 years of age, she had been seizure-free for the past year.

She continues to exhibit stereotypical behaviors such as hand flapping. She has profound visual field defects in the right eye and is suspected to have cortical blindness, since her anterior and posterior segments appear healthy. External ocular exam revealed right gave vs left field preference, full extraocular movements and visual field full to confrontation. She has maintained her small stature: at age 15, her height was 139.5 cm (<3rd percentile), weight 32.7 kg, (<3rd percentile), OFC 51 cm (3rd percentile). An MRI of the brain at this time showed mild thinning of the anterior part of the corpus callosum with slightly reduced frontal parietal cerebral volume. MR spectroscopy of the ventricles showed a medium-sized lactate peak, of indeterminate significance. A small, incidental arachnoid cyst over the left frontal convexity was noted but not thought to exert any mass effect.

The patient was diagnosed with congenital adrenal hyperplasia (CAH, 21-hydroxylase deficiency); she has occasional abdominal pains and headaches, ambiguous genitalia, and salt wasting. The CAH could also contribute to her small stature, which is proportional (whereas some CerTra patients are underweight for their height). Metabolic testing including plasma acylcarnitine and plasma amino acids were normal. Hgb (low MCV) and K levels are occasionally low.

Family history is reportedly negative for ID or developmental delay syndromes.Genetic screening by BluePrint Genetics identified a *de novo* heterozygous missense variant c.1372C>G, p.(Leu458Val) in *CERT1*, a hemizygous c.955C>T, p.(Gln319*) variant in *CYP21A2*, inherited from her father and a CYP21A2 deletion from her mother. These latter mutations of the gene that encodes the steroid 21-hydroxylase, which is involved in the synthesis of aldosterone and cortisol, is consistent with the congenital adrenal hypoplasia.

Subject 30 (S141R)

Subject 30 is a 14-year-old boy of Caucasian descent who was born at term (38.5 weeks) by vaginal delivery at a birth weight of 7 lbs and 19 inches in length. He presented with global developmental delays between 6 and 8 months of age. He sat at 10 months and walked with an unsteady gait at 17 months, at which time developed seizures. At most recent examination his intellectual disability is estimated to be

severe to profound. He is non-verbal but makes frequent repetitive vocalizations. He exhibits repetitive behavior consistent with ASD, and he is treated with lorazepam (Ativan) for generalized anxiety disorder. He has episodes of emesis when anxious or distressed and hits his chin when irritated.

He has generalized convulsive epilepsy, but seizures are infrequent and controlled with lacosamide (Vimpat) at adjusted doses over the years. The EEG performed in 2008 was normal; repeat EEG (performed a year later in 2009) revealed excessive slowing in the right hemisphere without epileptic activity; a subsequent EEG (performed in 2013) revealed significant dysfunction on the left hemisphere with epileptic tendencies arising from both cerebral hemispheres and an asymmetry on the background with slower frequencies noted over the left hemisphere. The most recently EEG (in 2018) did not reveal any epileptic, focal or diffuse abnormality while the patient was awake, even while having abnormal movements.

His most recent weight was 43.1 kg (11th percentile, Z= -1.25, based on CDC data), height 166 cm (45th percentile, Z= -0.14, based on CDC data), and OFC 54.2 cm (32nd percentile, Z= -0.46, based on Nellhaus data). There is a history of constipation, urinary tract infections with pain and microscopic hematuria. Renal ultrasound and brain MRI have been unrevealing. Metabolic testing showed normal CBC with differential, comprehensive metabolic panel, Vitamin D, thyroid function studies, pyruvic acid, lactic acid, creatine kinase, total and free carnitine, plasma amino acids, acylcarnitine profile, oligosaccharides, mucopolysaccharidosis urine electrophoresis, urine organic acids, plasma and urine GAA levels, and spinal fluid amino acids, lactic acid, and neurotransmitters; plasma and urine creatine levels were elevated but repeat urine testing on a morning void revealed normal urine creatine levels. Global Metabolomic Assisted Pathway Screening (Global MAPs) revealed no significant perturbations of metabolic pathways.

Facial dysmorphisms include palpebral fissures and almond-shaped eyes with mild epicanthal folds bilaterally. Infraorbital creases are noted bilaterally. Flat nasal bridge with short nasal tip. The mouth is typically held open. Pes planus were noted in the feet and hair exhibits normal texture and distribution.

Karyotyping, Fragile X screening and array-CGH screening were negative. A Syndromic Autism panel was normal. Epilepsy panel testing revealed one maternally inherited variant in the *SCN11A* gene that is likely not pathogenic nor clinically significant. Direct sequencing of *ARX*, *MECP2*, *UBE3A* and *SHANK3* were negative. Prader-Willi/Angelman syndrome methylation studies were also negative. Trio exome sequencing identified a *de novo* missense variant, p.S141R, in *CERT*1 (NM_005713.3).

Subject 31 (E424G)

Subject 31 is a 6-year old boy of Indian descent diagnosed with epileptic encephalopathy and developmental delay beginning at 9 months of age. He was born at term (38 weeks) by vaginal delivery at a birth weight of 2.5 kg. Feeding difficulties and failure to thrive were noted during the neonatal and infantile periods. Head-holding occurred at 9 months, sitting at 12 months and walking at 18 months. He exhibits moderate intellectual disability, speech delay (some verbal ability) and motor delays. No regression has been noted. Behaviorally, he exhibits ADHD, aggressive behavior, self-injurious behavior and repetitive movements consistent with autism spectrum disorder. Most recent OFC measurement (47 cm) is consistent

with microcephaly, although we have no information on his height and weight.

Brain MRI was normal. Brain EEG showed generalized epileptiform discharges. Seizures were reported to be generalized tonic clonic (1-2 episodes/week) and myoclonic jerks 5-7 times/day. The patient has been treated with valpraote sodium, levetriacetam, and clobazam and has sleep disturbances. He has exotropia. There are no apparent deviations in pain tolerance. Other than constipation, there are no other significant systemic issues.

Metabolic, blood/serology, hormone (TSH) and urinalysis testing was normal. The face was noted to be long but otherwise not dysmorphic; there are no observable abnormalities of the hands and feet.

Karyotyping, Fragile X screening, array-CGH and gene panel testing were negative. Trio exome sequencing identified a *de novo* missense variant, p.E424G, in *CERT*1 (NM_005713.3).

References

1. Coe BP, Stessman HAF, Sulovari A, Geisheker MR, Bakken TE, Lake AM, et al. Neurodevelopmental disease genes implicated by de novo mutation and copy number variation morbidity. *Nat Genet.* 2019;51(1):106-16.
2. Danecek P, Bonfield JK, Liddle J, Marshall J, Ohan V, Pollard MO, et al. Twelve years of SAMtools and BCFtools. *Gigascience.* 2021;10(2).
3. Chen ZL, Meng JM, Cao Y, Yin JL, Fang RQ, Fan SB, et al. A high-speed search engine pLink 2 with systematic evaluation for proteome-scale identification of cross-linked peptides. *Nat Commun.* 2019;10(1):3404.
4. Schindelin J, Arganda-Carreras I, Frise E, Kaynig V, Longair M, Pietzsch T, et al. Fiji: an open-source platform for biological-image analysis. *Nat Methods.* 2012;9(7):676-82.
5. Hausser A, Storz P, Märtens S, Link G, Toker A, and Pfizenmaier K. Protein kinase D regulates vesicular transport by phosphorylating and activating phosphatidylinositol-4 kinase IIIβ at the Golgi complex. *Nat Cell Biol.* 2005;7(9):880-6.
6. Rappsilber J, Mann M, and Ishihama Y. Protocol for micro-purification, enrichment, pre-fractionation and storage of peptides for proteomics using StageTips. *Nat Protoc.* 2007;2(8):1896-906.
7. Schilling B, Rardin MJ, MacLean BX, Zawadzka AM, Frewen BE, Cusack MP, et al. Platform-independent and label-free quantitation of proteomic data using MS1 extracted ion chromatograms in skyline: application to protein acetylation and phosphorylation. *Mol Cell Proteomics.* 2012;11(5):202-14.
8. Masson GR, Burke JE, Ahn NG, Anand GS, Borchers C, Brier S, et al. Recommendations for performing, interpreting and reporting hydrogen deuterium exchange mass spectrometry (HDX-MS) experiments. *Nat Methods.* 2019;16(7):595-602.
9. Micsonai A, Bulyaki E, and Kardos J. BeStSel: From Secondary Structure Analysis to Protein Fold Prediction by Circular Dichroism Spectroscopy. *Methods Mol Biol.* 2021;2199:175-89.
10. Micsonai A, Wien F, Kernya L, Lee YH, Goto Y, Refregiers M, et al. Accurate secondary structure prediction and fold recognition for circular dichroism spectroscopy. *Proc Natl Acad Sci U S A.* 2015;112(24):E3095-103.
11. UniProt C. UniProt: a worldwide hub of protein knowledge. *Nucleic Acids Res.* 2019;47(D1):D506-D15.
12. Steinegger M, and Söding J. Clustering huge protein sequence sets in linear time. *Nat Commun.* 2018;9(1):2542.
13. Eddy SR. A new generation of homology search tools based on probabilistic inference. *Genome Inform.* 2009;23(1):205-11.
14. Zamuner S, and De Los Rios P. Interpretable Neural Networks based classifiers for categorical inputs. *arXiv [csLG].* 2021.
15. Kim DE, Chivian D, and Baker D. Protein structure prediction and analysis using the Robetta server. *Nucleic Acids Res.* 2004;32(Web Server issue):W526-31.
16. Yang J, Anishchenko I, Park H, Peng Z, Ovchinnikov S, and Baker D. Improved protein structure prediction using predicted interresidue orientations. *Proc Natl Acad Sci U S A.* 2020;117(3):1496-503.

17. Lone MA, Hülsmeier AJ, Saied EM, Karsai G, Arenz C, von Eckardstein A, et al. Subunit composition of the mammalian serine-palmitoyltransferase defines the spectrum of straight and methyl-branched long-chain bases. *Proc Natl Acad Sci U S A.* 2020;117(27):15591-8.
18. Karsai G, Kraft F, Haag N, Korenke GC, Hänisch B, Othman A, et al. DEGS1-associated aberrant sphingolipid metabolism impairs nervous system function in humans. *J Clin Invest.* 2019;129(3):1229-39.
19. Bolte S, and Cordelieres FP. A guided tour into subcellular colocalization analysis in light microscopy. *J Microsc.* 2006;224(Pt 3):213-32.
20. Schoborg TA, Smith SL, Smith LN, Morris HD, and Rusan NM. Micro-computed tomography as a platform for exploring Drosophila development. *Development.* 2019;146(23).
21. Tataroglu O, and Emery P. Studying circadian rhythms in Drosophila melanogaster. *Methods.* 2014;68(1):140-50.
22. Schlichting M, and Helfrich-Forster C. Photic entrainment in Drosophila assessed by locomotor activity recordings. *Methods Enzymol.* 2015;552:105-23.
23. Spierer AN, Yoon D, Zhu CT, and Rand DM. FreeClimber: automated quantification of climbing performance in Drosophila. *J Exp Biol.* 2021;224(Pt 2).
24. Deciphering Developmental Disorders S. Large-scale discovery of novel genetic causes of developmental disorders. *Nature.* 2015;519(7542):223-8.
25. Deciphering Developmental Disorders S. Prevalence and architecture of de novo mutations in developmental disorders. *Nature.* 2017;542(7642):433-8.
26. Murakami H, Tamura N, Enomoto Y, Shimasaki K, Kurosawa K, and Hanada K. Intellectual disability-associated gain-of-function mutations in CERT1 that encodes the ceramide transport protein CERT. *PLoS One.* 2020;15(12):e0243980.
27. de Ligt J, Willemsen MH, van Bon BWM, Kleefstra T, Yntema HG, Kroes T, et al. Diagnostic exome sequencing in persons with severe intellectual disability. *N Engl J Med.* 2012;367(20):1921-9.
28. Iossifov I, O'Roak BJ, Sanders SJ, Ronemus M, Krumm N, Levy D, et al. The contribution of de novo coding mutations to autism spectrum disorder. *Nature.* 2014;515(7526):216-21.
29. Lelieveld SH, Wiel L, Venselaar H, Pfundt R, Vriend G, Veltman JA, et al. Spatial Clustering of de Novo Missense Mutations Identifies Candidate Neurodevelopmental Disorder-Associated Genes. *Am J Hum Genet.* 2017;101(3):478-84.
30. Hamdan FF, Srour M, Capo-Chichi J-M, Daoud H, Nassif C, Patry L, et al. De novo mutations in moderate or severe intellectual disability. *PLoS Genet.* 2014;10(10):e1004772.
